# Mitigation of severe urban haze pollution by a precision air pollution control approach

**DOI:** 10.1038/s41598-018-26344-1

**Published:** 2018-05-25

**Authors:** Shaocai Yu, Pengfei Li, Liqiang Wang, Yujie Wu, Si Wang, Kai Liu, Tong Zhu, Yuanhang Zhang, Min Hu, Liming Zeng, Xiaoye Zhang, Junji Cao, Kiran Alapaty, David C. Wong, Jon Pleim, Rohit Mathur, Daniel Rosenfeld, John H. Seinfeld

**Affiliations:** 1 Research Center for Air Pollution and Health; Key Laboratory of Environmental Remediation and Ecological Health, Ministry of Education, College of Environmental and Resource Sciences, Zhejiang University, Hangzhou, Zhejiang, 310058 P.R. China; 2 Division of Chemistry and Chemical Engineering, California Institute of Technology, Pasadena, CA 91125 USA; 3 College of Environmental Sciences and Engineering, Peking University, Beijing, 100871 P.R. China; 4 Key Laboratory for Atmospheric Chemistry, Chinese Academy of Meteorological Sciences, CMA, 46 Zhong Guan Cun S. Ave., Beijing, 100081 China; 5 Key Lab of Aerosol Chemistry and Physics, SKLLQG, Institute of Earth Environment, Chinese Academy of Sciences, Xi’an, China; 6 Systems Exposure Division, National Exposure Research Laboratory, U.S. Environmental Protection Agency (EPA), Research Triangle Park, NC 27711 USA; 7 Computational Exposure Division, National Exposure Research Laboratory, U.S. Environmental Protection Agency (EPA), Research Triangle Park, NC 27711 USA; 8 Institute of Earth Sciences, The Hebrew University of Jerusalem, Jerusalem, Israel

## Abstract

Severe and persistent haze pollution involving fine particulate matter (PM_2.5_) concentrations reaching unprecedentedly high levels across many cities in China poses a serious threat to human health. Although mandatory temporary cessation of most urban and surrounding emission sources is an effective, but costly, short-term measure to abate air pollution, development of long-term crisis response measures remains a challenge, especially for curbing severe urban haze events on a regular basis. Here we introduce and evaluate a novel precision air pollution control approach (PAPCA) to mitigate severe urban haze events. The approach involves combining predictions of high PM_2.5_ concentrations, with a hybrid trajectory-receptor model and a comprehensive 3-D atmospheric model, to pinpoint the origins of emissions leading to such events and to optimize emission controls. Results of the PAPCA application to five severe haze episodes in major urban areas in China suggest that this strategy has the potential to significantly mitigate severe urban haze by decreasing PM_2.5_ peak concentrations by more than 60% from above 300 μg m^−3^ to below 100 μg m^−3^, while requiring ~30% to 70% less emission controls as compared to complete emission reductions. The PAPCA strategy has the potential to tackle effectively severe urban haze pollution events with economic efficiency.

## Introduction

China’s unprecedented urbanization has been accompanied by an increase in the level of air pollution (both indoor and outdoor), which has been estimated to lead to 2.5 million premature deaths annually^1–4^. To tackle the increased threat owing to the growth of regional air pollution, in 2010 the State Council of China issued the circular, “Regional Joint Prevention and Control of Air Pollution”, to enhance the effort in regional environmental protection and reduction of the overall emissions of air pollutants^5^. The increased frequency of long-duration “haze episodes” with record-breaking air pollutant concentrations has become the most conspicuous feature of air pollution in China^6–9^. A “Haze day” is defined as one with visibility <10 km under conditions of 80% relative humidity and mainly caused by elevated PM_2.5_ (particles with aerodynamic diameter <2.5 μm) concentrations^10–12^. Such severe haze episodes occur predominantly in the economically developed, highly industrialized, and densely populated areas in China, such as the three largest urban regions (i.e., Beijing-Tianjin-Hebei (BTH), Yangtze River Delta (YRD) and Pearl River Delta (PRD)) and six urban mega-cities (i.e., central Liaoning, Shandong Peninsula, Wuhan and its surrounding areas, Chang-Zhu-Tan, Chengdu-Chongqing and Taiwan Strait West Coast)^6–9,13–16^. In January 2013, for example, unprecedented severe haze episodes with peak hourly PM_2.5_ concentrations of ~1000 μg m^−3^ occurred in central eastern China^6,7^enveloping over 10^6^ km^2^. To address severe haze pollution in China, the “Action Plan on Prevention and Control of Air Pollution” (referred to as the “Country Ten” measures), the nation’s most stringent measures to control haze historically, was released by China’s State Council in 2013^17^. The Action Plan aimed, for example, to reduce PM_2.5_ in the Beijing-Tianjin-Hebei region by 25% by 2017 relative to 2012 levels.

Studies of the sources and formation mechanisms of severe PM_2.5_ episodes in China pinpoint emissions from coal-combustion, motor vehicle traffic, construction dust, cooking, and agricultural activities (such as biomass burning), in conjunction with concomitant stagnant meteorological conditions (a shallow atmospheric boundary layer, temperature inversion, low wind speed, and high relative humidity)^6,7,13–15^. In addition to massive amounts of primary emissions, secondary PM products from the oxidation of precursors, such as SO_2_, NO_x_, and volatile organic compounds (VOCs), are estimated to make a significant contribution (30–70%) to PM_2.5_ during these severe haze pollution events^6,7,13–15^. Regional transport of emissions from upwind areas also contributes significantly to haze pollution in the urban areas^13,15^. Despite intensive measures, such as coal combustion reduction, traffic and dust emission controls, significant improvements have not resulted, especially for severe winter haze episodes, as reported by the Xinhua news agency and China’s Ministry of Environmental Protection (MEP)^18–22^. In 2016, for example, 80% of Chinese cities failed to meet air quality standards, and red alerts were triggered (China has a four-tier, color-coded warning system, with red being the most serious, followed by orange, yellow and blue) in more than 20 cities in early January, 2017^21^. The mean PM_2.5_ concentration in 338 Chinese cities in January, 2017, was 78 μg m^−3^, 14.7% higher than in the previous year, especially in the Beijing-Tianjin-Hebei area, where the mean PM_2.5_ concentration was 128 μg m^−3^, 43.8% higher than in 2016^22^.

By contrast, good air quality was achieved for several large international events, such as the 2008 Beijing Olympics, 2010 Shanghai Expo, 2014 Beijing Asia-Pacific Economic Cooperation (APEC) Summit, 2015 Beijing Grand Military Parade, and the 2016 G20 Hangzhou Summit, as a result of stringent urban and regional emission control measures enacted in anticipation of these events^23–27^. These measures involved mandatory temporary closure of most industrial emission sources in the host city and its surrounding areas. For example, it was estimated that the short-term crisis response measures for the 2014 APEC summit resulted in average reduction rates of 39.2, 49.6, 66.6, 61.6, and 33.6% for the emissions of SO_2_, NO_x_, PM_10_, PM_2.5_ and volatile organic compounds (VOCs) in Beijing, respectively^28^. Despite these particular successes, establishing a long-term air pollution control strategy for curbing severe urban haze on a regular basis poses a continuing challenge.

A red alert for severe haze pollution was issued for Beijing on December 8, 2015, extending from 7 am on December 8 until 12:00 am on December 10 (local time) in order to “protect public health and reduce levels of severe air pollution” as stated in Beijing Municipal Environmental Protection Bureau’s official Weibo account^29^. Such a “red alert” is released when severe air pollution with the air quality index (AQI) >500 is forecasted to persist longer than 3 days (72 h). Mandatory and recommended emergency response plans include^30^: (1) Suspending 50% motor vehicle traffic and extending operating hours of public transportation; (2) Barring heavy-duty vehicles from the roads; (3) Banning all construction activities; (4) Washing roads at least once daily to reduce traffic dust; and (5) Banning fireworks and outdoor barbecues. Additional recommended emergency measures include: (1) Schools closed and enterprises encouraged to adopt flexible working schedules and (2) Large-scale outdoor activities banned^30^. Both mandatory and recommended emergency responses triggered by the first-ever smog red alert issued by the Beijing government on December 7, 2015, led only to 10% lower PM_2.5_ concentrations, with the PM_2.5_ concentration in Beijing reaching 233 μg m^−3^ at 5 pm on December 9, despite suspension of production at about 2,100 companies and of outdoor work at ~3,500 construction sites^31^. This was likely a result of the fact that the actual sources contributing to the severe haze during this episode had not been targeted effectively.

Large-scale comprehensive atmospheric chemical transport models are used extensively to evaluate the effectiveness of emission control strategies in a retrospective manner^32^. Source apportionment methods, mainly including emission inventories, 3-D air quality models and receptor models, are used to identify and quantify the major sources of PM and to provide the scientific basis for emission control measures with different shortcomings and uncertainties for each method^32–35^. In this work, we propose a new air pollution control strategy, to be implemented when impending meteorological conditions portend a pollution episode. Using a hybrid trajectory-receptor model in conjunction with a state-of-the-art 3-D atmospheric chemical transport model and high PM_2.5_ concentrations, either observed or forecast, those emission areas that are predicted to most heavily influence air quality levels in the major urban area are identified. We term this a Precision Air Pollution Control Approach (PAPCA), in that the strategy takes advantage of the predictive power of comprehensive atmospheric chemical transport models, offering effectiveness, practicality, and economic efficiency for significantly mitigating impending severe urban haze pollution. To the best of our knowledge, this paper is first to combine all three components (high PM_2.5_ concentrations (either observed or forecast), a hybrid trajectory-receptor model and a comprehensive 3-D air quality model) together to pinpoint the origins of emissions leading to heavy haze events and to optimize emission controls.

## Results and Discussion

The essential idea of the PAPCA is to predict the advent of extreme pollutant concentrations using a comprehensive 3-D air quality model in conjunction with a hybrid trajectory-receptor model to calculate so-called Concentration Weighted Trajectory (CWT) values which can pinpoint the emission areas that are predicted to contribute most significantly to a pending severe urban haze event. The comprehensive 3-D air quality model is employed to optimize the emission controls that will most effectively mitigate the impending haze event. The CWT values are used as a weighting function for emission control factors when the emission control schemes for the targeted areas are optimized in the 3-D atmospheric chemical transport model simulations. In short, the targeted emission areas with the highest potential contributions to the severe haze episode are identified by the CWT values (See Methods).

**Figure 1:**
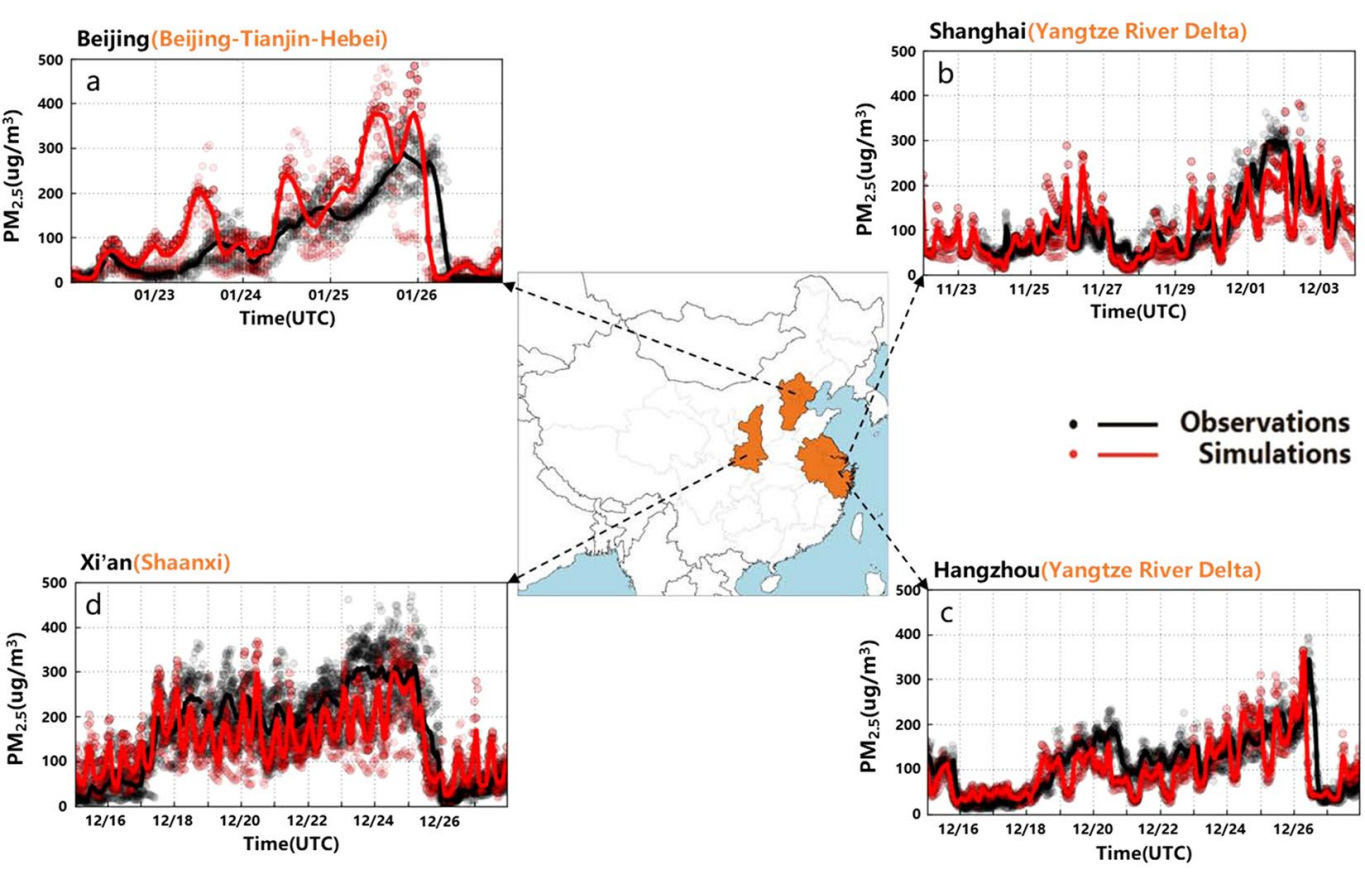
12-km grid resolution model domain (central panel) and time-series comparisons of WRF-CMAQ model predictions and observations for hourly PM_2.5_ concentrations. (**a**) Time-series comparison of predicted and observed hourly PM_2.5_ concentrations at 12 monitoring stations in Beijing from Jan 22 to 26, 2017. (**b**) The same as (**a**) but at 10 monitoring stations in Shanghai for the period from Nov 24 to Dec 4, 2013. (**c**) The same as (**a**) but at 10 monitoring stations in Hangzhou for the period from December 15 to Dec 28, 2013. (**d**) The same as (**a**) but at 13 monitoring stations in Xi’an for the period from December 15 to Dec 28, 2013. The solid lines represent hourly mean concentrations in each city and each dot represents the hourly observation at each surface monitoring station in each city as listed in Table S1.

**Figure 2:**
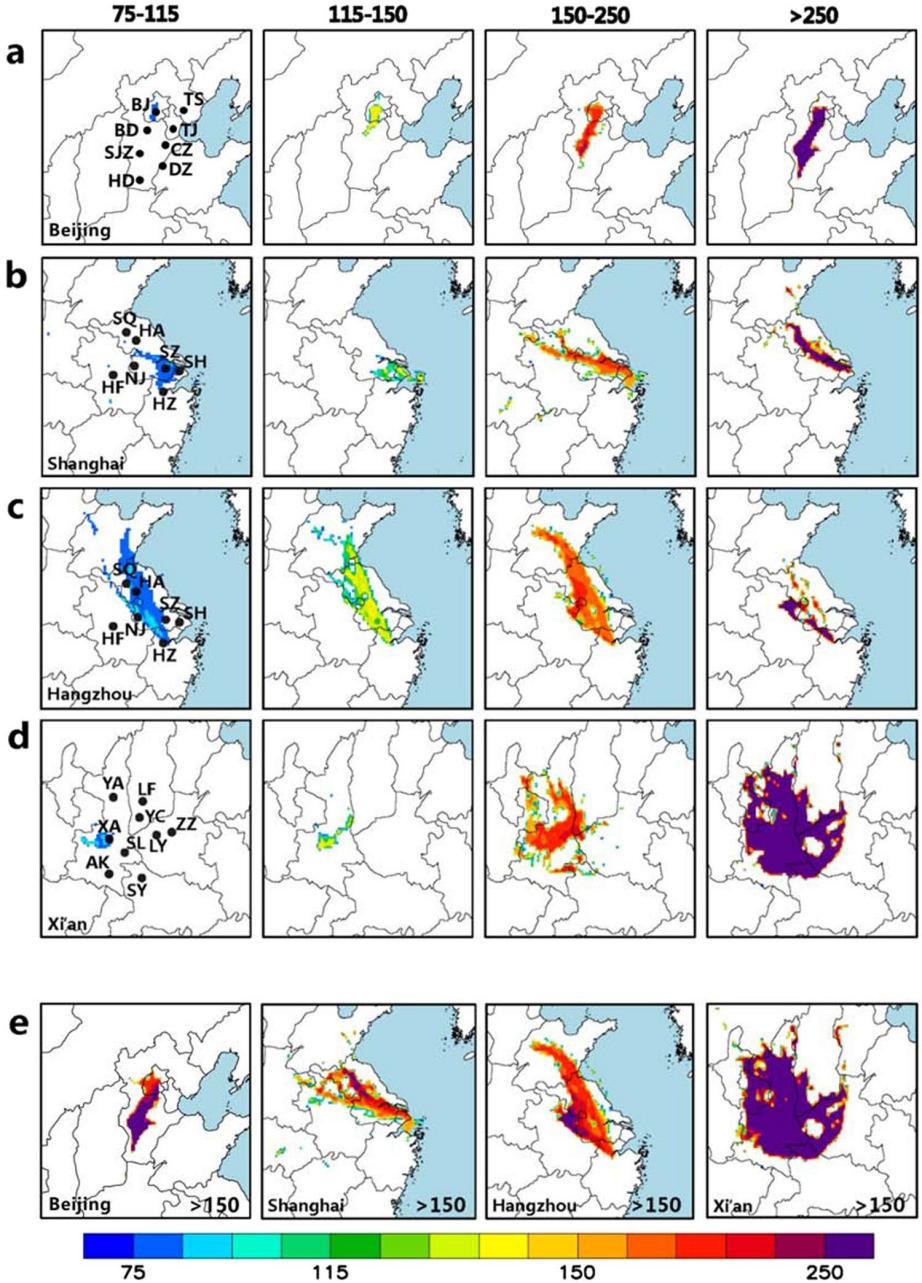
CWT values for PM_2.5_ obtained from the hybrid receptor model to pinpoint origins of heavy haze pollution. (**a**) The spatial distributions of the four different CWT value intervals (75 μg m^−3^ ≤ CWT ≤ 115 μg m^−3^, 115 μg m^−3^ ≤ CWT ≤ 150 μg m^−3^, 150 μg m^−3^ ≤ CWT ≤ 250 μg m^−3^, CWT ≥ 250 μg m^−3^) for PM_2.5_ in Beijing for the period from Jan 24 to 26, 2017. (**b**) The same as (**a**) but for the period from Nov 24 to Dec 4, 2013, in Shanghai. **(c)** The same as (**a**) but for the period from December 15 to Dec 28, 2013, in Hangzhou. **(d)** The same as (**a**) but for the period from December 15 to Dec 28, 2013, in Xian. **(e)** The spatial distributions of the CWT values in the four cities for the cases with CWT ≥ 150 μg m^−3^, AK: Ankang, BD: Baoding, BJ: Beijing, CZ: Cangzhou, DZ: Dezhou, HA: Huai’an, HD: Handan, HF: Hefei, HZ: Hangzhou, LF: Linfen, LY: Luoyang, NJ: Nanjing, SH: Shanghai, SJZ: Shijiazhuang, SL: Shangluo, SQ: Suqian, SY: Shiyan, SZ: Suzhou, TJ: Tianjin, TS: Tangshan, XA: Xi’an, YA: Yan’an, YC: Yuncheng, ZZ: Zhengzhou.

To illustrate how the application of the PAPCA might have worked, we focus on four severe urban haze outbreaks in 2013 in Beijing, Shanghai, Hangzhou, and Xian, which are located in the northern, eastern, eastern and western regions of China, respectively, and one severe urban haze episode in 2017 in Beijing (Figs 1 and S1). Observed hourly peak PM_2.5_ concentrations were 376, 376, 394, and 941 μg m^−3^ for the 2013 outbreaks in Beijing, Shanghai, Hangzhou, and Xian, respectively, each substantially exceeding the daily national PM_2.5_ air quality standard of 75 μg m^−3^. Observed PM_2.5_ concentrations in Beijing rapidly increased from 67 μg m^−3^ at 18:00 on October 26 to 376 μg m^−3^ at 23:00 on October 28 and then sharply decreased to about 20 μg m^−3^ by 3:00 on October 29 (Fig. S1a). PM_2.5_ concentrations rose in a second cycle from 107 μg m^−3^ at 15:00 on October 30 to 355 μg m^−3^ at 15:00 on November 2, then sharply decreasing from ~256 μg m^−3^ at 2:00 to 43 μg m^−3^ at 3:00 on November 3 within a one-hour time frame (Fig. 1a). The 48-h air mass back trajectories (Fig. S2b) show that the severe haze periods in Beijing were influenced mainly by air masses from the southwest and east of Beijing, especially the southwest areas. The eventual sharp decrease of PM_2.5_ in Beijing was associated with a change in air mass wind direction from southwesterly to northwesterly, bringing clean air masses from Inner Mongolia and Mongolia areas to Beijing^30^. Similarly, the haze period in Beijing in 2017 with PM_2.5_ >150 μg m^−3^ was influenced mainly by air masses from the southwest of Beijing (Fig. 1a), and PM_2.5_ concentrations sharply decreased from ~299 μg m^−3^ at 4:00 am on January 26 to ~13 μg m^−3^ at 9:00 am on January 26, 2017, because of a change in air mass wind direction from southwesterly to northwesterly (Figs 1a, 2b and S2a). The severe haze period in Shanghai, lasting from 11:00 on November 30 to 5:00 on December 3 with PM_2.5_ >150 μg m^−3^, was influenced mainly by air masses originating from Zhejiang, Anhui, Jiangsu, Shandong, and Hebei provinces over high emission industrial areas at low wind speed (Figs 1b and S3f)^36^. Similarly, the haze period in Hangzhou with PM_2.5_ >150 μg m^−3^ was influenced predominantly by air masses originating from the northern industrial part of Hangzhou (Anhui, Jiangsu, Shandong, and Hebei provinces) (Figs 1c and S4f)^37^. In contrast to these three cases, the severe haze period in Xian with PM_2.5_ >150 μg m^−3^, by application of this method, is found to be influenced by air masses essentially from all directions (Figs 1d and S5f).

In demonstrating the PAPCA, the two-way coupled WRF-CMAQ model (see Methods and SI) was used to simulate each of these four 2013 severe urban haze episodes in retrospective mode and the 2017 severe urban haze episode in forecast mode. The performance of the WRF-CMAQ model simulations of PM_2.5_, O_3_, SO_2_, NO_2_ and CO for these episodes was evaluated extensively by comparison with observations in each city and related surroundings (see Figs 1 and SI). The model performances for PM_2.5_ chemical composition on the basis of available measurements for the Beijing, Shanghai, Hangzhou and Xian cases in the retrospective simulations are summarized in Tables S6a, S6b, S6c and S6d, respectively, and the temporal variations of comparisons of predictions and observations for each PM_2.5_ component are shown in Figs S15–S18. The model captures with reasonable fidelity the hourly variations and broad synoptic changes in the observed PM_2.5_ concentrations for each of the four cities (Fig. 1, Figs S6–S14, and Tables S2–S5). The model exhibits reasonable performance for PM_2.5_ chemical composition for different heavy haze episodes in different cases (see SI). The normalized mean bias (NMB) values for predictions of PM_2.5_ at Beijing, Shanghai, Hangzhou and Xian are −2.8%, −14.5%, −11.4%% and −11.1%, respectively (Tables S2–S5). The results demonstrate skill in reproducing the urban PM_2.5_, O_3_, SO_2_, NO_2_ and CO concentrations, and PM_2.5_ chemical composition for these haze episodes.

A critical aspect of the development of PAPCA strategies is identification of influential sources and their locations leading to specific severe urban haze episodes. Here, 48-h back trajectories and trajectory cluster analyses are used to locate regional transport pathways and relative contributions of air masses influencing the receptor sites for different periods on the basis of observed PM_2.5_ concentration intervals (see Figs S2–S5 and SI). As expected, the severe urban haze episodes were caused by air masses passing over heavily industrialized areas before arriving at the receptor sites. For example, most of the back trajectories with PM_2.5_ ≥ 150 μg m^−3^ in Beijing (mainly belonging to E-SW and SW clusters) were influenced by the heavily industrialized area southwest of Beijing (Figs S1b(f), S1b(g), S2b(f) and S2b(g)). In Shanghai, the severe haze periods with PM_2.5_ ≥ 150 μg m^−3^ were principally affected by air masses (mainly belonging to NW-S and NW-W clusters) from the industrialized northwest of Shanghai (Figs S3f and S3g). In Hangzhou, upwind air masses came from the industrialized northwest and north of Hangzhou (Fig. S4). As noted above, the severe haze periods in Xian were the results of air masses from all directions (Fig. S5).

To pinpoint the source locations with the largest potential contributions to high concentration values at the receptor site, concentration weighted trajectory (CWT) values for PM_2.5_ are calculated on the basis of the air mass back trajectories and their associated PM_2.5_ concentrations (see Methods). We separated the entire dataset into four different categories on the basis of observed PM_2.5_ concentrations: 75 μg m^−3^ ≤ PM_2.5_ <115 μg m^−3^, 115 μg m^−3^ ≤PM_2.5_ <150 μg m^−3^, 150 μg m^−3^ ≤ PM_2.5_ <250 μg m^−3^, PM_2.5_ ≥ 250 μg m^−3^ and PM_2.5_ ≥ 150 μg m^−3^ when the back trajectories (see Figs S2–S5 and SI) and the CWT values are calculated (see Figs 2 and S1b). The spatial distributions of the four CWT intervals for the five urban cases in Figs 2 and S1b reveal the relative contributions of the potential sources to the high PM_2.5_ concentrations at each of the receptor cities. For example, the main sources affecting the severe haze formation in Beijing with CWT ≥ 250 μg m^−3^ are located in Dezhou, Changzhou, Baoding, Shijiazhuan, Handan, and Tangshang (Fig. S1b), while in Shanghai, they are located in Suzhou, Suqian, Huaian and Bengbu and Nanjing (Fig. 2b). The principal sources affecting Hangzhou with CWT ≥ 150 μg m^−3^ are located in the central part of Jiangsu province (such as Suzhou, Suqian, Huaian, Lianyungang, Bengbu and Nanjing), central part of Shangdong province (such as Rizhao and Jinan) and northern part of Anhui province (Fig. 2c). A close inspection of Figs 2a and S1b for two Beijing cases (one in 2013 and another in 2017) indicates that the main sources affecting the severe haze formation in Beijing with CWT ≥ 250 μg m^−3^ for both cases are located in southwest of Beijing with slightly broader source regions for the 2013 episode. In contrast to the three other cities, the sources affecting haze formation in Xian with CWT ≥ 250 μg m^−3^ are predicted to have originated from all surrounding industrial cities (Fig. 2d). PM_2.5_ concentrations for the haze episode in Xian were consistently >200 μg m^−3^ with the hourly peak PM_2.5_ concentration of 941 μg m^−3^, substantially exceeding the levels in the other three episodes (Fig. 1).

To evaluate the effectiveness of the PAPCA strategy, emission control factors (ECFs) for the domain were calculated on the basis of the CWT values as follows:

1 $${\rm{ECFs}}=\{\begin{array}{c}0.0\,{\rm{if}}\,{\rm{CWT}}\le 75\,{\rm{\mu }}{\rm{g}}\,{{\rm{m}}}^{-3}\\ \frac{{\rm{CWT}}-75}{250-75}\ast 100\,{\rm{if}}\,\,75\,{\rm{\mu }}{\rm{g}}\,{{\rm{m}}}^{-3} < {\rm{CWT}} < 250\,{\rm{\mu }}{\rm{g}}\,{{\rm{m}}}^{-3}\\ \,100\,{\rm{if}}\,{\rm{CWT}}\ge 250\,{\rm{\mu }}{\rm{g}}\,{{\rm{m}}}^{-3}\end{array}$$

**Figure 3:**
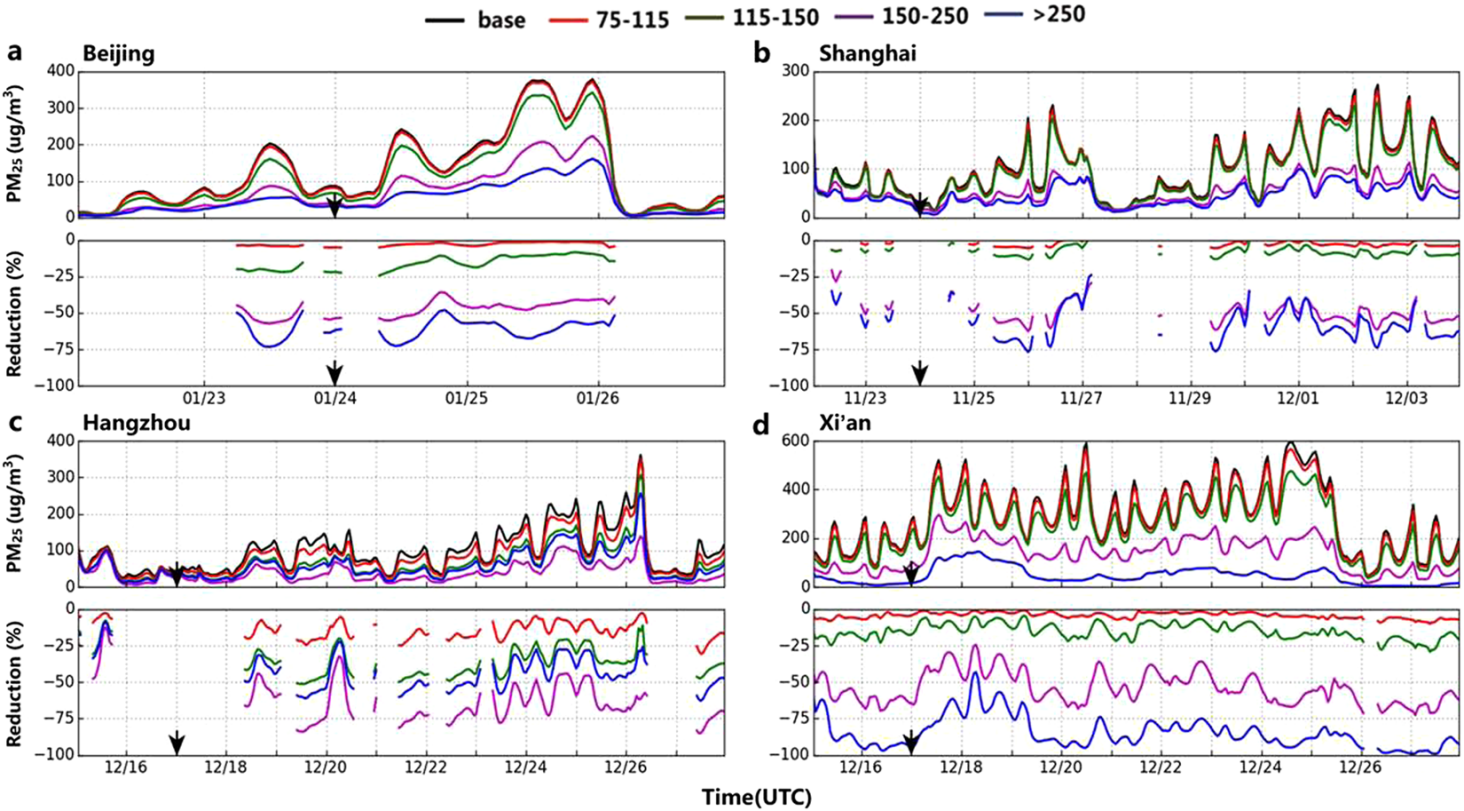
Test of effectiveness of the PAPCA strategy for the four different emission control scenarios. (**a**) Temporal variations of hourly mean PM_2.5_ concentrations and their reduction relative to the base case for the four different emission control scenarios on the basis of the four different CWT value intervals in Beijing for the period from Jan 22 to 26, 2017. The proportional reduction is given only when the hourly mean PM_2.5_ concentrations exceed 75 μg m^−3^. (**b**) The same as (**a**) but for the period from Nov 24 to Dec 4, 2013, in Shanghai. **(c)** The same as (**a**) but for the period from December 15 to Dec 28, 2013, in Hangzhou. **(d)** The same as (**a**) but for the period from December 15 to Dec 28, 2013, in Xi’an. The arrow symbols represent the day with the hourly PM_2.5_ ≥ 150 μg m^−3^ forecasted. The arrow signs show the heavy haze day with hourly mean PM_2.5_ concentration >150 μg m^−3^ at least in one hour and 48 hours earlier than this heavy haze day is the time to start emission control schemes.

Based on the above calculation, all emissions will be shut off or grid cells with CWT ≥ 250 μg m^−3^, while those will remain uncontrolled for grid cells with CWT ≤ 75 μg m^−3^. For grid cells with 75 μg m^−3^ < CWT<250 μg m^−3^, the ECFs are calculated as (CWT-75)/(250–75) with (250–75) as the scaling factor. The concentration of 75 μg m^−3^ is China’s national daily secondary ambient air quality standard for PM_2.5_, which we take as the control objective. To assess the effectiveness of the PAPCA strategy, emission controls were applied to the entire region 48-h prior to the time of the forecasted onset of the severe haze episode (i.e., hourly PM_2.5_ ≥ 150 μg m^−3^) (Fig. 3). Temporal variations and reductions of PM_2.5_ concentrations during the haze periods for the four different emission control scenarios (ECS) on the basis of the CWT value intervals (i.e., ECS1: 75 μg m^−3^ ≤ CWT<115 μg m^−3^; ECS2: 115 μg m^−3^ ≤CWT<150 μg m^−3^; ECS3: 150 μg m^−3^ ≤ CWT<250 μg m^−3^; ECS4: CWT ≥ 250 μg m^−3^) are shown in Figs 3 and S1c.

Model simulations in Figs 3 and S19 show that peak PM_2.5_ concentrations are predicted to be effectively decreased by more than 60% to a level below ~100 μg m^−3^ for each severe urban haze outbreak when the PAPCA strategies are applied to the targeted areas with 150 μg m^−3^ ≤ CWT<250 μg m^−3^ (i.e., ECS3 case) or CWT ≥ 250 μg m^−3^ (i.e., ECS 4 case). By contrast, peak PM_2.5_ concentrations are predicted to be decreased by only <25% if the emission control strategies are applied to the targeted areas with CWT ≤ 150 μg m^−3^ (i.e., cases ECS1 and ECS2) (Figs 3 and S19). For example, the simulated mean PM_2.5_ concentration for the period from 22:00 on October 26 to 16:00 on October 28 in Beijing decreased from 203.0 μg m^−3^ to 198.8, 192.9, 79.8, and 74.0 μg m^−3^ for the emission control scenarios ECS1, ECS2, ECS3 and ECS 4, respectively (Fig. S1c). For the severe haze period from 7:00 on October 31 to 3:00 on November 2 in Beijing, the mean PM_2.5_ concentration decreased from 224.4 μg m^−3^ to 217.0, 205.7, 77.8, and 56.9 μg m^−3^ for the cases ECS1, ECS2, ECS3 and ECS 4, respectively (Fig. S1c). Similar results for the effectiveness of the PAPCA for the severe haze episodes in Shanghai (Fig. 3b), Hangzhou (Fig. 3c), Xian (Fig. 3d) and 2017 Beijing case (Fig. 3a) are obtained (see SI). Fig. S19 summarizes the PM_2.5_ reduction as a function of the emission control scenarios in terms of the CWT value intervals.

To test the practicality of the PAPCA, three emission control scenarios (i.e., cases 1, 3, and 5) were designed by controlling only transportation and industrial emissions instead of all emission sources based on the ECF values as summarized in Table 1. The Beijing government suspended 50% vehicle traffic and production operations at about 2,100 companies in Beijing when the smog red alert was issued on December 7, 2015^18^. Cases 1, 3 and 5 in Table 1 are designed for the PAPCA to control emissions over only targeted areas with CWT ≥ 150 μg m^−3^ (see Fig. 2e) for 50% vehicle emission controls, but different industrial emission control percentages (i.e., 75%, 50% and 25%, respectively), depending on the air quality objectives. CWT ≥ 150 μg m^−3^ is chosen because the results in Fig. 3 show that the peak PM_2.5_ concentrations can be effectively decreased by >60% when the approach is applied to the targeted areas with CWT ≥ 150 μg m^−3^. Case 7 in Table 1 tests the extent to which air quality can be significantly improved when all emissions in the studied city are suspended.

**Table 1:** Emission control scenarios for testing the PAPCA*.

Cases	Transportation	Industry	Controlling Areas
Case1	−50%	−75%	Target Areas
Case2	−50%	−75%	Surrounding Areas
Case3	−50%	−50%	Target Areas
Case4	−50%	−50%	Surrounding Areas
Case5	−50%	−25%	Target Areas
Case6	−50%	−25%	Surrounding Areas
Case7	−100%	−100%	For the city only

^*^Surrounding areas: (1) For Beijing, its surrounding area includes Beijing-Tianjing-Hebei; (2) For Shanghai and Hangzhou, it is Yangtze River Delta (Shanghai-Jiangsu-Zhejiang-Anhui); (3) For Xian, its surrounding area is Shanxi province (see Fig. 1).

Target areas refer to the areas identified by the CWT values in the PAPCA strategy.

**Figure 4:**
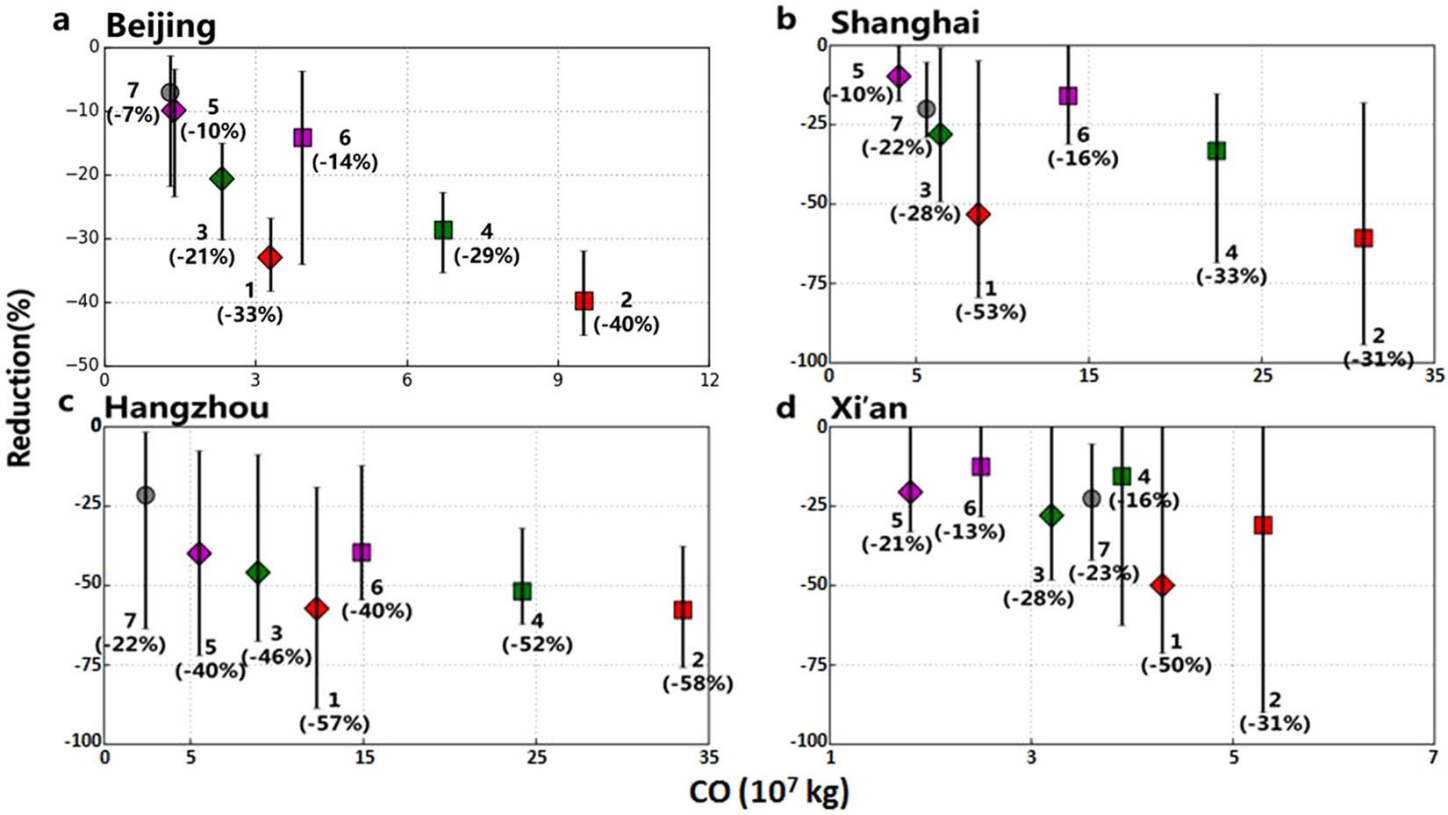
PM_2.5_ reduction percentages as a function of the emission control amounts for the test of economic efficiency of the PAPCA. (**a**) The mean PM_2.5_ reduction as a function of the CO emission control amounts for the 6 different cases in Beijing. Numbers 1–7 refer to the corresponding cases in Fig. S15 and Table 2. The same colors represent the pair comparisons (e.g., cases 1 and 2 are the pair). The ranges of the reduction percentages are calculated on the basis of the hourly results for the periods. Here we use CO emission control amounts as the x-axis to represent the general emission controls because CO is a long-lived tracer of human activity associated with sources, such as combustion, industry, mobile, and hydrocarbon oxidation. (**b**) The same as (**a**) but for Shanghai. (**c**) The same as (a) but for Hangzhou. (d) The same as (a) but for Xian.

Fig. S20a, S20b show the temporal variations and reductions of PM_2.5_ concentrations in the five cities for the different cases, and the results are summarized in Figs 4, S19a and S19b. For case 1 with emission controls from 50% vehicles and 75% industries, the mean PM_2.5_ concentration for the severe haze periods in Beijing (from 22:00 on October 26 to 16:00 on October 28, 2103), Beijing (from 9:00 on January 24 to 3:00 on January 26, 2017), Shanghai (from 10:00 on December 1 to 14:00 on December 3), and Xian (from 9:00 on December 16 to 14:00 on December 25) decreased from 203.0 μg m^−3^ to 95.7 μg m^−3^, 245.9 μg m^−3^ to 166.1 μg m^−3^, 172.1 μg m^−3^ to 71.0 μg m^−3^, and 347.9 μg m^−3^ to 174.4 μg m^−3^, respectively. Since emissions are assumed to be controlled only for two sectors (transportation and industries), the results in Figs 4, S1d, S20a, S20b, S19a and S19b demonstrate the essential nature of the PAPCA. In case 7, for which complete emission control in each city is implemented, the mean PM_2.5_ concentration for the haze periods in Beijing (2013 case), Beijing (2017 case), Shanghai, and Xian are predicted to be decreased from 203.0 μg m^−3^ to 177.9 μg mrespectively. Since emissions are assumed, 245.9 μg m^−3^ to 237.3 μg m^−3^, 172.1 μg m^−3^ to 153.7 μg m^−3^, and 347.9 μg m^−3^ to 268.5 μg m^−3^, respectively. This comparison highlights the fact that, in certain cases, local emissions from the city are not primarily responsible for the severe haze episode, as even when emissions in each city are totally curtailed, mean PM_2.5_ concentrations in Beijing (2013 case), Beijing (2017 case), Shanghai, Hangzhou and Xian are predicted to be reduced only by 11.1%, 7%, 22.0%, 21.5% and 22.6%, respectively (Figs 4 and S1d and Tables 2 and S7).

**Table 2:** PM_2.5_ reduction and emission control amounts for each species for each case.

Case	PM_2.5_ reduction (%)	Emission reduction (10^7^ kg)								
		CO	SO_2_	NH_3_	NO_x_	VOC	PM_2.5_	PMcoarse	BC	OC
**Beijing (2017)**										
1	−33.0	3.3	0.3	0.004	0.4	3.8	0.2	0.08	0.6	0.5
2	−39.7	9.5	1.1	0.01	1.1	10.2	0.5	0.2	1.8	1.7
3	−20.7	2.3	0.2	0.003	0.3	2.7	0.1	0.05	0.5	0.4
4	−28.7	6.7	0.7	0.01	0.9	7.2	0.3	0.2	1.4	1.2
5	−9.9	1.4	0.1	0.002	0.2	1.5	0.05	0.01	0.3	0.2
6	−14.2	3.9	0.4	0.006	0.6	4.2	0.2	0.08	0.9	0.7
7	−7.1	1.3	0.2	0.002	0.3	3.5	0.07	0.04	0.3	0.2
**Shanghai**										
1	−53.2	8.6	13.2	0.4	16.5	35.0	7.8	4.1	0.9	0.8
2	−60.7	30.9	45.9	1.0	65.2	99.4	21.5	12.6	2.8	2.3
3	−28.0	6.4	8.7	0.2	12.9	23.9	5.3	2.7	0.7	0.6
4	−33.2	22.4	30.8	0.7	50.3	68.2	14.8	8.4	2.1	1.6
5	−9.8	4.0	4.5	0.1	9.3	12.9	2.9	1.4	0.5	0.3
6	−15.9	13.8	15.8	0.4	35.3	37.0	8.1	4.2	1.4	1.0
7	−22.0	5.6	1.3	0.1	1.6	2.0	0.3	0.1	0.03	0.03
**Hangzhou**										
1	−38.5	12.3	22.2	0.5	26.1	35.0	8.8	5.0	1.1	0.9
2	−43.3	33.5	49.7	1.1	70.7	107.6	23.3	13.6	3.0	2.4
3	−23.7	8.9	14.9	0.3	20.0	24.1	6.1	3.3	0.9	0.7
4	−33.4	24.2	33.4	0.7	54.5	73.9	16.0	9.1	2.3	1.7
5	−14.5	5.5	7.6	0.2	14.0	13.2	3.3	1.7	0.6	0.4
6	−15.2	14.9	17.1	0.4	38.3	40.1	8.7	4.6	1.5	1.0
7	−21.5	2.4	0.3	0.1	0.7	1.1	0.2	0.1	0.02	0.02
**Xi’an**										
1	−49.9	4.3	14.6	0.7	9.6	11.8	4.3	2.8	0.7	0.9
2	−31.0	5.3	19.6	1.1	10.7	13.5	5.3	3.1	1.0	1.0
3	−27.9	3.2	11.8	0.6	7.5	8.5	3.2	1.8	0.5	0.5
4	−15.6	3.9	13.2	1.0	8.2	9.3	3.6	2.1	0.5	0.6
5	−20.6	1.8	5.0	0.5	5.7	4.6	1.8	0.7	0.4	0.4
6	−12.6	2.5	6.8	0.5	5.8	5.3	1.8	1.1	0.5	0.4
7	−22.6	3.6	0.7	0.4	0.7	0.4	0.4	0.2	0.1	0.1

Cases 2, 4 and 6 in Table 1 are designed to replicate emission control for two sectors (transportation and industries) in each city and its surroundings, as this represents the actual strategy used by the government during the smog red alert^18^. Comparisons between the results of these cases (Cases 2, 4, and 6) and their corresponding cases (Cases 1, 3, and 5) in Figs 4, S1d, S19a, 19b, 20a and 20b and Tables 2 and S7 reveal that relative to those of the Cases 2, 4, and 6, the PAPCA strategy (Cases 1, 3, and 5) can significantly reduce the comparable PM_2.5_ concentrations but with ~30% to ~70% less emission controls. For example, predicted emission reductions needed by the PAPCA for CO, SO_2_, NO_x_, VOC, primary PM_2.5_ (pPM_2.5_), primary coarse PM (PMcoarse), BC and OC in case 1 in Beijing (2013 case) are 23.7, 24.6, 30.2, 27.4, 12.4, 6.3, 1.7, 1.5 10^4^ tons, respectively, about 22% to 26% less than the corresponding values for case 2 (see Table S7). Similar results can be obtained for the Beijing case in 2017 (see Table 2). Note that the significant differences in total emission control amounts between the 2013 and 2017 cases in Beijing as shown in Tables 2 and S7 is due to the fact that the heavy haze episode in 2013 lasted much longer and was affected by broader source regions relative to those in 2017 Beijing case. The emission control times for the Beijing case in 2013 and 2017 are from 00:00 on January 22 to 24:00 on January 26, 2017 (total 120 hours) and from 00:00 on October 24 to 24:00 on November 3, 2013 (total 264 hours). The results for the Beijing case in 2013 retrospective simulations and in 2017 forecast simulations indicate that the PAPCA works well for the same city but under different pollution episodes with the meteorological conditions that are totally different. For Shanghai, predicted emission reductions needed by the PAPCA for CO, SO_2_, NO_x_, VOC, primary PM_2.5_ (pPM_2.5_), primary coarse PM (PMcoarse), BC and OC in cases 1, 3 and 5 are about 64% to 75% less than the corresponding values for the cases 2, 4 and 6, while for Hangzhou city, they are about 55% to 67% less (Table 2). In Xian, the results are summarized for the comparisons in Shaanxi province only because the severe haze episode in Xian was caused by the air masses from the surrounding industrialized cities in all directions (see Figs 2d and S5). Table 2 shows that the PAPCA emission reductions needed for CO, SO_2_, NO_x_, VOC, primary PM_2.5_ (pPM_2.5_), primary coarse PM (PMcoarse), BC and OC in cases 1, 3, and 5 are about 5% to 29% less than the corresponding values for cases 2, 4 and 6 in Xian city.

## Methods

### Precision air pollution control approach (PAPCA)

The PAPCA involves three steps (Fig. S21). The first step is to identify an impending period with high PM_2.5_ concentrations and time to start emission control schemes. Here, we have considered hourly mean PM_2.5_ concentration ≥150 μg m^−3^ to define a severe urban haze event for the city. For example, the arrow signs in Fig. 3 show the heavy haze day with hourly mean PM_2.5_ concentration > 150 μg m^−3^ at least in one hour and 48 hours earlier than this heavy haze day is the time to initiate emission control schemes. The second step is to calculate the concentration weighted trajectory (CWT) values for PM_2.5_ using the hybrid receptor model with 48-h back trajectories and PM_2.5_ concentrations (SI Appendix) to pinpoint source areas leading to high PM_2.5_ levels. Note that the PM_2.5_ trigger concentrations can arise from either observations or model forecasts at the receptor sites when the CWT values are calculated. The third step is to employ a comprehensive atmospheric chemical transport model (CTM) (here the two-way coupled WRF-CMAQ) to optimize emission controls using the CWT values as a weighting function as shown in equation (1). This last step involves a series of CTM simulations to determine the optimal emission control scenarios. In summary, in the PAPCA used in forecast mode, the period of the high PM_2.5_ concentrations is identified by the 3-D CTM forecast, and the CWT values for PM_2.5_ are calculated using the hybrid trajectory-receptor model with 48-h back trajectories on the basis of forecast meteorological fields and forecast PM_2.5_ concentrations. Note that the meteorological initial and lateral boundary conditions were derived from the Global Forecast System (GFS) model data when the PAPCA is used in forecast mode and the meteorological initial, and lateral boundary conditions were derived from the National Center for Environmental Prediction (NCEP) final analysis dataset in the retrospective simulations.

### Observations and hybrid receptor model

Hourly PM_2.5_, O_3_, SO_2_, NO_2_ and CO concentrations at four cities (at 12, 10, 8 and 9 monitoring stations in Beijing, Shanghai, Hangzhou and Xian, respectively) were obtained from the national air quality monitoring network operated and maintained by the Ministry of Environmental Protection (MEP) in China (http://datacenter.mep.gov.cn/). The observations of PM_2.5_ Chemical Composition for each case study were also obtained on the basis of the available field study. More details are available in the SI Appendix.

The 48-h back trajectories starting at the arrival level of 100 m from the monitoring sites were calculated with the NOAA HYSPLIT model (http://ready.arl.noaa.gov/HYSPLIT.php) for each period. Back trajectories were calculated eight times per day at starting times of 00:00, 03:00, 6:00, 09:00, 12:00, 15:00, 18:00 and 21:00 UTC. Based on the results of the HYSPLIT model, the CWT method can be used to pinpoint regional sources with significant potential contributions of high CWT values to receptor site concentrations. The CWT value (*CWT*_*ij*_) for grid cell (i,j) is calculated as the average weighted concentration by the following equation^36–40^:

$$CW{T}_{ij}=\frac{1}{{\sum }_{l=1}^{M}{T}_{ijl}}{\sum }_{l=1}^{M}{C}_{l}{T}_{ijl},$$

where *C*_*l*_ and *T*_*ijl*_ are the concentration at the receptor site on the arrival of trajectory *l* and the time spent in the grid cell (*i, j*) for the trajectory, respectively, and *l* and *M* are the index and total number of the trajectories, respectively. Note that since the targeted areas with the highest potential contributions to the haze episode identified by the CWT values vary for different episodes, the optimal emission control schemes are determined for each particular situation.

### Two-way coupled WRF-CMAQ modeling system

We have used the two-way coupled WRF-CMAQ^41,42^ to simulate urban PM_2.5_ events. A series of simulations for different emission control scenarios were conducted to assess the optimal emission control scheme for the targeted areas. The WRF-CMAQ system was developed by linking Weather Research and Forecast (WRF, version 3.4) and Community Multiscale Air Quality (CMAQ, version 5.0)^41–44^. The model configurations are the same as those in Yu *et al*.^42^. The modeling domain covers most of China and parts of East Asia with 12 km × 12 km grid resolution and with 27 vertical layers for both WRF and CMAQ (see Fig. S6). The physics package of the WRF3.4 (ARW) includes the Kain–Fritsch (KF2) cumulus cloud parameterization, the Asymmetric Convective Model (ACM2) for a planetary boundary layer (PBL) scheme, RRTMG shortwave and longwave radiation schemes, two-moment cloud microphysics, and the Pleim–Xiu (PX) land-surface scheme. In the retrospective simulations, the meteorological initial, and lateral boundary conditions for the outermost domain were derived from the National Center for Environmental Prediction (NCEP) final analysis dataset with a spatial resolution of 1° × 1° and a temporal resolution of 6 h. In the forecast simulations, the meteorological initial and lateral boundary conditions were derived from the Global Forecast System (GFS) model data. The carbon bond chemical mechanism (CB05)^45^ is used to represent gas-phase photochemical reaction pathways, and the AERO6 aerosol module of CMAQ version 5.0 is used to represent aerosol microphysics. The default chemical boundary conditions (BCONs) in the CMAQ model were used in the simulations. For both retrospective and forecast simulations, anthropogenic emissions of SO_2_, NO_x_, CO, NMVOC, NH_3_, PM_10_ and PM_2.5_ over China were based on the Multi-resolution Emission Inventory for China (MEIC)^46^ for 2012 (www.meicmodel.org), while those over the rest of domain were estimated on the basis of Emissions Database for Global Atmospheric Research (EDGAR): HTAP V2 (0.1° × 0.1°). Multi-resolution Emission Inventory for China (MEIC) is a dynamic technology-based inventory for more than 700 anthropogenic emitting sources developed for China covering the years from 1990 to 2013 by Tsinghua University following the work of INTEX-B^46^. With the detailed source classification by representing emission characteristics of different sectors, fuels, products, emission control and combustion/process technologies, the MEIC model can derive emissions which are aggregated to five sectors: power plants, industries, residential, transportation, and agriculture^43^. For example, transportation emissions at high spatial resolution were derived on the basis of vehicle population and emission factors at county level, while the emissions at high-resolution model grids can be derived on the basis of the county-level emissions and information about a digital road map and weighting factors of traveling kilometers and road types^46^. The lumped speciated NMVOC emissions were derived for each source sector by splitting the total NMVOC emissions according to the speciation assignment approaches for different chemical mechanisms such as CB05 in the MEIC43. Temporal variations and gridded emissions were created for each sector using different temporal profiles and spatial aggregations^46^. The detailed description of the MEIC can be found in Li *et al*.^46^. Note that since the heating in southern and northern China is different, the contribution of residential emissions in terms of heating to the haze formation will be significantly different.

### Model performance evaluations

Evaluation of the WRF-CMAQ model performance of PM_2.5_, PM_10_, NO_2_, SO_2_, CO, and PM_2.5_ chemical composition for the studied four severe urban haze episodes for each city are summarized in Tables S2–S6. Figs S7–S18 show time-series comparisons of mean observed and predicted concentrations of different species for each city and its related surrounding cities for the five severe urban haze cases. As can be seen, the model captures the spatial pattern of most of observations reasonably well for this severe haze episode. For the Beijing case in 2013, the NMB values for PM_2.5_ range from 0.2% at Handan to −19.5% at Qinhuangdao at all related cities except Tangshan where the NMB value for PM_2.5_ is 45.8% (see Table S2). For the Shanghai case, the NMB values for PM_2.5_ range from 5.6% at Changzhou to −26.2% at Ningbo (see Table S3). For the Hangzhou case, the NMB values for PM_2.5_ are within ±25% except for Huaian, Huzhou, and Lianyungang where the NMB values for PM_2.5_ are −35.9%, −37.1% and −45.8%, respectively (see Table S4). For the Xian case, The NMB values for PM_2.5_ range from −11.1% at Xian to −37.1% at Tongchuan (see Table S5). Model performances for PM_2.5_ chemical composition on the basis of available measurements for the Beijing, Shanghai, Hangzhou and Xian cases in the retrospective simulations are summarized in Tables S6a, S6b, S6c and S6d, respectively. The temporal variations of comparisons of predictions and observations for each PM_2.5_ component are shown in Figs S15–S18. The model simulations generally underestimate both SO_4_^2−^ and NH_4_^+^ at urban sites (Beijing, Zhengzhou, and Xian sites) but overestimate both SO_4_^2−^ and NH_4_^+^ at the rural sites (Linan, Taiyangshang and Gaolanshan) for all four heavy haze episodes, while the model simulations overestimate EC at all sites and cases except the Beijing case at Zhengzhou site where the model simulations slightly underestimate observed EC by −3.5% (see Table S6a). The model simulations underestimate OC at all sites and cases except the Shanghai case at Taiyangshan site where the model simulations slightly overestimate observed OC by 13.3% (see Table S6b), while the model simulations underestimate NO_3_^−^ at all sites and cases except the Beijing case at the Beijing site and Xian case at Xian site where the model simulations overestimate NO_3_^−^ slightly. Uncertainties in emission inventories, the physical-chemical mechanisms of haze formation, and prognostic model simulation of meteorological fields are the sources of biases in the simulations of PM_2.5_ chemical composition. More details about model performance are available in the SI Appendix.

### Uncertainties associated with the PAPCA strategy

There are recognizable uncertainties associated with the PAPCA strategy. First, simulations of the severe haze episodes (i.e., high PM_2.5_ concentrations) by the 3-D air quality models (WRF-CMAQ here) involve inevitable uncertainties in emission inventories^32^, the physical-chemical mechanisms of haze formation, and prognostic model forecasts of meteorological fields. Emissions-modeling uncertainties involve inventory emissions for area, line and point sources, temporal profiles, chemical speciation profiles, spatial surrogates and vertical distribution^47^. Although the anthropogenic emission inventory for China used in this study was derived from the extensively tested state-of-the-art MEIC model (includes five anthropogenic source sectors: power, industry, transportation, residential, and agriculture)^48^, uncertainties remain at high spatial and temporal resolutions. Severe winter haze events over the North China Plain have not been captured successfully by a number of air quality models, indicating that the physical-chemical mechanisms of haze formation may not yet be completely understood^49^. Despite the overall strong performance of the two-way coupled WRF-CMAQ model for these five severe urban haze episodes, further in-depth evaluation of model predictions is warranted.

Uncertainties in the hybrid receptor model can affect the accuracy in determination of source origins of severe haze pollution and in optimization of the emission control schemes for the targeted areas. Despite the fact that the HYSPLIT model is a well-tested system for computing air parcel back-trajectories^50^, complex wind fields can lead to uncertain prediction of trajectories because of neglect of wind shear in the trajectory calculations^51^.Nevertheless, it is established that the trajectory model may be accurately employed for various regimes of stability, wind shear and source configuration^51^. Since the high PM_2.5_ concentrations are the result of primary emissions and secondary aerosol production, the CWT values, which are calculated on the basis of PM_2.5_ concentrations at the receptor site and the arrival of back-trajectories (see Methods), contain the information about the sources of primary emissions and chemical transformation. That implementation of the PAPCA is predicted to effectively reduce the PM_2.5_ peak concentrations for the five severe urban haze episodes using the CWT values as weighted function suggests that the CWT values successfully pinpoint the source origins of the severe urban haze pollution.

## Electronic supplementary material

Supporting Information
