# Supplementary material for: Mitigation of severe urban haze pollution by a precision air pollution control approach
Source: Sci Rep. 2018 May 25;8:8151. doi: 10.1038/s41598-018-26344-1 (PMC5970218; doi:10.1038/s41598-018-26344-1)
Supplement: Supplementary file 1 — Supporting Information [file 41598_2018_26344_MOESM1_ESM.pdf]

Supporting Information for mitigation of severe urban haze pollution by a precision air pollution control approach

Shaocai Yu*, Pengfei Li*, Liqiang Wang*, Yujie Wu， Si Wang, Kai Liu, Tong Zhu, Yuanhang Zhang, Min Hu, Liming Zeng, Xiaoye Zhang, Junji Cao, Kiran Alapaty, David Wong, Jon Pleim, Rohit Mathur, Daniel Rosenfeld, and John H. Seinfeld

Correspondence and requests for materials should be addressed to S.Y. (email: shaocaiyu@zju.edu.cn or shaocaiy@caltech.edu) or J.H.S. (email: Seinfeld@caltech.edu) or K.A. (email: Alapaty.Kiran@epa.gov)

Observational data. Detailed observations of hourly air pollutant ($PM_{2.5}$, PM_{10}, O_3, SO_2, NO_2, and CO) concentrations for the four severe urban haze episodes in retrospective simulations are available at the website of Ministry of Environmental Protection in China (http://datacenter.mep.gov.cn/). Table S1a lists information about the monitoring stations in each city considered in this study. Measurements at different sites in the same city were analyzed together because the $PM_{2.5}$ concentrations at these monitoring sites in the same city are close, as shown in Figure 1. Table S1a also lists information about the observations used to evaluate the model performance for each study case. For instance, the observations at Beijing and its surrounding 12 cities for the Beijing case (Cangzhou, Handan, Langfang, Shijiazhuang, Tianjin, Zhangjiakou, Baoding, Chengde, Hengshui, Qinhuangdao, Tangshan, Xingtai) are used. For the Shanghai case, the observations at Shanghai and its surrounding 9 cities (Hangzhou, Jiaxing, Nantong, Suzhou, Changzhou, Huzhou, Nanjing, Ningbo, Wuxi) are used. For the Hangzhou case, the observations at Hangzhou and its surrounding 9 cities (Huaian, Laiwu, Linyi, Yangzhou, Changzhou, Huzhou, Lianyungang, Nanjing, Zhenjiang) are used. For the Xian case, the observations at Xian and its surrounding 5 cities (Baoji, Tongchuan, Weinan, Xianyang and Yanan) are used.

For observations of $PM_{2.5}$ chemical composition, Table S1b lists the information about the monitoring stations from which the observational chemical composition of $PM_{2.5}$ is used to evaluate the model performance for each study case. For the Beijing case from Oct 27 to Nov 3, 2013, an Aerodyne high resolution time-of-flight aerosol mass spectrometer was used to measure the chemical compositions of $PM_{2.5}$ and black carbon was measured by a

single-wavelength (670 nm) Thermo multiangle absorption photometer [6]. For the Xian case from Dec 15 to 28, 2013, the sulfate, nitrate, ammonium, and organic aerosols are measured by the Aerodyne High Resolution Time-of-Flight Aerosol Mass Spectrometer (HR-ToF-AMS) with a novel $PM_{2.5}$ lens at the Institute of Earth Environment, Chinese Academy of Sciences (34.23_ N, 108.88_ E) in Xi'an, China[1]. For the Shanghai and Hangzhou cases, daily chemical composition of $PM_{2.5}$ at Lian, Taiyangshan and Zhengzhou stations was obtained from the Chinese Meteorological Administration (CMA) Atmospheric Watch Network (CAWNET)[2]. Note that the observations of chemical composition of $PM_{2.5}$ at Zhengzhou and Gaolanshan stations from CAWNET are also used for the model evaluation for Beijing and Xian cases, respectively, as shown in Table S1b.

48-h air mass back trajectories and their cluster analyses. To locate possible regional transport pathways of air masses and evaluate the relative contributions by long range transport, 48-h back trajectories starting at the arrival level of 100 m from the monitoring sites were calculated with the NOAA HYSPLIT model (http://ready.arl.noaa.gov/HYSPLIT.php) for each period studied. The back trajectories were calculated eight times per day at starting times of 00:00, 03:00, 6:00, 09:00, 12:00, 15:00,18:00 and 21:00 UTC. To be consistent with the WRF-CMAQ model simulations, the same WRF meteorological fields are used to calculate the back trajectories. The trajectory cluster analysis for each case was performed with the clustering option of Euclidean distance[10,33,36]. Figs S2-S5 show the results of the trajectory cluster analyses and the 48 h back trajectories in different periods on the basis of observed $PM_{2.5}$ concentration intervals (6 intervals: the entire period,75 $\mu g\ m^{-3} \leqslant PM_{2.5} < 115\ \mu g\ m^{-3}$, 115 $\mu g\ m^{-3} \leqslant PM_{2.5} < 150\ \mu g\ m^{-3}$, 150 $\mu g\ m^{-3} \leqslant PM_{2.5} < 250\ \mu g\ m^{-3}$, $PM_{2.5} \geqslant 250\ \mu g\ m^{-3}$ and $PM_{2.5} \geqslant 150\ \mu g\ m^{-3}$) for the five heavy haze episodes in Beijing, Shanghai, Hangzhou and Xian. To discuss differences in the distributions of backward trajectory clusters arriving at the receptor sites in the vertical direction, the pressure profiles of the trajectory clusters for each case are also shown in Figs. S2a(g)-S5g. Table S8 summarizes the results of mean $PM_{2.5}$ concentrations, and percentages of trajectories for each trajectory cluster for five cases. The corresponding values of pressures and heights for each trajectory cluster at 48-h earlier before arriving at the receptor sites are also summarized in Table S8.

For the Beijing case in the forecast simulations from Jan 24-26, 2017, Fig. S2a shows that three clusters for all data during the entire period were determined by the cluster analysis algorithm: one short distance transport pathway: SW (Southwest), and two long distance transport pathways: NW (Northwest) and W (West). Figs. S2a(d), S2a(e) and S2a(f) indicate that most of the 48 h back trajectories for the heavy haze periods with the $PM_{2.5} \geqslant 150\ \mu g\ m^{-3}$, which mainly belong to SW clusters, originated from the southwest of Beijing and brought the dirty air masses to Beijing by passing through the industrialized cities such as Baoding, Langfang, and Shijiazhuang. The vertical distributions of the trajectory clusters in Fig S2a(g) and Table S8 reveal that the heights of SW and W clusters at 48-h earlier before arriving at Beijing were 642 (938 hPa pressure) and 2284 (764 hPa pressure) m, respectively, indicating that back trajectories for these two clusters traveled through the industrialized cities in the low boundary layer. On the other hand, for the Beijing case in the retrospective simulations from Oct 27-Nov 3, 2103, Fig. S2b shows that three clusters for all data during the entire period were determined by the cluster analysis algorithm: one short distance transport pathway: SW (Southwest), and two long distance transport pathways: NW (Northwest) and E-SW (East-Southwest). Figs. S2b(d), S2b(e) and S2b(f) indicate that most of the 48 h back trajectories for the heavy haze periods with the $PM_{2.5} \geqslant 150\ \mu g\ m^{-3}$, which mainly belong to E-SW and SW clusters, originated from the southwest of Beijing and brought the dirty air masses to Beijing by passing through the industrialized cities such as Baoding, Langfang, Shijiazhuang, Cangzhou, Tangshan and Tianjin. The vertical distributions of the trajectory clusters in Fig S2b(g) and Table S8 reveal that the heights of E-SW and SW clusters at 48-h earlier before arriving at Beijing were 450.0 (954.0 hPa pressure) and 24.5 (1002.7 hPa pressure) m, respectively, indicating that back trajectories for these two clusters traveled through the industrialized cities in the low boundary layer. The results in Figs. S2a and 1b indicate that the sources affecting the formation of heavy haze in Beijing are slightly different, depending on the meteorological conditions although the pollution sources in the southwest of Beijing are mainly responsible for both heavy haze events.

For the Shanghai case, Fig. S3a shows that three clusters for all the data during the entire period were determined by the cluster analysis algorithm: one long distance transport pathway: NW (Northwest), and two short distance transport pathways: NW-S (Northwest-South) and

NW-W (Northwest-West). Figs. S3d, S3e and S3f indicate that predominance of the 48 h back trajectories for the heavy haze periods with $PM_{2.5}$≥150 μg m^{-3}, which mainly belong to NW-S and NW-W clusters, originated from the northwest of Shanghai and passed through the industrialized cities such as Yangtze River Delta (Nanjing, Hehui, Hangzhou, Wuxi), Huaian, Suqian and Shijiazhuang. The vertical distribution of the trajectory clusters in Fig S3g and Table S8 reveals that the heights of NW-W and NW-S clusters at 48-h prior to arriving at Shanghai were 1651.7 (828.9 hPa pressure) and 1094.4 (884.7 hPa pressure) m, respectively.

For the Hangzhou case, Fig. S4a shows that three clusters for all data during the whole period were determined by the cluster analysis algorithm: NE (Northeast), NW (Northwest) and N (North). Figs. S4d, S4e and S4f indicate that most of the 48 h back trajectories for the heavy haze periods with the $PM_{2.5}$≥150 μg m^{-3} belong mainly to NW and N clusters, which came from the northwest and north of Hangzhou and passed through the industrialized cities such as Nanjing, Wuxi, Huaian, Rizhao and Jinan. The vertical distribution of the trajectory clusters in Fig S4g and Table S8 reveals that the heights of NW and N clusters at 48-h prior to arriving at Hangzhou were 1462.4 (847.5 hPa pressure) and 1224.3 (871.4 hPa pressure) m, respectively.

For the Xian case, Fig. S5a shows that four clusters for all data during the entire period were determined by the cluster analysis algorithm, two short distance transport pathways: SE (SouthEast) and E (East), and two slightly long distance transport pathways: NW (NorthWest) and N (North). Figs. S5d, S5e and S5f indicate that most of the 48 h back trajectories for the heavy haze periods with the $PM_{2.5}$≥150 μg m^{-3} came from all directions to Xian. The vertical distribution of the trajectory clusters in Fig S5g and Table S8 reveals that the heights of N, NW, SE and E clusters at 48-h prior to arriving at Xian were 1825.2 (812.2 hPa pressure), 2237.7 (774.0 hPa pressure), 360.5 (964.1 hPa pressure) and 690.7 (927.5 hPa pressure) m, respectively.

Evaluation of WRF-CMAQ model performance for the five severe haze episodes. In parallel with the hourly observations, concurrent hourly predicted concentrations at the monitoring sites in the city were averaged. The averaged observed and predicted concentrations are compared to evaluate the model performance for each haze episode, as

shown in Figs. S7-S10 for $PM_{2.5}$ at the related cities for each severe haze episodes. Fig. S6 shows the model simulations for $PM_{2.5}$ concentrations with observed data overlaid (circles) at 18:00 (local time), 20:00, 21:00 and 22:00 on December 2, 2013. As can be seen, the model captures the spatial pattern of most of observations reasonably well for this severe haze episode. Figs. S7-S10 show time-series comparisons of mean observed and predicted $PM_{2.5}$ concentrations for each city for the five severe urban haze cases. Time-series comparisons of observations and simulations for $PM_{2.5}$, PM_{10}, O_3, SO_2, NO_2, and CO in Beijing, Shanghai, Hangzhou and Xian are presented in Figs. S11, S12, S13 and S14a, respectively. Model performance in terms of normalized mean bias (NMB) values for $PM_{2.5}$, O_3, SO_2, NO_2, and CO for each city and each study case is summarized in Tables 2-5.

For the Beijing case in the forecast simulations from Jan 24-26, 2017, Fig. S7a shows that the model captured the temporal variations of mean $PM_{2.5}$ concentrations at all related cities very well. The NMB values for $PM_{2.5}$ range from -4.5% at Tangshan to -31.2% at Tianjin at all related cities (see Table S2a). The NMB values for SO_2 are within ±30% at all related cities except Beijing, Langfang and Tianjin where the NMB values for SO_2 are 54.7, 62.9 and 65.4%, respectively (see Table S2a). The NMB values for NO_2 are within ±20% at all related cities (see Table S2a). The NMB values for CO are within ±31% at all related cities except Qinhuangdao where the NMB value for CO is -36.2% (see Table S2a). On the other hand, for the Beijing case in the retrospective simulations from Oct 27-Nov 3, 2103, Fig. S7b shows that the model captured the temporal variations of mean $PM_{2.5}$ concentrations at all related cities very well except Tangshan city, for which there is consistent overestimation of $PM_{2.5}$. The NMB values for $PM_{2.5}$ range from 0.2% at Handan to -19.5% at Qinhuangdao at all related cities except Tangshan where the NMB value for $PM_{2.5}$ is 45.8% (see Table S2c). The NMB values for O_3 are within ±20% at all related cities except Langfang and Qinhuangdao for which the NMB values for O_3 are 42.3% and 42.9%, respectively (see Table S2c). The NMB values for SO_2 are within ±20% at all related cities except Hengshui and Qinhuangdao where the NMB values for SO_2 are 36.2% and 50.2%, respectively (see Table S2c). The NMB values for NO_2 are within ±30% at all related cities except Chengde and Qinhuangdao where the NMB values for NO_2 are -49.3% and -37.4%, respectively (see Table S2c).The NMB values for CO are within ±21% at all related cities except Qinhuangdao

where the NMB value for CO is -33.8% (see Table S2c).

For the Shanghai case, Fig. S8 shows that the model captured well the temporal variations of mean $PM_{2.5}$ concentrations at all related cities. The NMB values for $PM_{2.5}$ range from5.6% at Changzhou to -26.2% at Ningbo (see Table S3). The NMB values for O_3 are within ±40% at all related cities except Huzhou, Jiaxing and Ningbo where the NMB values for O_3 are 54.9%, 49.7% and 45.6%, respectively (see Table S3). The NMB values for SO_2 are within ±40% at all related cities except Nanjing and Suzhou where the NMB values for SO_2 are 51.5% and 56.0%, respectively (see Table S3). The NMB values for NO_2 are within ±33% at all related cities except Huzhou and Ningbo where the NMB values for $NO_2$are -41.8% and -38.8%, respectively (see Table S3).The NMB values for CO are within ±35% at all related cities except Nantong and Suzhou where the NMB values for CO are -47.3% and -47.5%, respectively (see Table S3).

For the Hangzhou case, Fig. S9 shows that the model captured the temporal variations of mean $PM_{2.5}$ concentrations at all related cities very well. The NMB values for $PM_{2.5}$are within ±25% except for Huaian, Huzhou, and Lianyungang where the NMB values for $PM_{2.5}$ are -35.9%, -37.1% and -45.8%, respectively (see Table S4).The NMB values for O_3 are within ±45% at all related cities (see Table S4). The NMB values for SO_2 are within ±35% except for Huaian, Lianyungang and Yangzhou where the NMB values for SO_2 are -48.7%, -48.6% and -46.1%, respectively (see Table S4). The NMB values for NO_2 are within ±35% except for Lianyungang where the NMB value for NO_2 are -44.0% (see Table S4).The NMB values for CO are within ±30% at all related cities except Changzhou and Huaian where the NMB values for CO are -38.4% and -55.5%, respectively (see Table S4).

For the Xian case, Fig. S10 shows that the model captured well the temporal variations of mean $PM_{2.5}$ concentrations at all related cities except for Tangchuan and Baoji where the model did not capture the peaks of observed $PM_{2.5}$ concentrations. The NMB values for $PM_{2.5}$range from -11.1% at Xian to -37.1% at Tongchuan (see Table S5). The NMB values for O_3 are within ±33% at all related cities except Baoji and Weinan where the NMB values for O_3 are 50.5% and 57.4%, respectively (see Table S5). The NMB values for SO_2 are within ±26% at all related cities except Xianyang and Yanan where the NMB values for SO_2 are 62.5% and -57.6%, respectively (see Table S5). The NMB values for NO_2 are within ±15%

at all related cities (see Table S5).The NMB values for CO are within ±34% at all related cities (see Table S5).

Model performances for $PM_{2.5}$ chemical composition on the basis of available measurements for the Beijing, Shanghai, Hangzhou and Xian cases in the retrospective simulations are summarized in Tables 6a, 6b, 6c and 6d, respectively. The temporal variations of comparisons of predictions and observations for each $PM_{2.5}$ component are shown in Figs. S15-S18. As can be seen, the model has reasonable performance for $PM_{2.5}$ chemical composition for different heavy haze episodes in different cases. For the Beijing case, the NMB values for EC, OC, NO_3^-, SO_4^{2-}, NH_4^+ at Beijing site (Zhengzhou site) are 10.3 (-3.54), -45.9 (-17.9), 18.8 (-28.6), -30.4 (-15.0) and -19.4% (-16.5%), respectively (see Table 6a). For the Shanghai case, the NMB values for EC, OC, NO_3^-, SO_4^{2-}, NH_4^+ at Lian site (Zhengzhou site) are 10.2 (30.6), -47.0 (-21.3), -21.7 (-36.3), 22.4 (-10.9) and 64.6 % (-21.3%), respectively, while the NMB values for EC, OC, NO_3^-, SO_4^{2-}, NH_4^+ at Taiyangshan are 85.6, 13.3, -10.9, 30.8 and 10.0 %, respectively, (see Table 6b). For the Hangzhou case, the NMB values for EC, OC, NO_3^-, SO_4^{2-}, NH_4^+ at Lian site (Zhengzhou site) are 35.4 (18.0), -38.2 (-36.8), -44.2 (-42.9), 97.6 (-26.4) and 39.4% (-41.2%), respectively, while the NMB values for EC, OC, NO_3^-, SO_4^{2-}, NH_4^+ at Taiyangshan are 113.3, -5.3, -32.5, 43.5 and 76.0%, respectively, (see Table 6c). For the Xian case, the NMB values for OC, NO_3^-, SO_4^{2-}, NH_4^+ at Xian site on the basis of daily (hourly) data are -32.5 (-35.9), 4.3 (3.5), -23.0 (-23.5) and -15.4% (-13.8%), respectively, while the NMB values for EC, OC, NO_3^-, SO_4^{2-}, NH_4^+ at Gaolanshan are 37.2, -32.1, -30.1, 34.0 and 38.1%, respectively (see Table 6d). In summary, the model simulations generally underestimate both SO_4^{2-} and NH_4^+ at urban sites (Beijing, Zhengzhou, and Xian sites) but overestimate both SO_4^{2-} and NH_4^+ at the rural sites (Linan, Taiyangshang and Gaolanshan) for all four heavy haze episodes, while the model simulations overestimate EC at all sites and cases except the Beijing case at Zhengzhou site where the model simulations slightly underestimate observed EC by -3.5% (see Table S6a). The model simulations underestimate OC at all sites and cases except the Shanghai case at Taiyangshan site where the model simulations slightly overestimate observed OC by 13.3% (see Table S6b), while the model simulations underestimate NO_3^- at all sites and cases except the Beijing case at the Beijing site and Xian case at Xian site where the model simulations overestimate

NO_3^- slightly. The uncertainties in emission inventories, the physical-chemical mechanisms of haze formation, and prognostic model simulation of meteorological fields cause the biases in the simulations of $PM_{2.5}$ chemical composition.

A detailed inspection of time-series results in Figs. 1, and S7-S14a shows that more variable features in the hourly model simulations relative to the observations are due to the fact that there is significant diurnal variability exhibited by the model but very little diurnal variation in the observations as shown in Fig. S14b for diurnal variations of mean observations and model simulations at Xian for the Xian case. The evaluation of several $PM_{2.5}$ forecast models by Mckeen et al.[3] also found the similar results for other models such as WRF-Chem and CHRONOS and CMAQ-ETA models. As pointed out by McKeen et al.[3], the diurnal variability in model simulations is due to the utilization of the PBL parameterization within the WRF formalism (The ACM2 PBL scheme is used in our WRF-CMAQ model case). The different PBL scheme will have different PBL growth that causes different timing of midmorning drawdown as analyzed by McKeen et al.[3]. Further investigation is needed for this in the future. On the other hand, the normalized mean bias (NMB) values for predictions of $PM_{2.5}$ at Beijing, Shanghai, Hangzhou and Xian are -2.8%, -14.5%, -11.4%% and -11.1%, respectively (Tables S2-S5). The results demonstrate skill in reproducing the urban $PM_{2.5}$, O_3, SO_2, NO_2 and CO concentrations for these haze episodes.

Effectiveness of the PAPCA. For the severe haze period from 10:00 on December 1 to 14:00 on December 3 in Shanghai, the mean $PM_{2.5}$ concentration decreased from 172.1μg m^{-3} (range 62.2 from to 272.9 μg m^{-3}) to 166.1 (58.9 to 259.0), 155.5 (55.8 to 238.3), 79.7 (39.5 to 113.2), and 64.9 (36.3 to 94.2) μg m^{-3} for the cases ECS1, ECS2, ECS3 and ECS 4, respectively (Fig. 3b). For the severe haze period from 9:00 on December 16 to 14:00 on December 25 in Xian, the mean $PM_{2.5}$ concentration decreased from 347.9μg m^{-3} (range from 150.8 to 595.3 μg m^{-3}) to 335.1 (144.5 to 565.0), 299.4 (128.8 to 475.1), 165.5 (54.8 to 295.5), and 58.4 (7.1 to 145.0) μg m^{-3} for the cases ECS1, ECS2, ECS3 and ECS 4, respectively (Fig. 3d).

Contributions of different emission sectors over the Beijing-Tianjin-Hebei region to $PM_{2.5}$ concentrations in Beijing. The sensitivity of $PM_{2.5}$ concentrations in Beijing to anthropogenic emissions over the Beijing-Tianjin-Hebei region were analyzed through the brute force method (BFM)[4]. Seven simulation scenarios for Beijing case for the period from Jan 22-26, 2017, were designed: Scenario Base (the base case) in which the emissions from all types of sources are included, and Scenarios All, Agr, Ind, Pow, Res and Tra in which the pollutant emissions of all (agriculture + industrials + power plants + residential + transportation), agriculture, industrials, power plants, residential, and transportation were zeroed out, respectively. The changes in simulated $PM_{2.5}$ ground concentrations at 12 sites (see Table S1a) in Scenarios All, Agr, Ind, Pow, Res and Tra compared to those in the base case are illustrated in Fig. S 21. Summarized in Table S9 are the contributions of all, agriculture, industrials, power plants, residential, and transportation sectors to mean $PM_{2.5}$ in Beijing. As can be seen, the mean contributions of the all, agriculture, industrials, power plants, residential, and transportation sectors to average $PM_{2.5}$ concentrations in Beijing were estimated at 91.5, 5.6, 28.71, 3.0, 40.9, and 5.5%, respectively. The results in Table S9 also show that the transport from outside of the Beijing-Tianjin-Hebei region contributed 8.5% to average $PM_{2.5}$ concentrations in Beijing.

Comparison of the results for the Beijing case in 2013 retrospective simulations and in 2017 forecast simulations. Table S9 shows that the mean reduction percentages of $PM_{2.5}$ for the Beijing case in 2013 retrospective simulations in Cases 1, 3, and 5 were 33.0%, 20.7% and 9.9%, respectively, where they for the Beijing case in 2017 forecast simulations are 32.6%, 20.4% and 9.8%, respectively. The results are very similar but with slightly broader source regions for the 2013 episode as shown in Figs. 2a and S1b. The significant differences in total emission control amounts between the 2013 and 2017 cases in Beijing as shown in Tables 2 and S7 is due to the fact that the heavy haze episode in 2013 last much longer and affected by the broader source regions relative to those in 2017 Beijing case.
The emission control times for the Beijing case in 2013 and 2017 are from 00:00 on January 22 to 24:00 on January 26, 2017 (total 120 hours) and from 00:00 on October 24 to 24:00 on November 3, 2013 (total 264 hours). The results for the Beijing case in 2013 retrospective

simulations and in 2017 forecast simulations indicate that the PAPCA works well for the same city but under different pollution episodes with the meteorological conditions that are totally different.

References

1. Li, G., *et al*. A possible pathway for rapid growth of sulfate during haze days in China. *Atmos. Chem. Phys.*, 17, 3301–3316 (2017)
2. Zhang, X.Y., *et al*. Atmospheric aerosol compositions in China: spatial/temporal variability, chemical signature, regional haze distribution and comparisons with global aerosols. *Atmos. Chem. Phys.*, **12**, 779–799 (2012)
3. McKeen, S. *et al.* The evaluation of several $PM_{2.5}$ forecast models using data collected during the ICARTT/NEAQS 2004 field study. *J. Geophys. Res.*, 112, D10S20, doi:10.1029/2006JD007608 (2007).
4. Dunker, A. M., *et al*. Photochemical modeling of the impact of fuels and vehicles on urban ozone using auto oil program data, *Environ. Sci. Technol.*, **30**, 787–801 (1996)

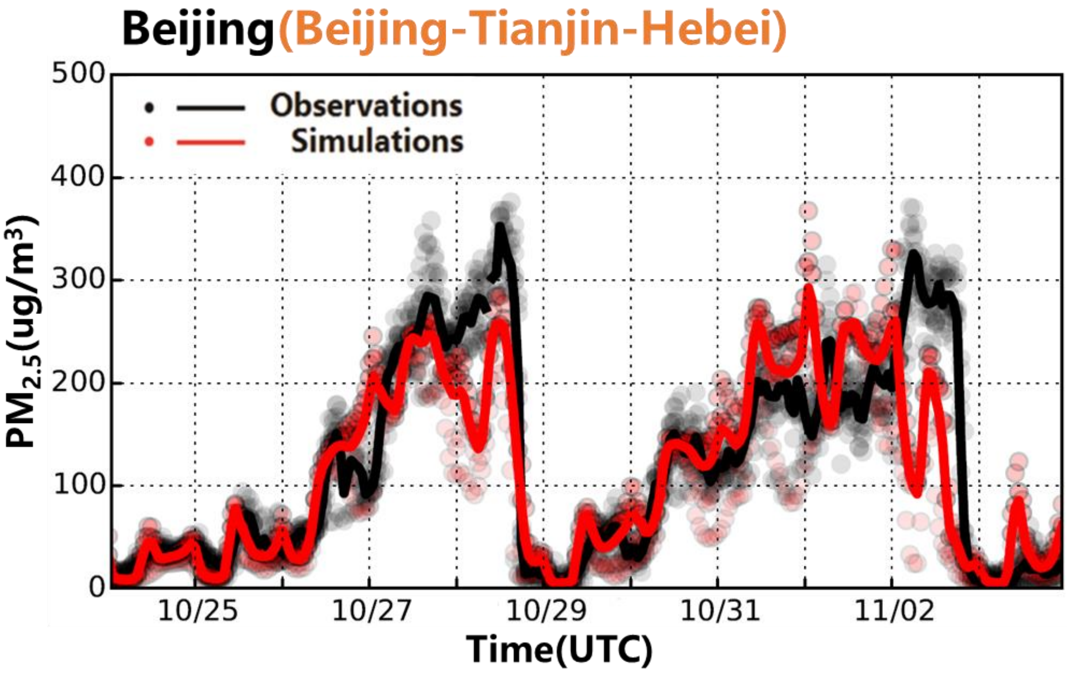

Fig. S1a. **Time-series comparisons of WRF-CMAQ model predictions and observations for $PM_{2.5}$ in Beijing from Oct 27 to Nov 3, 2013.**

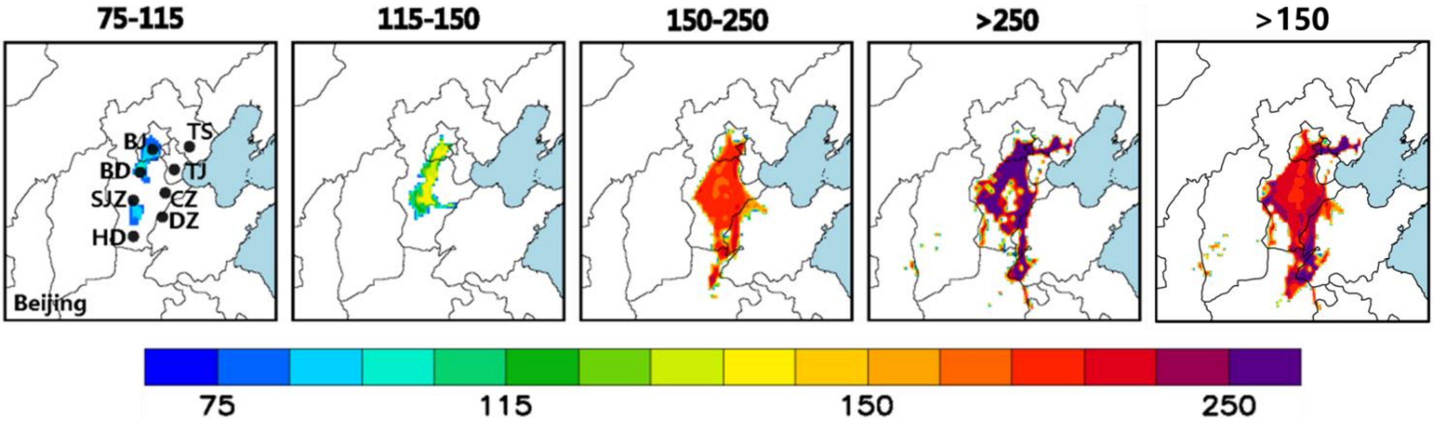

Fig. S1b. CWT values for $PM_{2.5}$ obtained from the hybrid receptor model to pinpoint origins of heavy haze pollution. (a) The spatial distributions of the four different CWT value intervals (75 μg m^{-3} ≤ CWT ≤ 115 μg m^{-3}, 115 μg m^{-3} ≤ CWT ≤ 150 μg m^{-3}, 150 μg m^{-3} ≤ CWT ≤ 250 μg m^{-3}, CWT ≥ 250 μg m^{-3}) for $PM_{2.5}$ in Beijing for the period from Dec 27 to Nov 3, 2013.

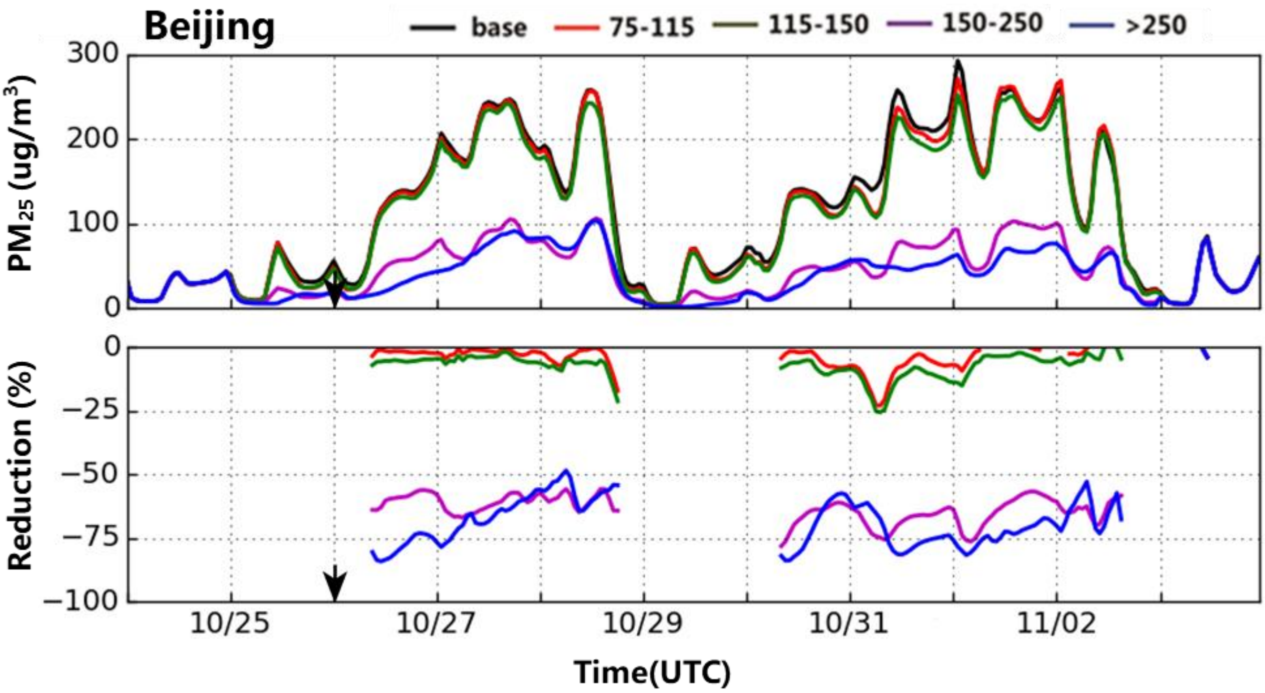

Fig. S1c. Test of effectiveness of the PAPCA strategy for the four different emission control scenarios. (a) Temporal variations of $PM_{2.5}$ concentrations and their reduction relative to the base case for the four different emission control scenarios on the basis of the four different CWT value intervals in Beijing for the period from Oct 27 to Nov 3, 2013.The proportional reduction is given only when the observations exceed 75μgm^{-3}.

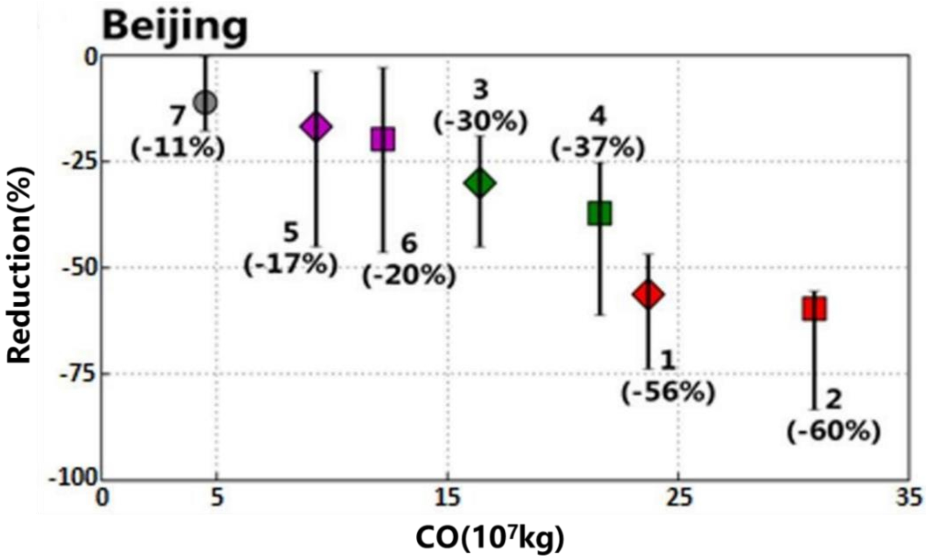

Fig. S1d. $PM_{2.5}$ reduction percentages as a function of the emission control amounts for the test of economic efficiency of the PAPCA. (a) The mean $PM_{2.5}$ reduction as a function of the CO emission control amounts for the 6 different cases in Beijing. Numbers 1-7 referred to the corresponding cases in Fig. S15 and Table 2. The same colors represent the pair comparisons like that cases 1 and 2 are the pair. The ranges of the reduction percentages are calculated on the basis of the hourly results for the studying periods. Here we use CO emission control amounts as the x-axis to represent the general emission control amounts because CO is a long-lived tracer of human activity associated with sources such as combustion, industry, mobile, and oxidation of hydrocarbons.

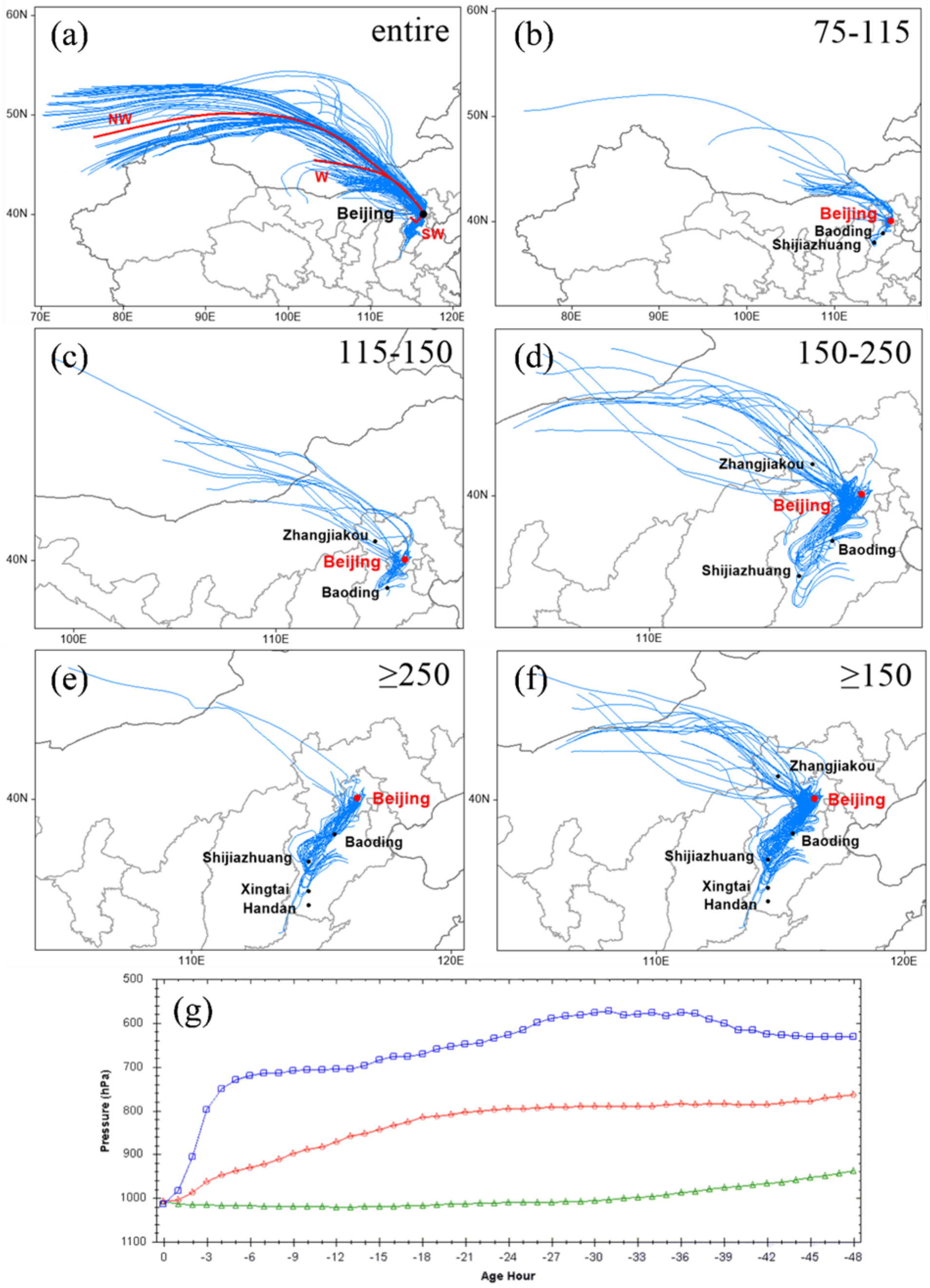

Fig. S2a. Cluster analysis of the 48-h air mass back trajectories starting at 100 m from the 10 monitoring sites in Beijing for the Beijing case for the period of Jan 24 to Jan 26, 2017. (a) All back trajectories for the entire dataset. Three transport pathways (clusters) are determined: NW (NorthWest), W (West) and SW (SouthWest); All back trajectories for the period with of (b) 75 μg m^{-3}≤$PM_{2.5}$<115 μg m^{-3}, (c) 115 μg m^{-3}≤$PM_{2.5}$<150 μg m^{-3}, (d) 150 μg m^{-3}≤$PM_{2.5}$<250 μg m^{-3}, (e) 250 μg m^{-3}≤$PM_{2.5}$ and (f) 150 μg m^{-3}≤$PM_{2.5}$; (g) Pressure profiles of three clusters.

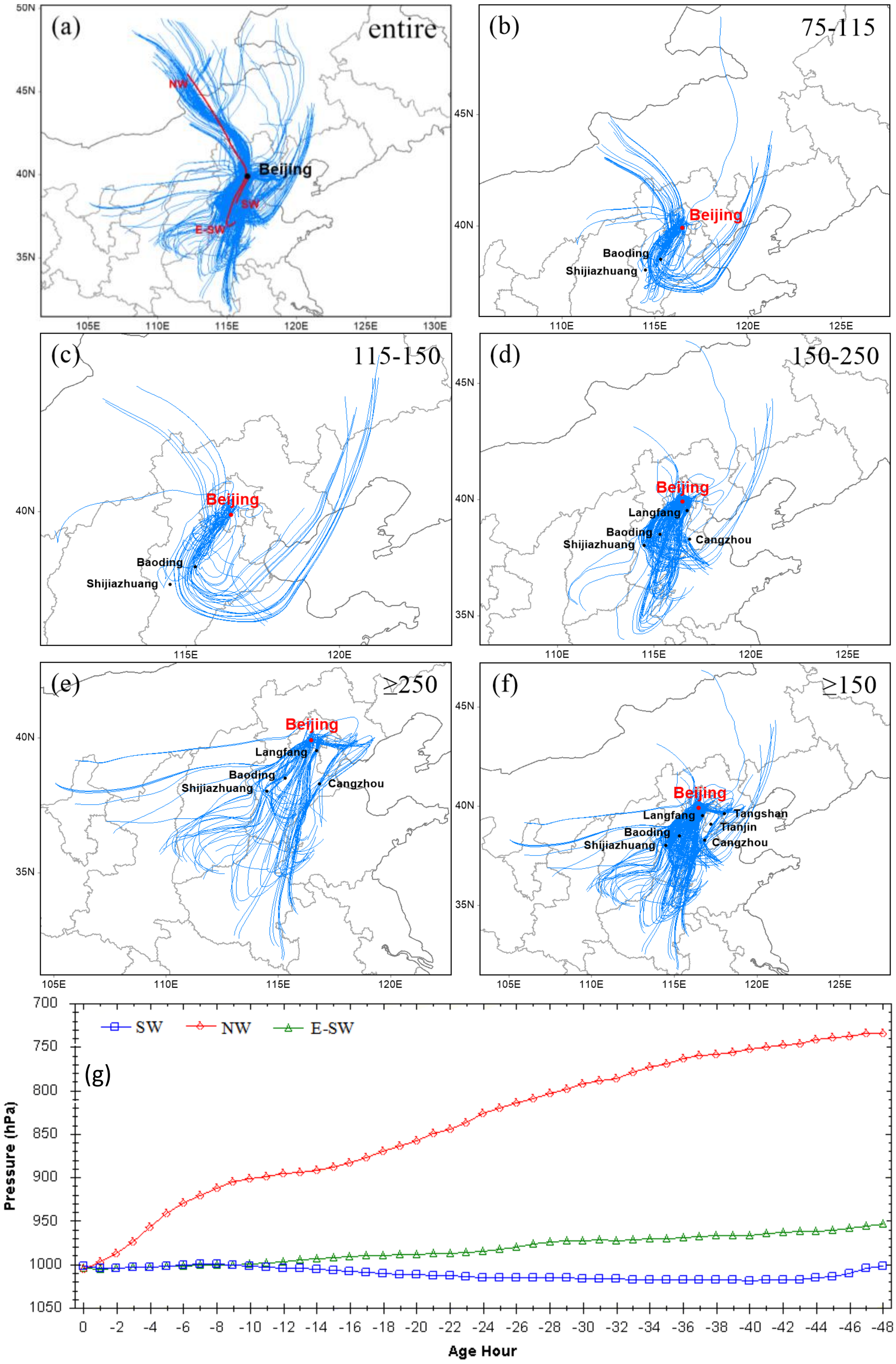

Fig. S2b. Cluster analysis of the 48-h air mass back trajectories starting at 100 m from the 10 monitoring sites in Beijing for the Beijing case for the period of Oct 27 to Nov 3, 2013. (a) All back trajectories for the entire dataset. Three transport pathways (clusters) are determined: SW (SouthWest), E-SW (East-SouthWest), and NW (NorthWest) ; All back trajectories for the period with of (b) 75 μg m^{-3}≤$PM_{2.5}$<115 μg m^{-3}, (c) 115 μg m^{-3}≤$PM_{2.5}$<150 μg m^{-3}, (d) 150 μg m^{-3}≤$PM_{2.5}$<250 μg m^{-3}, (e) 250 μg m^{-3}≤$PM_{2.5}$ and(f) 150 μg m^{-3}≤$PM_{2.5}$; (g) Pressure profiles of three clusters.

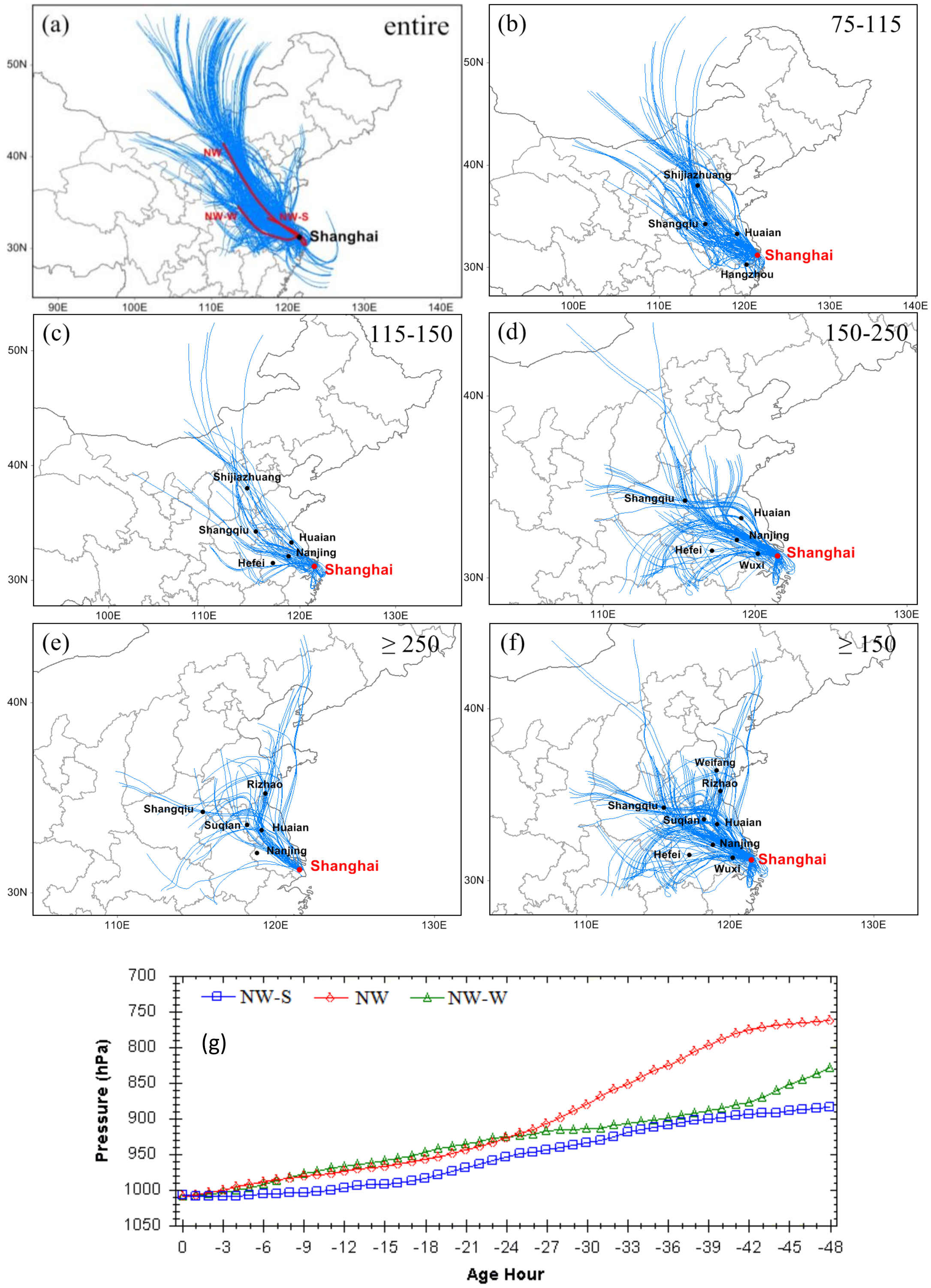

Fig. S3. The same as Fig. S2b but at Shanghai for the Shanghai case for the period of Nov 24 to Dec 4, 2013. Three transport pathways (clusters) are determined: NW-S (NouthWest-South), NW (NorthWest) and NW-W (NorthWest -West).

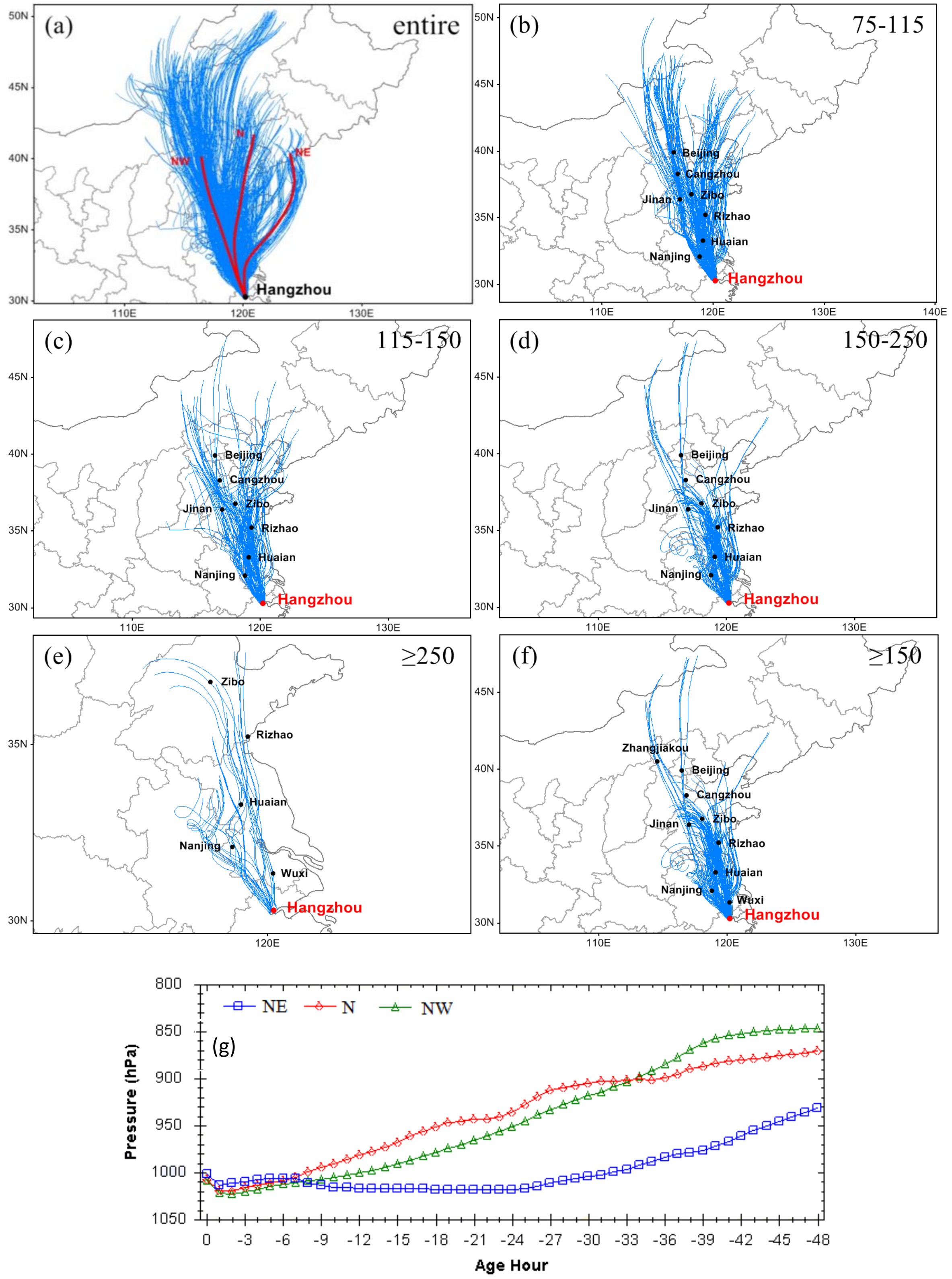

Fig. S4 The same as Fig. S2b but at Hangzhou for the Hangzhou case for the period of Dec 15 to Dec 28, 2013. Three transport pathways (clusters) are determined: NE (NouthEast), NW (NorthWest) and N (North).

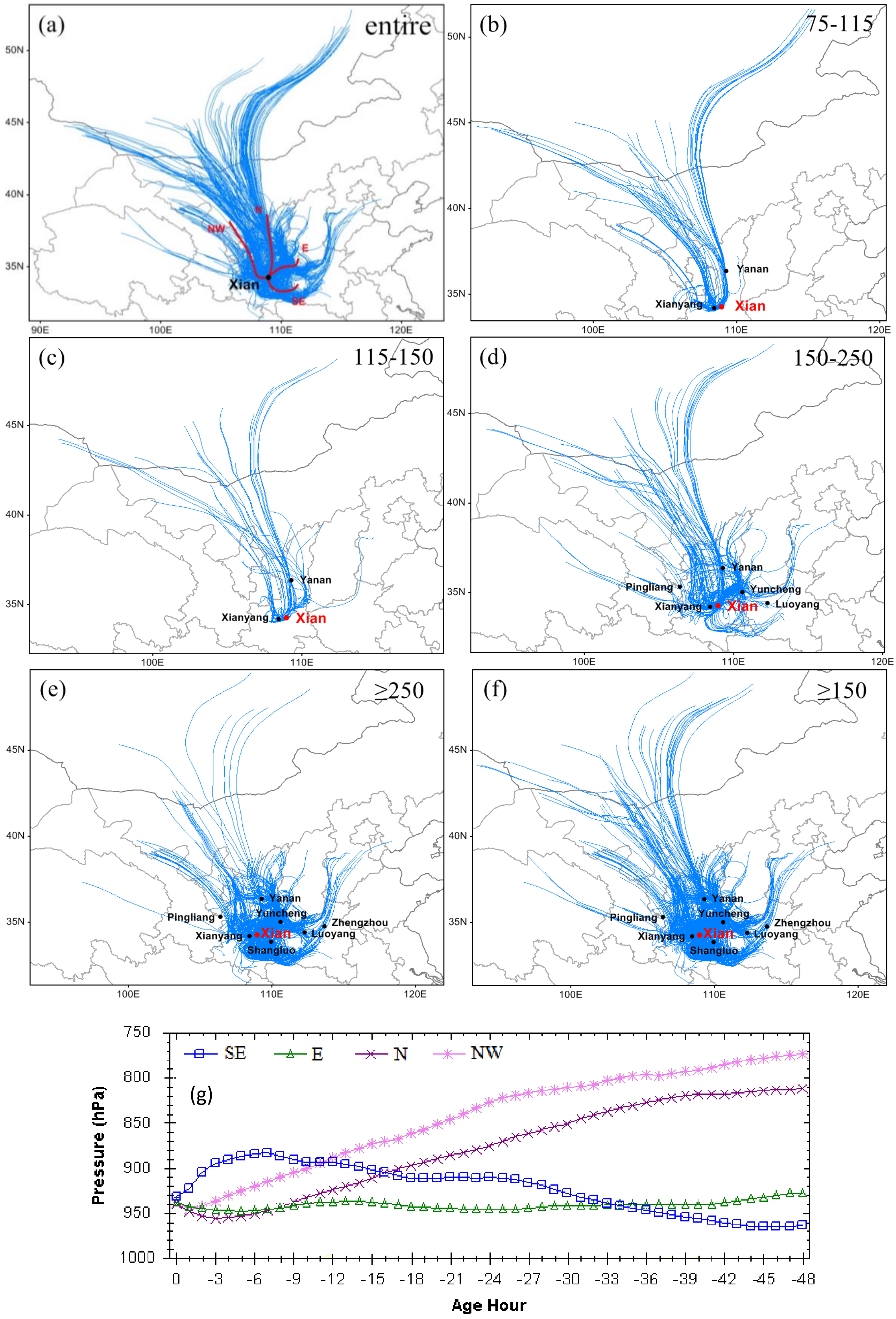

Fig. S5. The same as Fig. S2b but at Xian for the Xian case for the period of Dec 15 to Dec 28, 2013. Four transport pathways (clusters) are determined: SE (SouthEast), E (East), NW (NorthWest) and N (North).

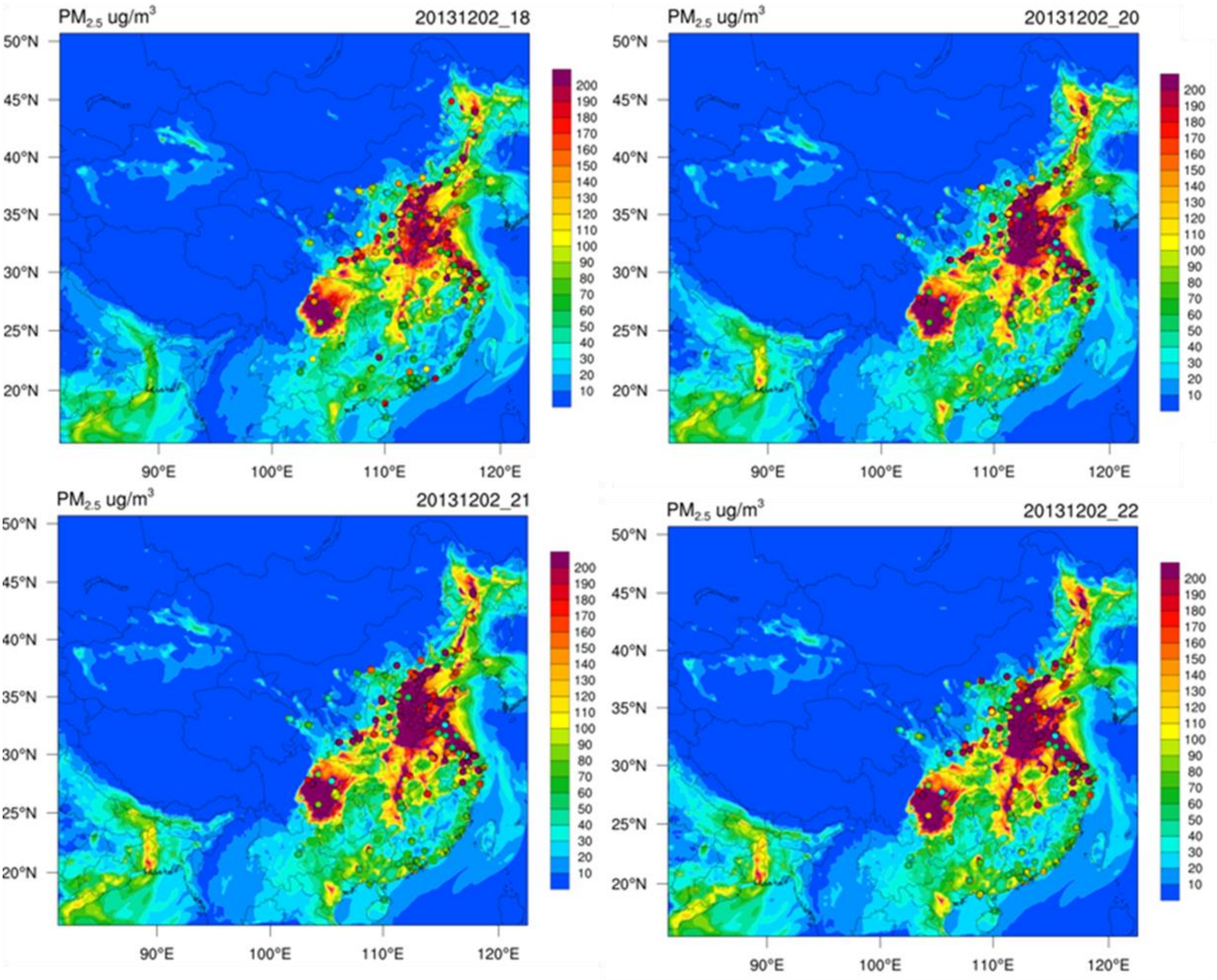

Fig. S6 Concentrations of $PM_{2.5}$ (particles with aerodynamic diameter lower than 2.5 μm) simulated by the WRF–CMAQ with observed data overlaid (circles) at 18:00 (local time), 20:00, 21:00, 22:00, December 2, 2013. The essential consistency between the model predictions and observations indicates that the spatial patterns of observed $PM_{2.5}$ concentrations are captured reasonably well

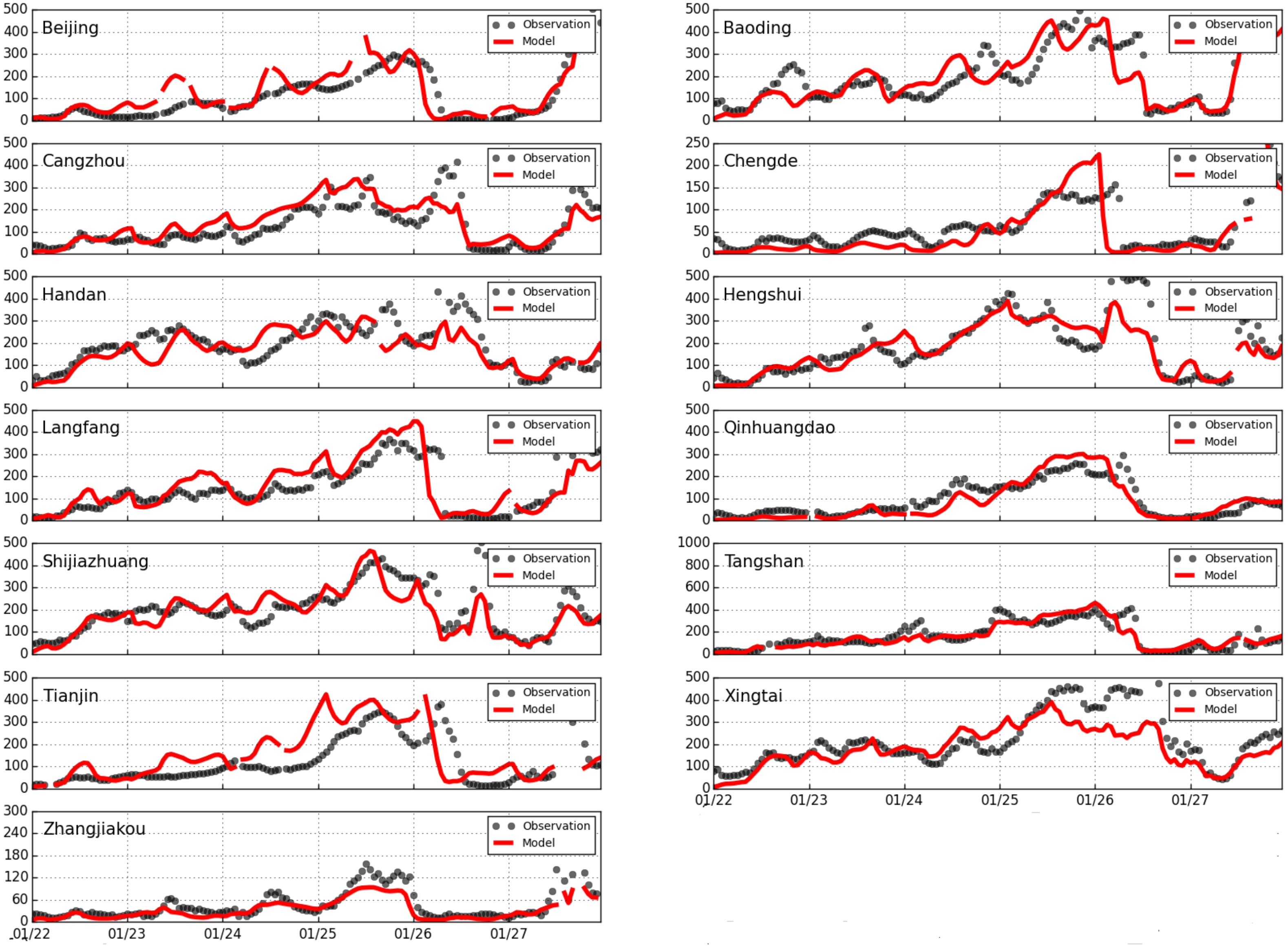

Fig. S7a Time-series comparisons of observations and model simulations for hourly mean $PM_{2.5}$concentrations at Beijing and its surrounding 12 cities for the Beijing forecast case (2017)on the basis of the data at the corresponding monitoring stations in each city.

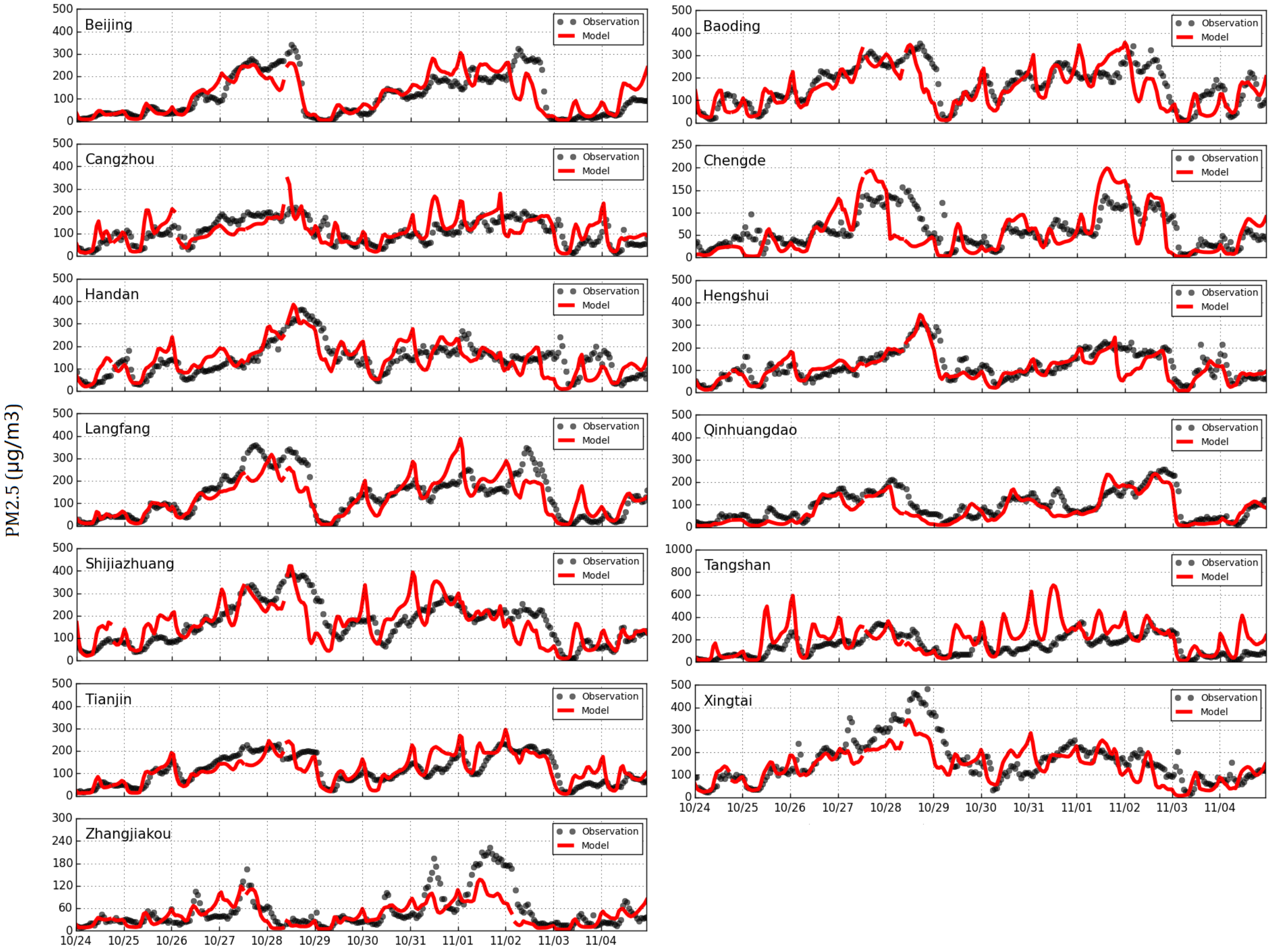

Fig. S7b Time-series comparisons of observations and model simulations for hourly mean $PM_{2.5}$concentrations at Beijing and its surrounding 12 cities for the Beijing case (2013)on the basis of the data at the corresponding monitoring stations in each city.

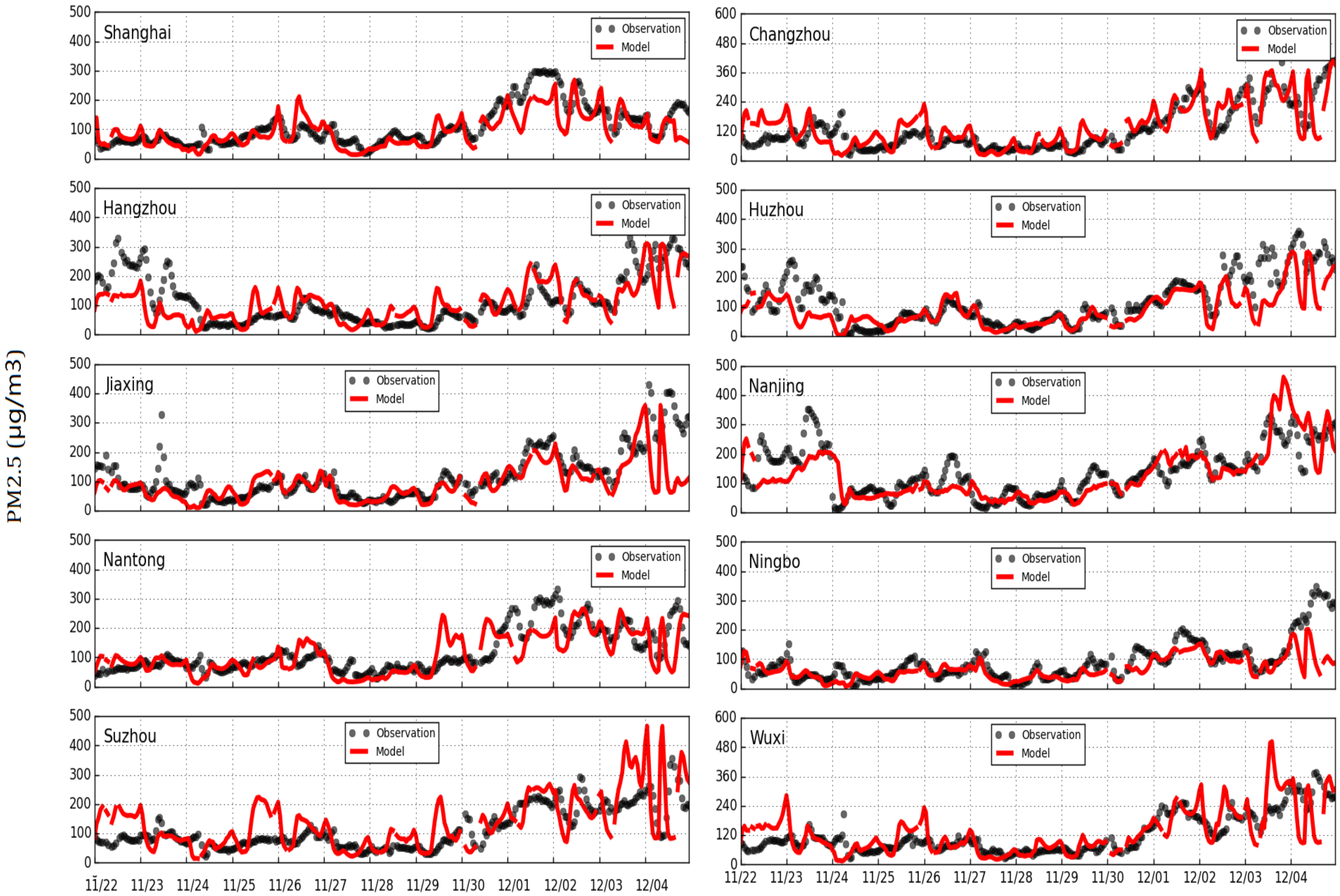

Fig. S8 Time-series comparisons of observations and model simulations for hourly mean $PM_{2.5}$concentrations at Shanghai and its surrounding 9 cities for the Shanghai case on the basis of the data at the corresponding monitoring stations on the basis of the data at the corresponding monitoring stations in each city.

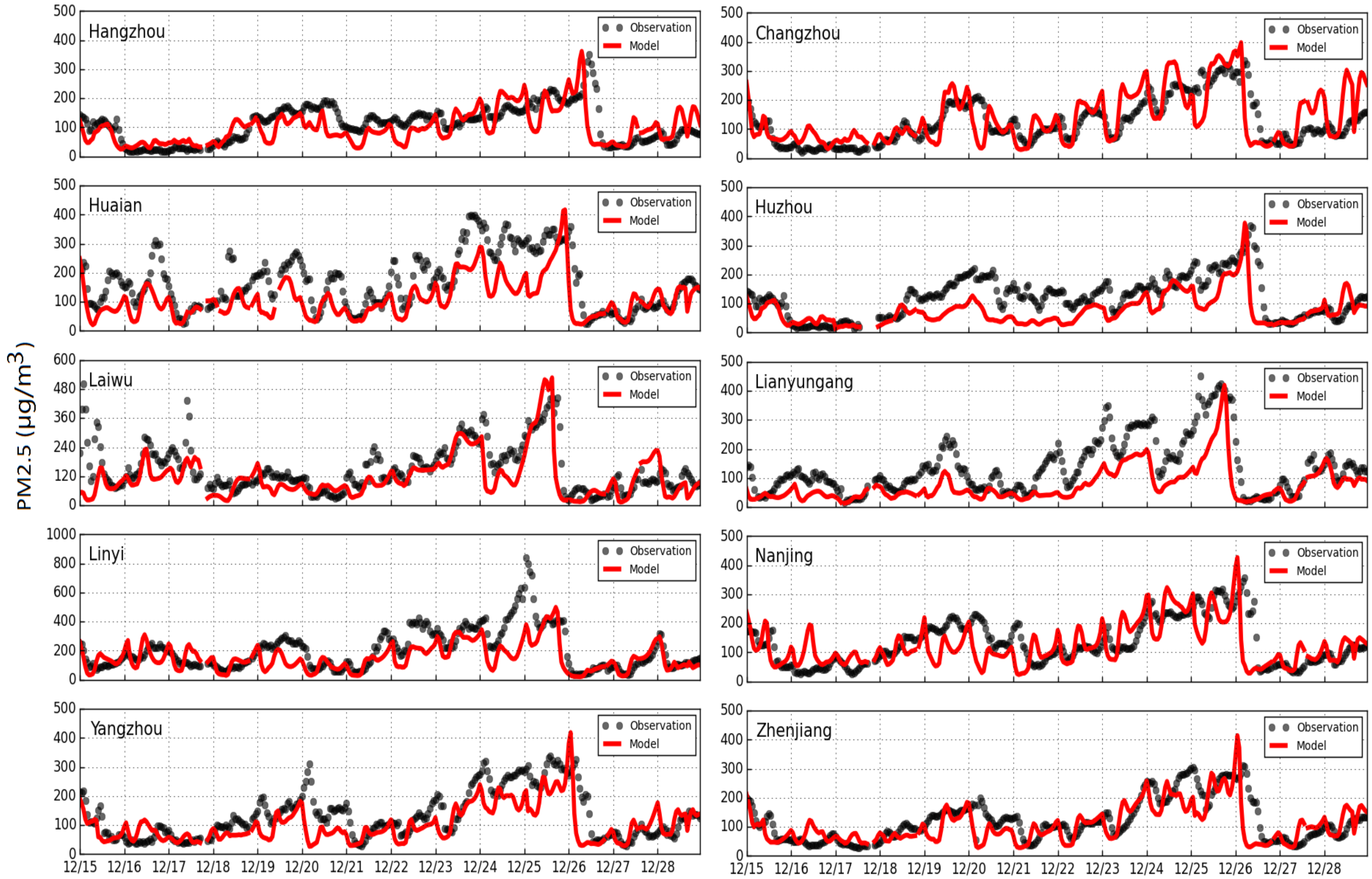

Fig. S9 Time-series comparisons of observations and model simulations for hourly mean $PM_{2.5}$concentrations at Hangzhou and its surrounding 9 cities for the Hangzhou case on the basis of the data at the corresponding monitoring stations in each city.

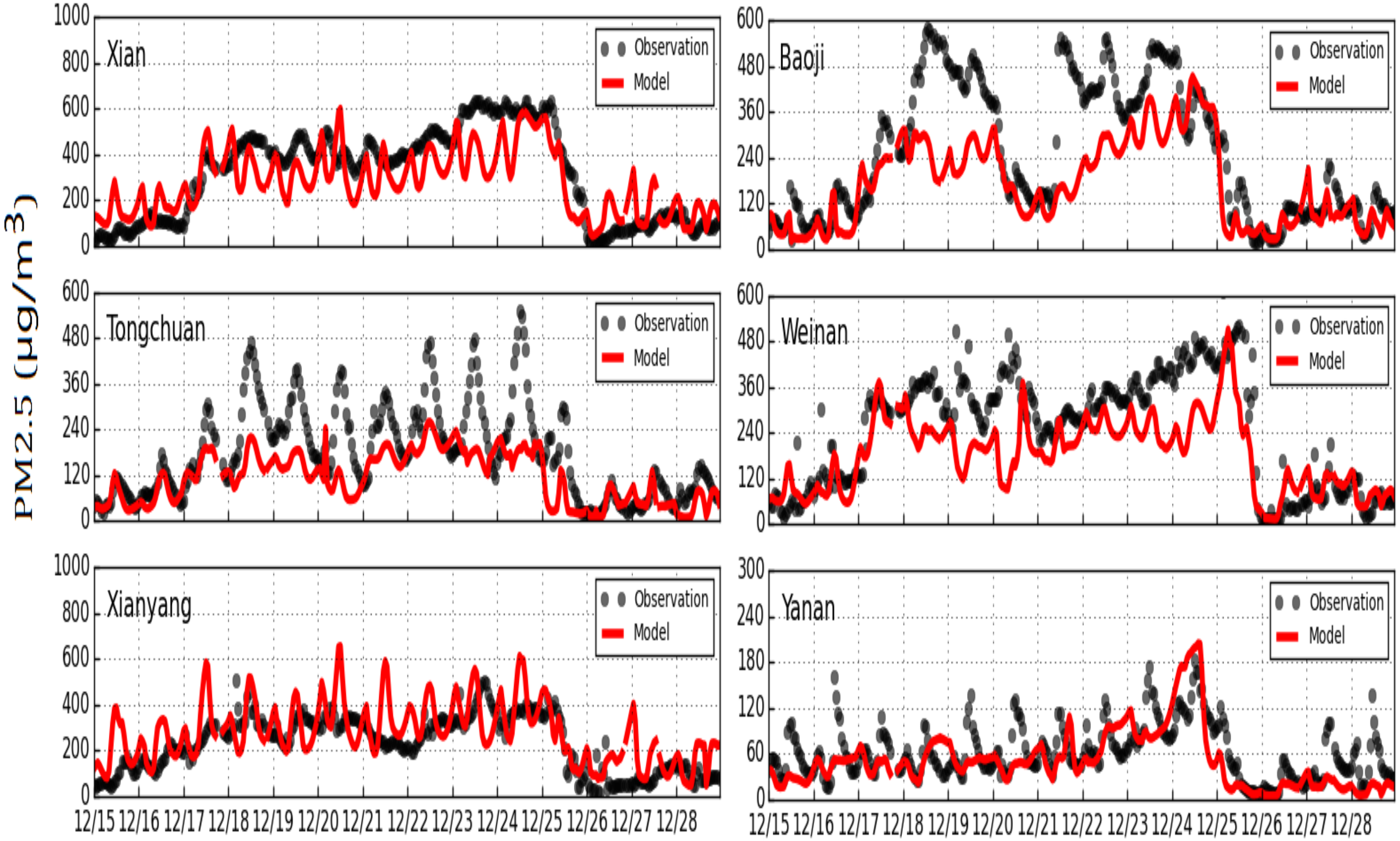

Fig. S10 Time-series comparisons of observations and model simulations for hourly mean $PM_{2.5}$concentrations at Xian and its surrounding 5 cities for the Xian case on the basis of the data at the corresponding monitoring stations in each city

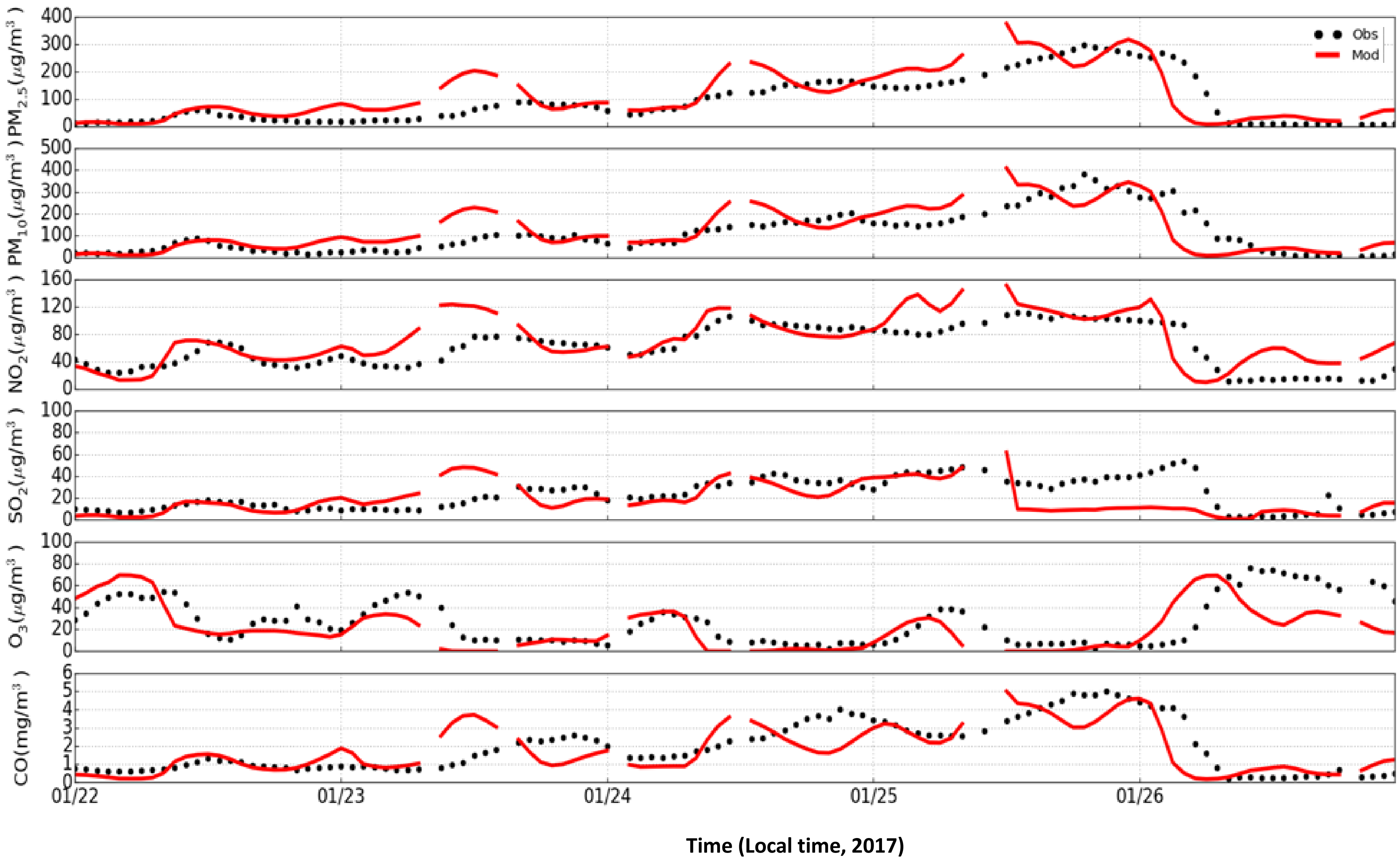

Fig. S11a Time-series comparisons of observations and model simulations for $PM_{2.5}$, PM_{10}, NO_2, SO_2, O_3, and CO hourly mean concentrations at Beijing for the Beijing forecast case from Jan 24-26, 2017.

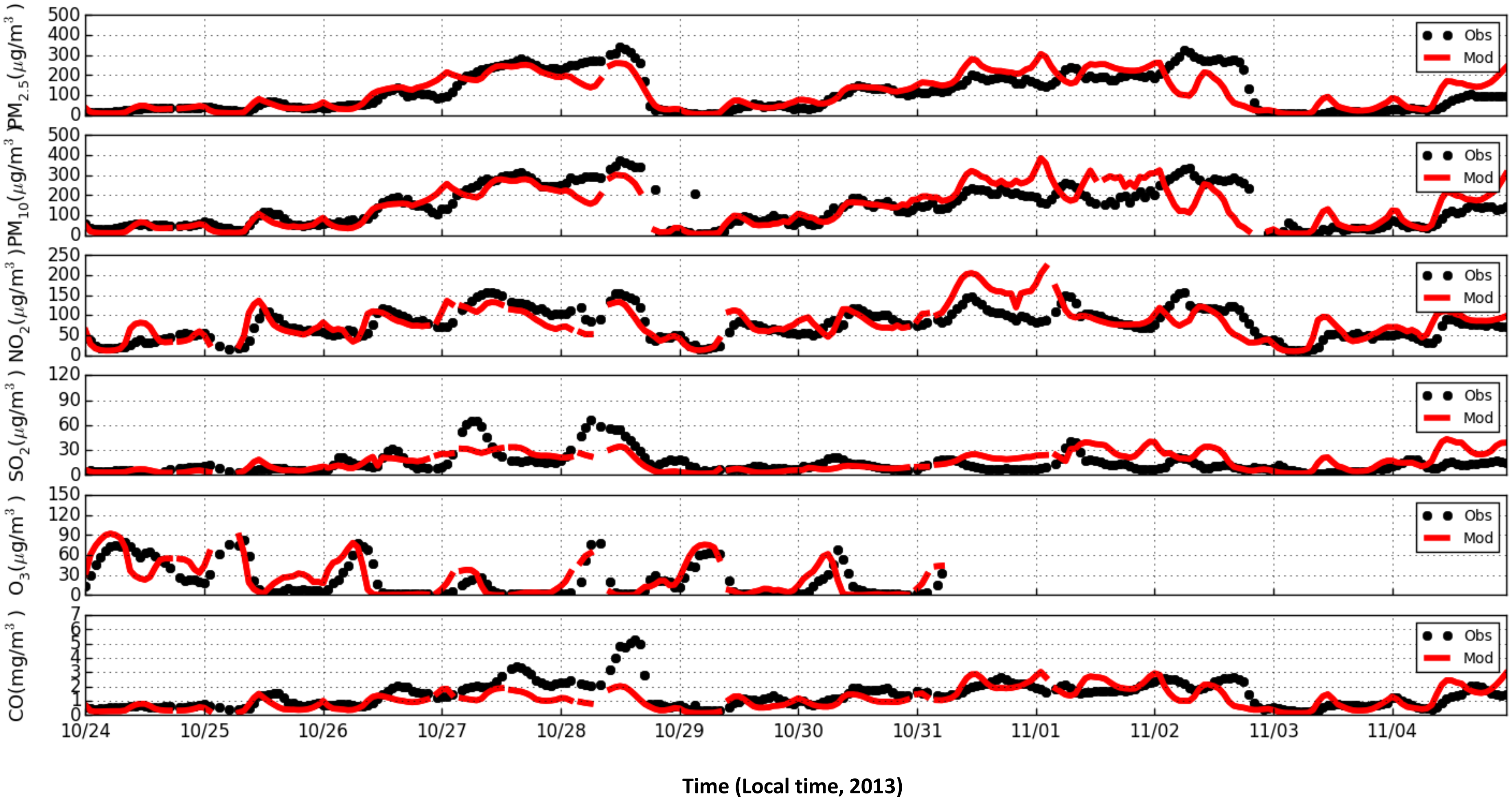

Fig. S11b Time-series comparisons of observations and model simulations for $PM_{2.5}$, PM_{10}, NO_2, SO_2, O_3, and CO hourly mean concentrations at Beijing for the Beijing case (2013)

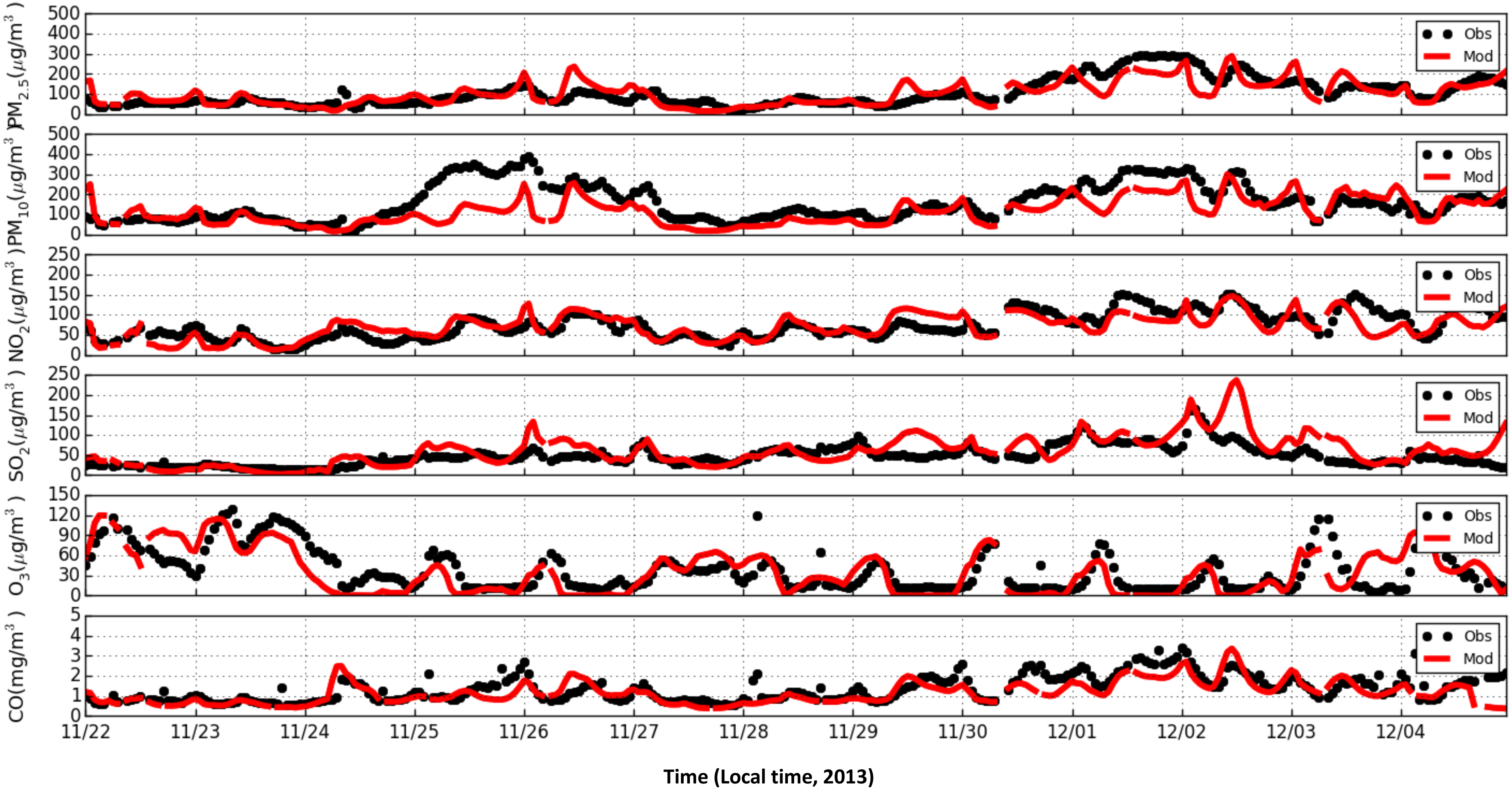

Fig. S12. Time-series comparisons of observations and model simulations for $PM_{2.5}$, PM_{10}, NO_2, SO_2, O_3 and CO hourly mean concentrations at Shanghai for the Shanghai case.

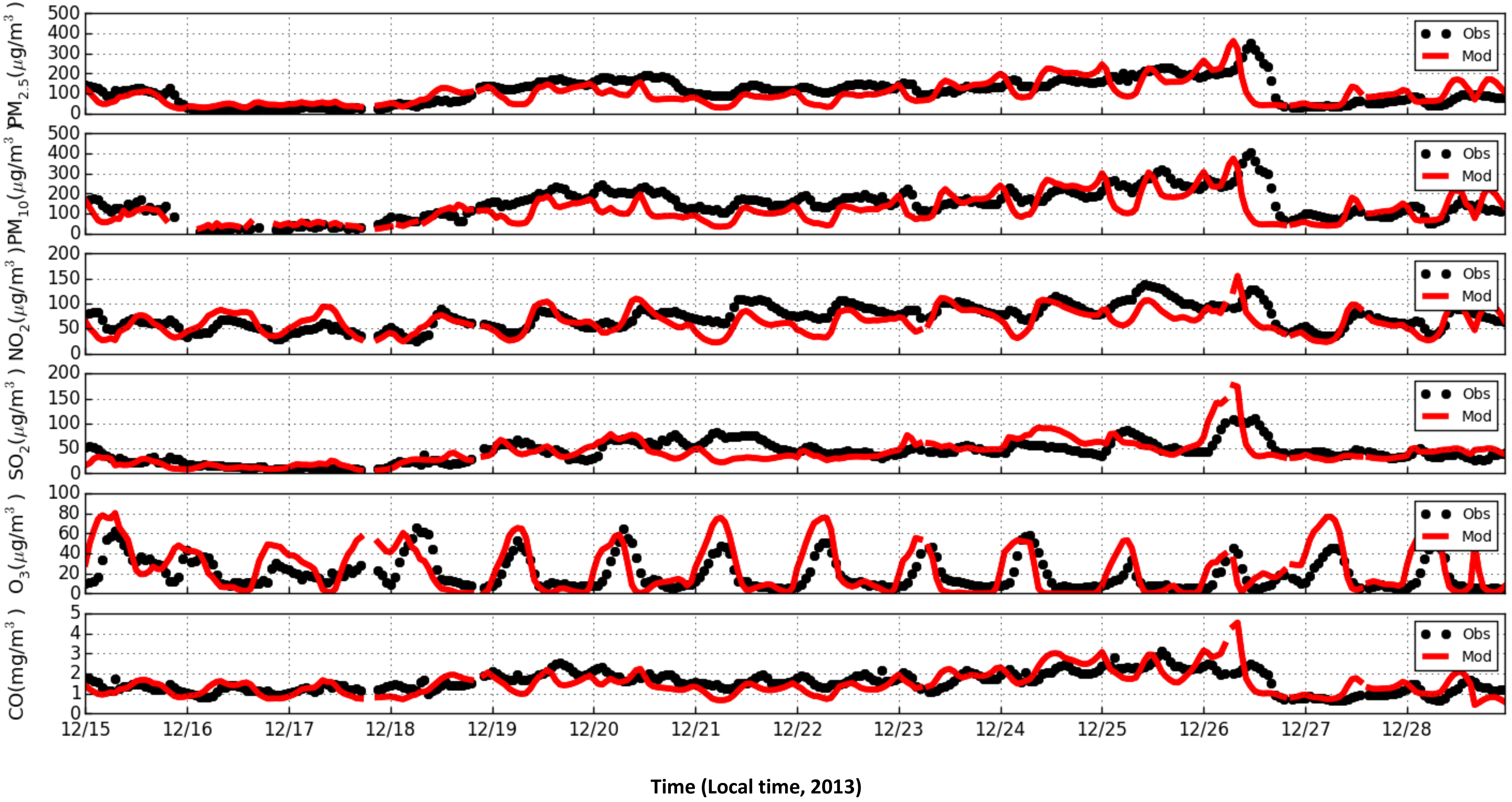

Fig. S13. Time-series comparisons of observations and model simulations for $PM_{2.5}$, PM_{10}, NO_2, SO_2, O_3 and CO hourly mean concentrations at Hangzhou for the Hangzhou case.

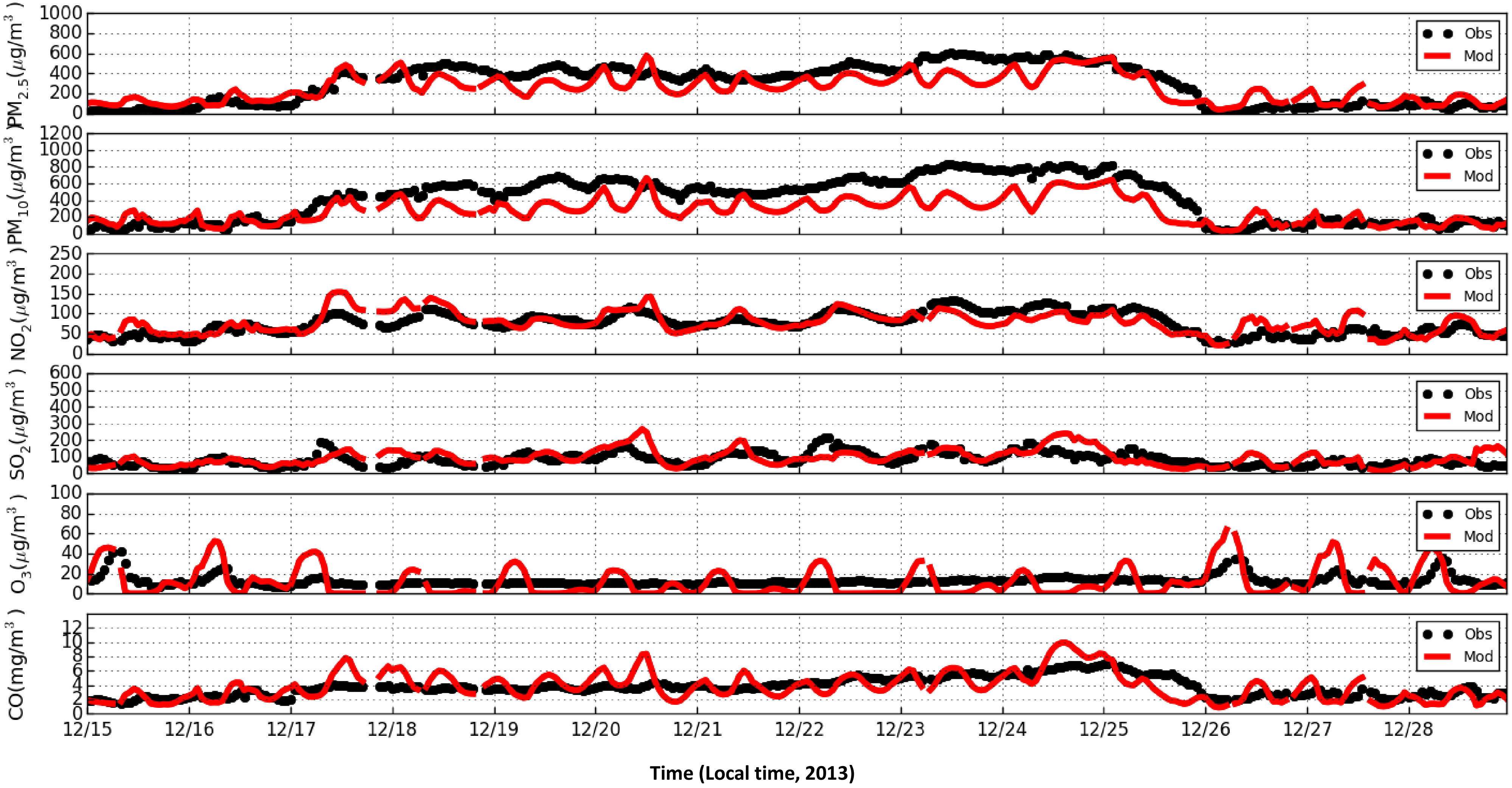

Fig. S14a. Time-series comparisons of observations and model simulations for $PM_{2.5}$, PM_{10}, NO_2, SO_2, O_3 and CO hourly mean concentrations at Xian for the Xian case.

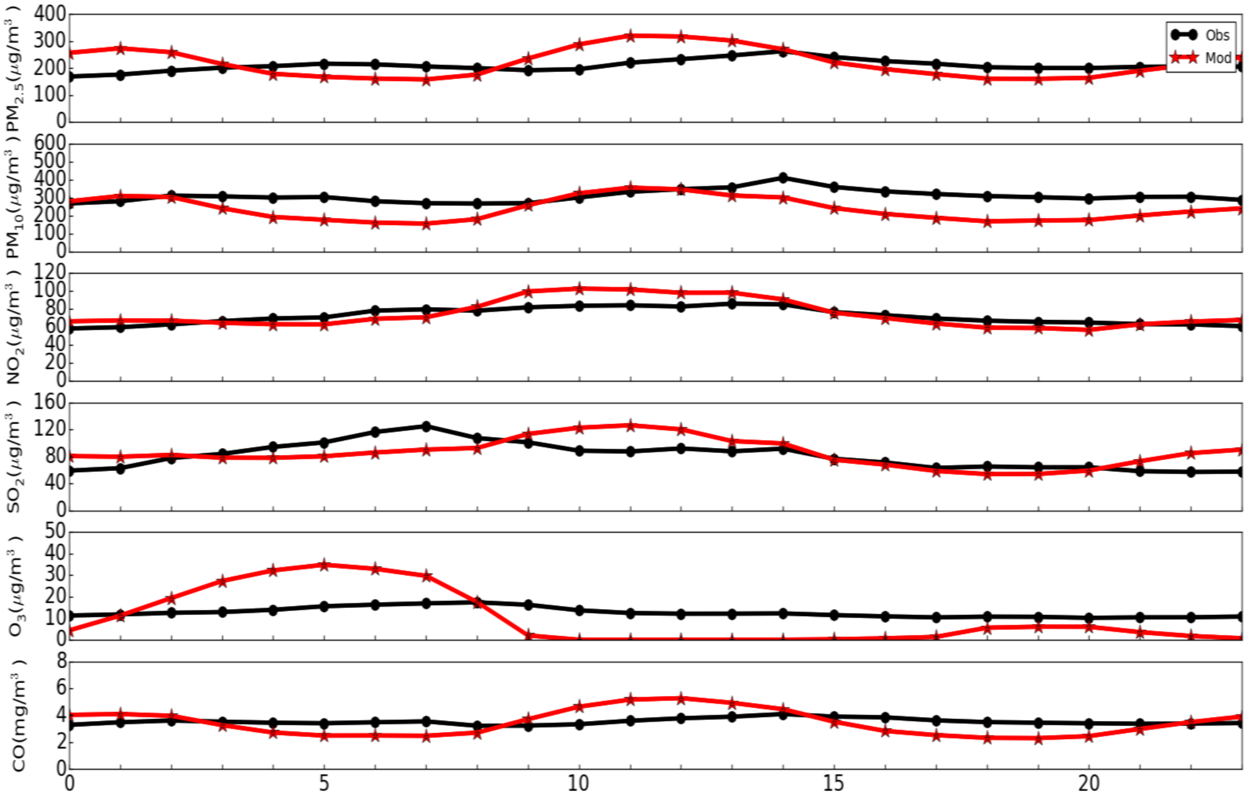

Time (local time)

Fig. S14b. Diurnal variations of mean observations and model simulations for $PM_{2.5}$, PM_{10}, NO_2, SO_2, O_3 and CO concentrations at Xian for the Xian case.

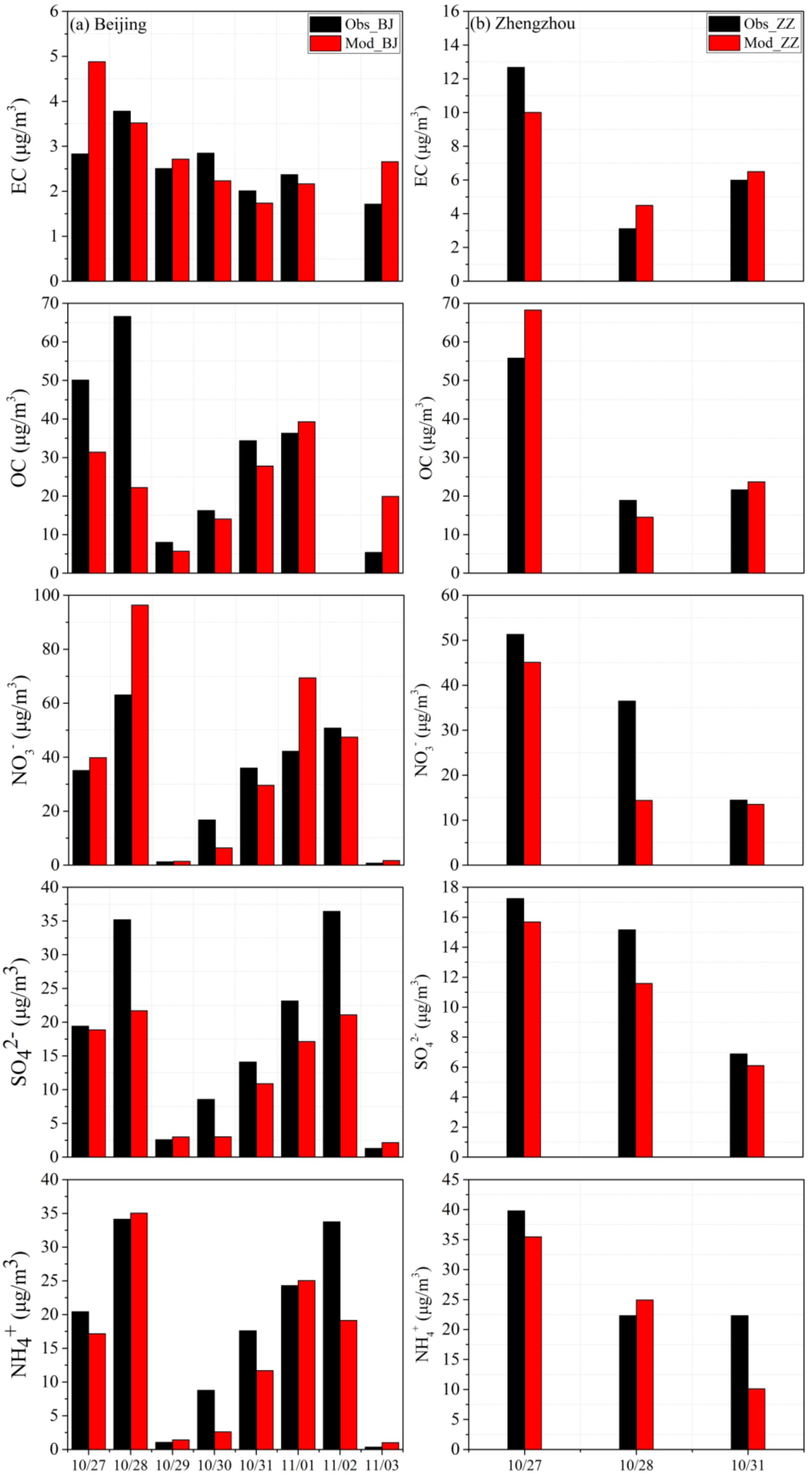

Fig. S15. Evaluation of CMAQ model performance for $PM_{2.5}$ chemical composition at Beijing and Zhengzhou for Beijing case.

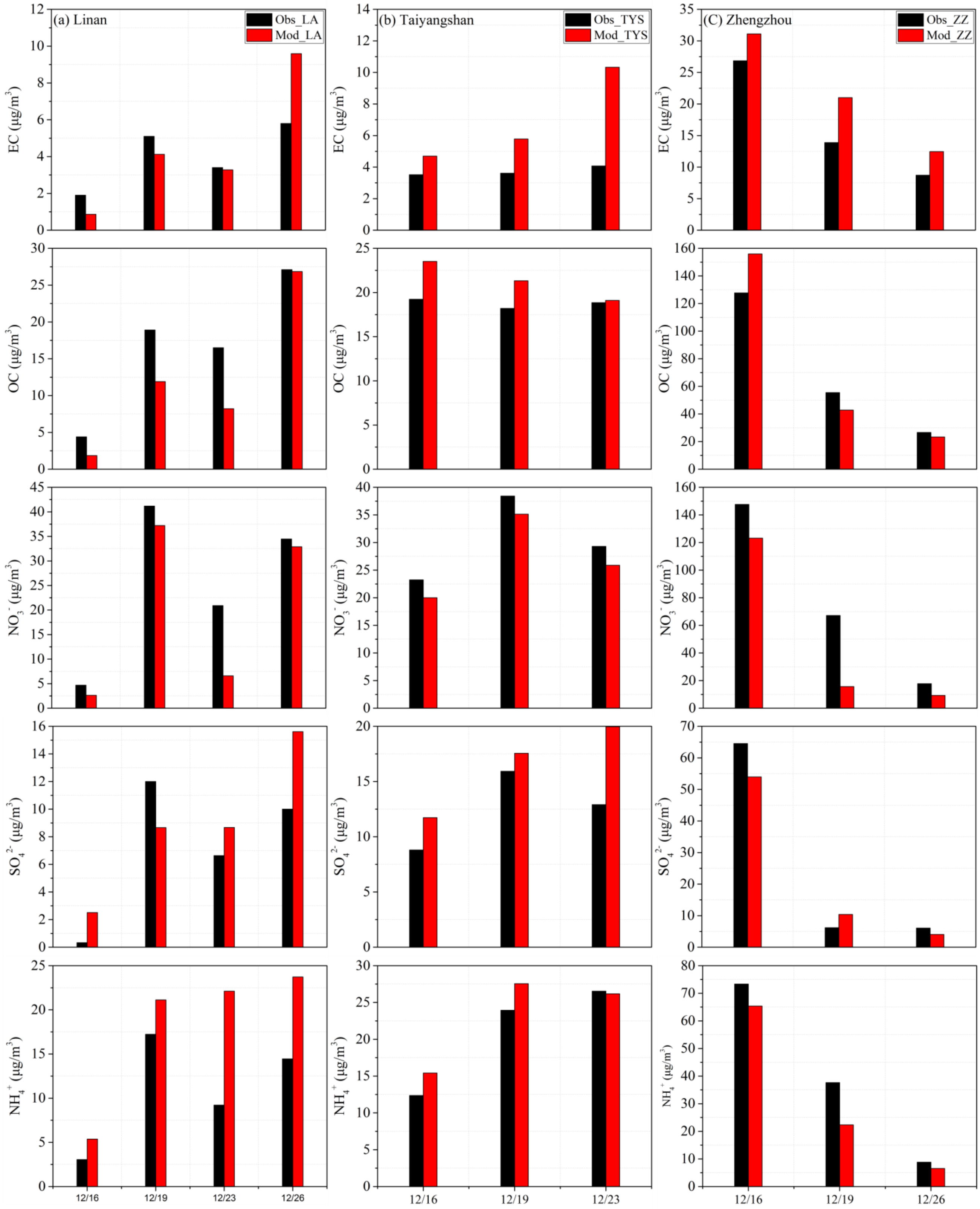

Fig. 16. Evaluation of CMAQ model performance for $PM_{2.5}$ chemical composition at Linan, Taiyangshan and Zhengzhou for Shanghai case.

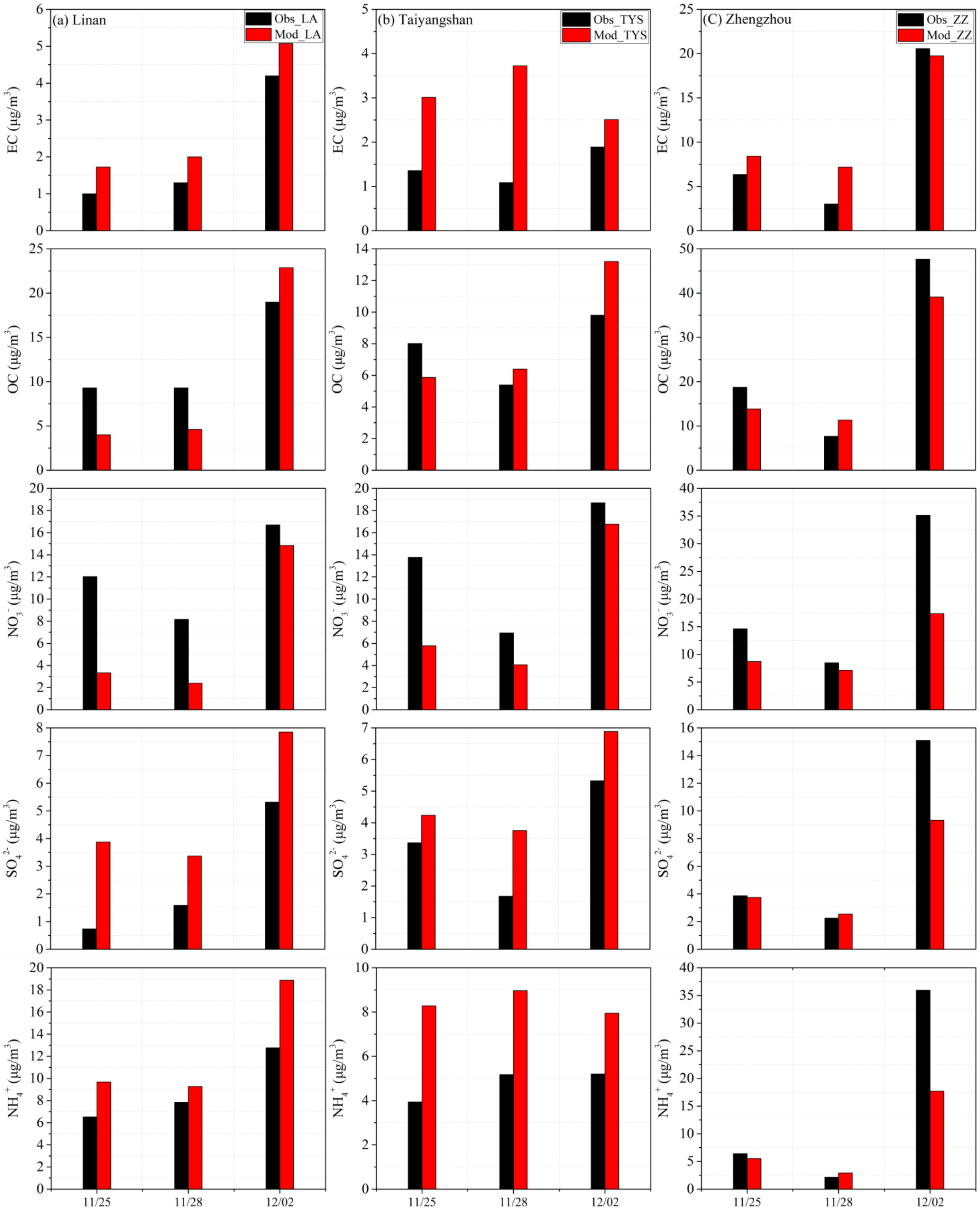

Fig. 17. Evaluation of CMAQ model performance for $PM_{2.5}$ chemical composition at Linan, Taiyangshan and Zhengzhou for Hangzhou case.

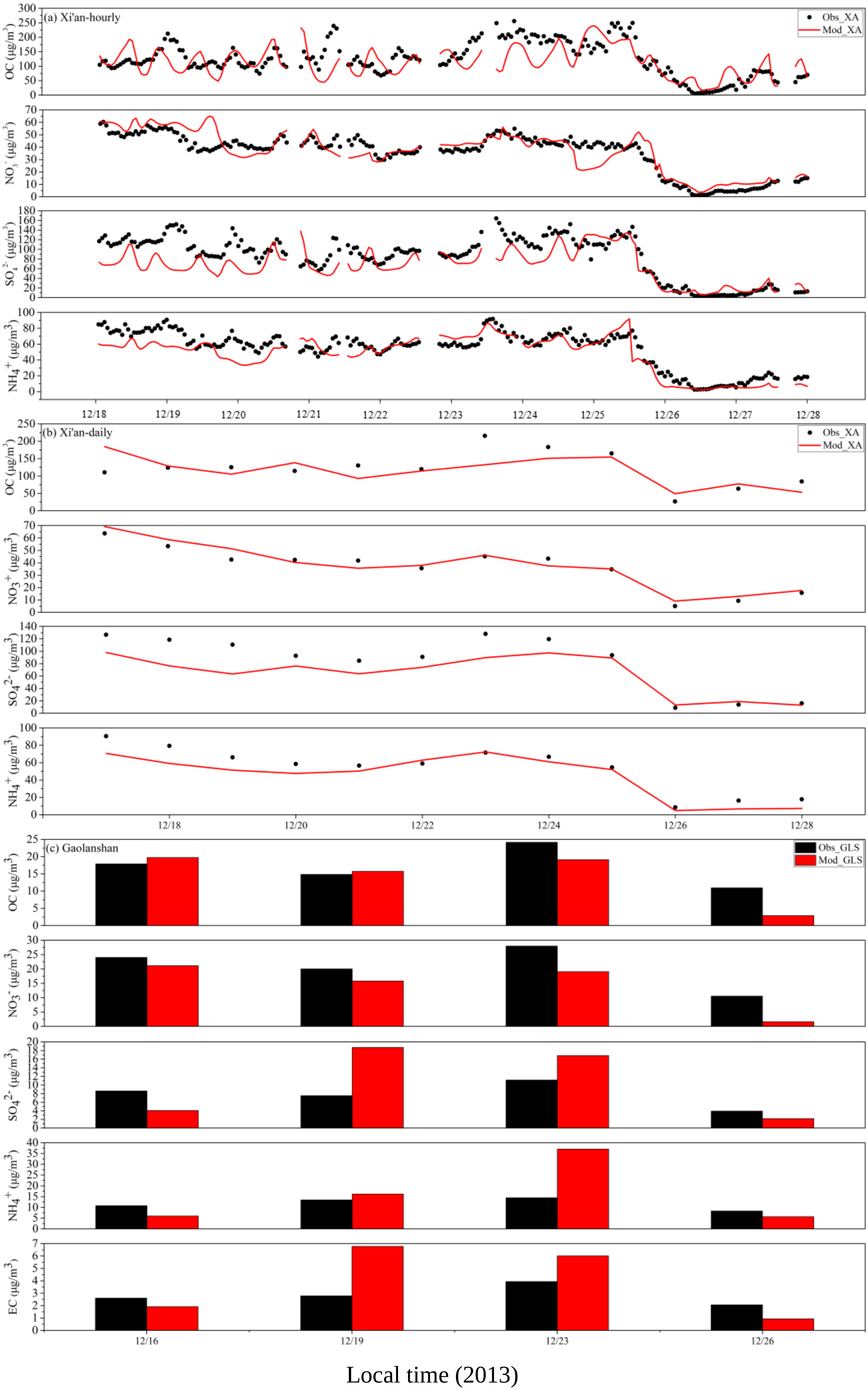

Fig 18. Evaluation of CMAQ model performance for $PM_{2.5}$ chemical composition at Xian ((a) hourly and (b) daily) and (c) Gaolanshan for Xian case.

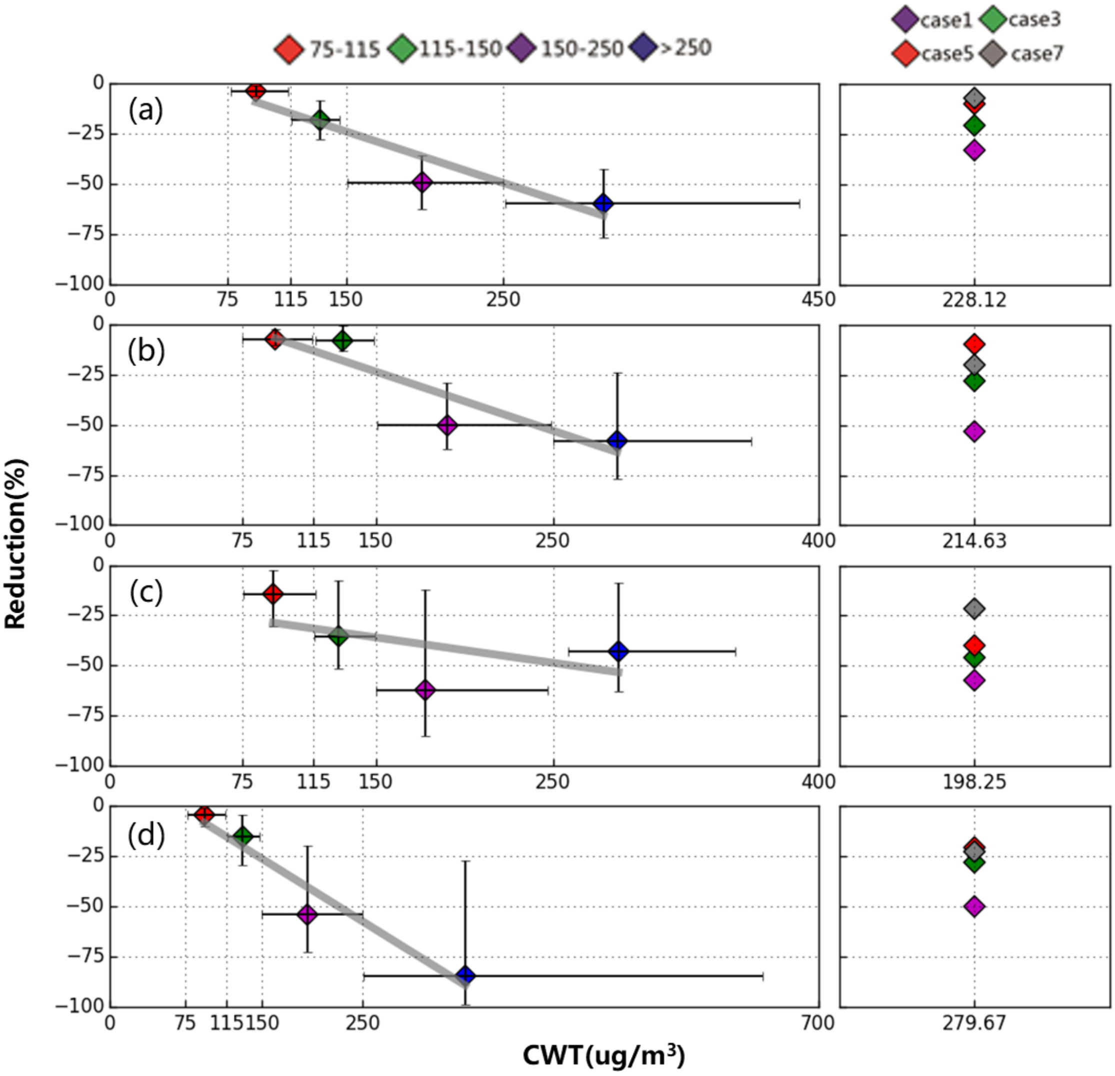

Fig. S19a Summary of the $PM_{2.5}$ reduction as a function of the CWT value intervals for $PM_{2.5}$. (a) The mean reductions as a function of the mean CWT values for the four emission control scenarios in Beijing for forecast application. The vertical and horizontal lines represent the ranges of the $PM_{2.5}$ reduction percentages and the CWT intervals, respectively. The points represent the mean values for each case. (b) The same as (a) but for Shanghai. (c) The same as (a) but for Hangzhou. (d) The same as (a) but for Xi'an. The right panels show the results for the cases 1, 3, 5, and 7 in Table 1.

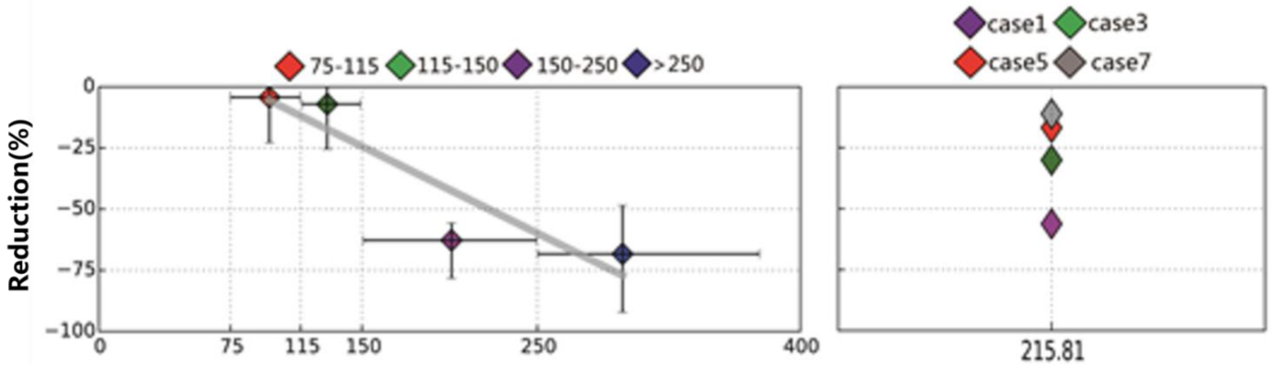

Fig. S19b. Summary of the $PM_{2.5}$ reduction as a function of the CWT value intervals for $PM_{2.5}$. The mean reductions as a function of the mean CWT values for the four emission control scenarios in Beijing (2013). The vertical and horizontal lines represent the ranges of the $PM_{2.5}$ reduction percentages and the CWT intervals, respectively. The points represent the mean values for each case. The right panels show the results for the cases 1, 3, 5, and 7 in Table 1.

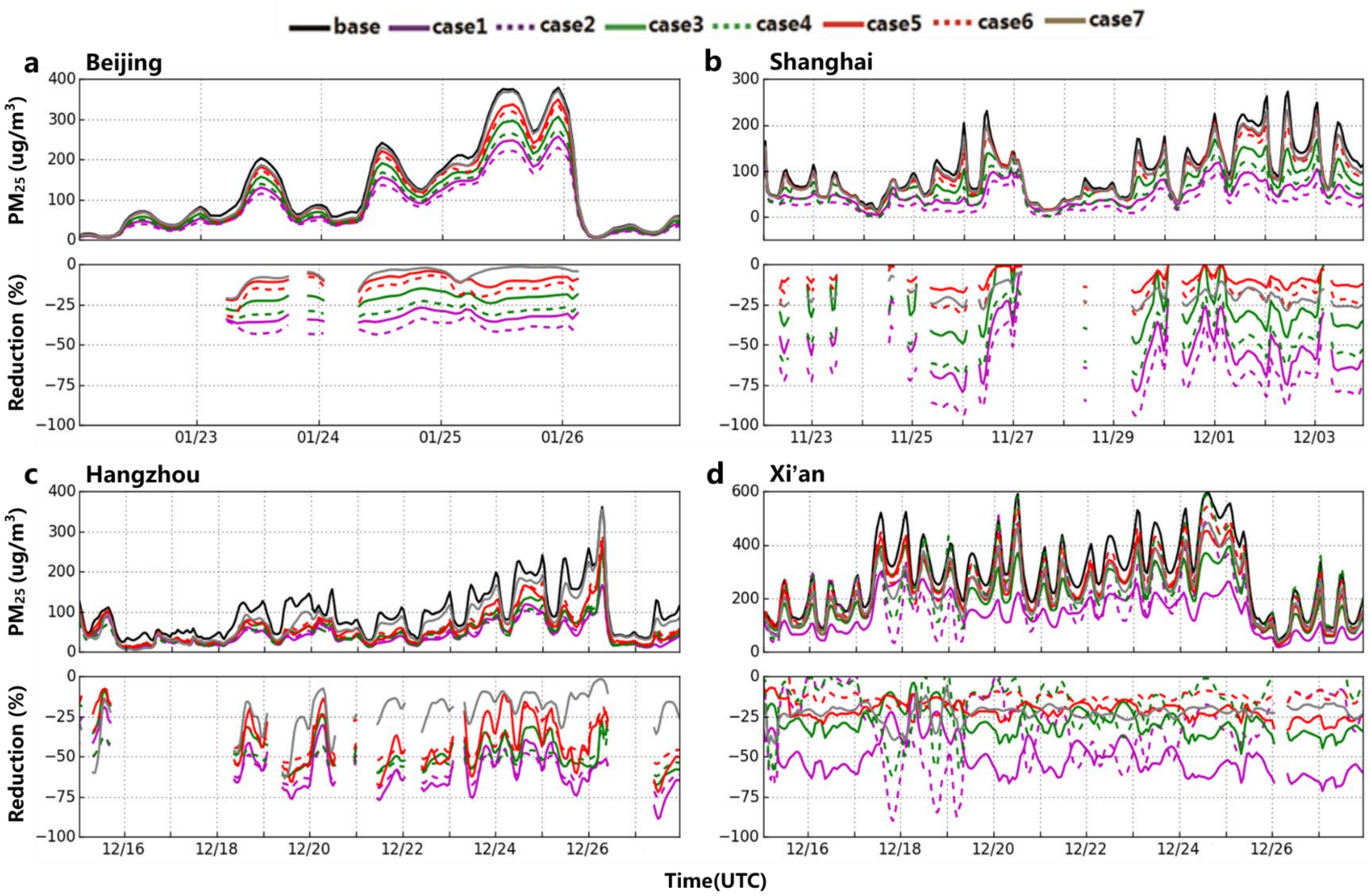

Fig. S20a Test of practicality of PAPCA for the different cases. (a) The temporal variations of the $PM_{2.5}$ concentration and its reductions relative to the base case for the six different emission control scenarios in Beijing for the period from Jan 22 to Jan 26, 2017. The reduced proportion is given only when the observation data exceed 75 μg m^{-3}. (b) The same as (a) but for the period from Nov 24 to Dec 4, 2013, in Shanghai. (c) The same as (a) but for the period from December 15 to Dec 28, 2013, in Hangzhou. (d) The same as (a) but for the period from December 15 to Dec 28, 2013, in Xian.

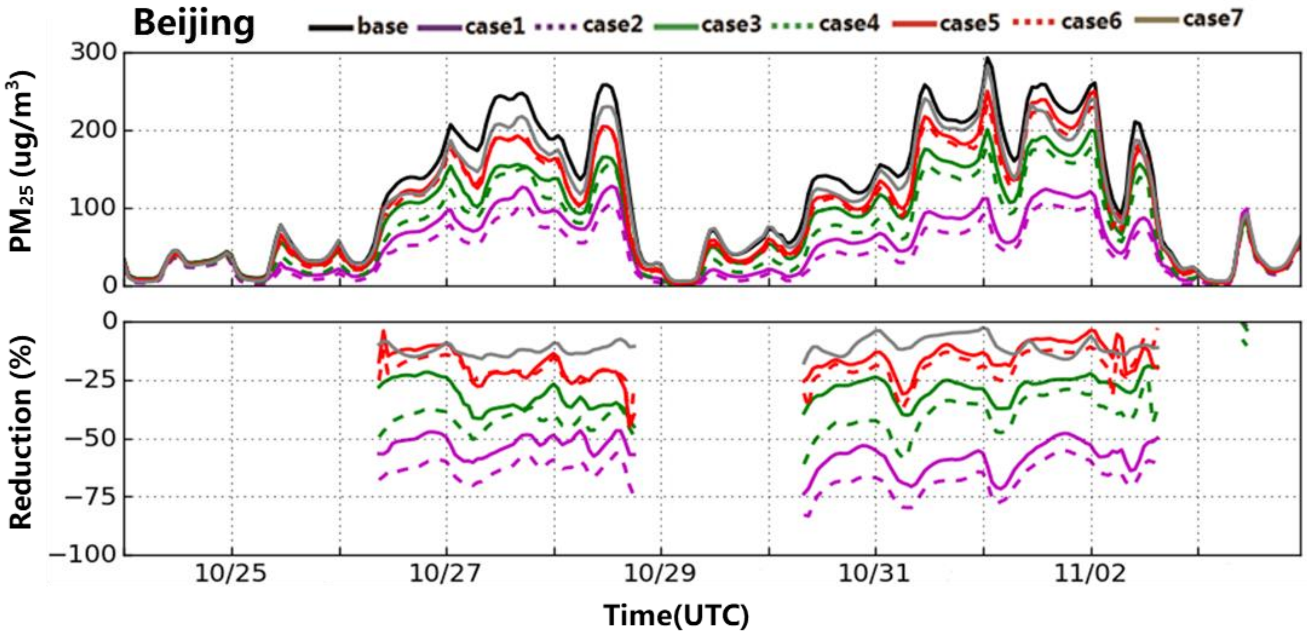

Fig. S20b Test of practicality of PAPCA for the different cases. The temporal variations of the $PM_{2.5}$ concentration and its reductions relative to the base case for the six different emission control scenarios in Beijing for the period from Dec 27 to Nov 3, 2013.The reduced proportion is given only when the observation data exceed 75 μgm^{-3}.

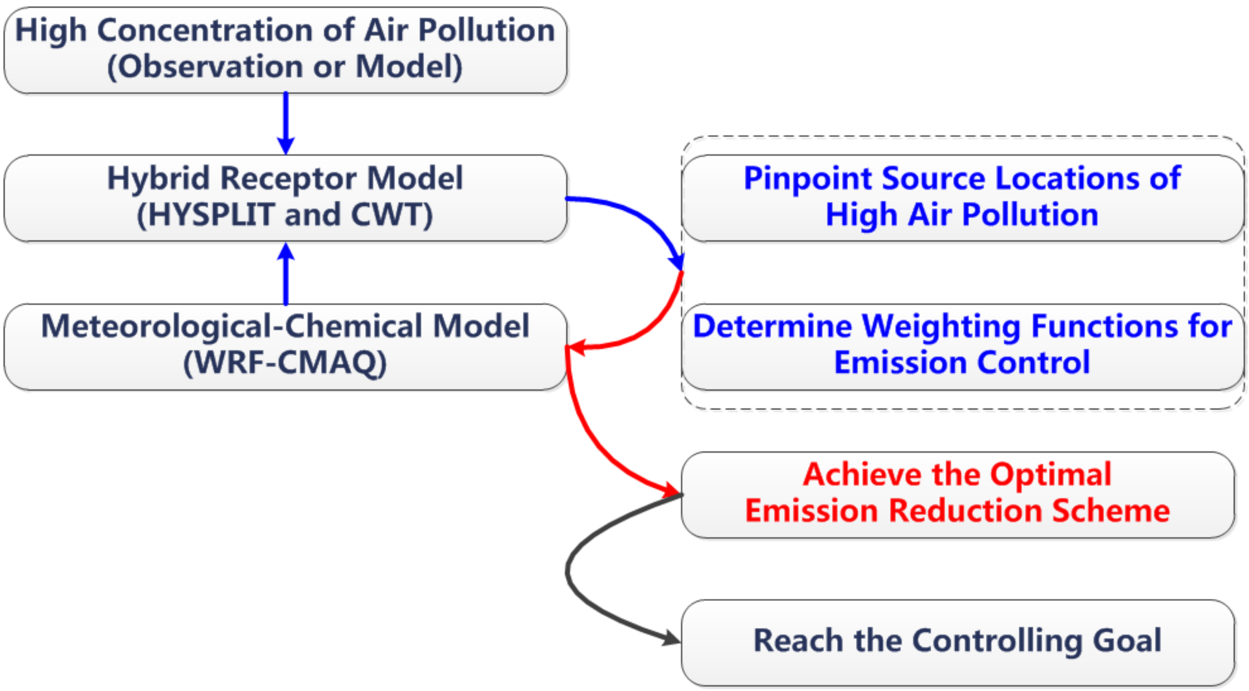

Fig. S21 The framework of the Precision Air Pollution Control Approach.

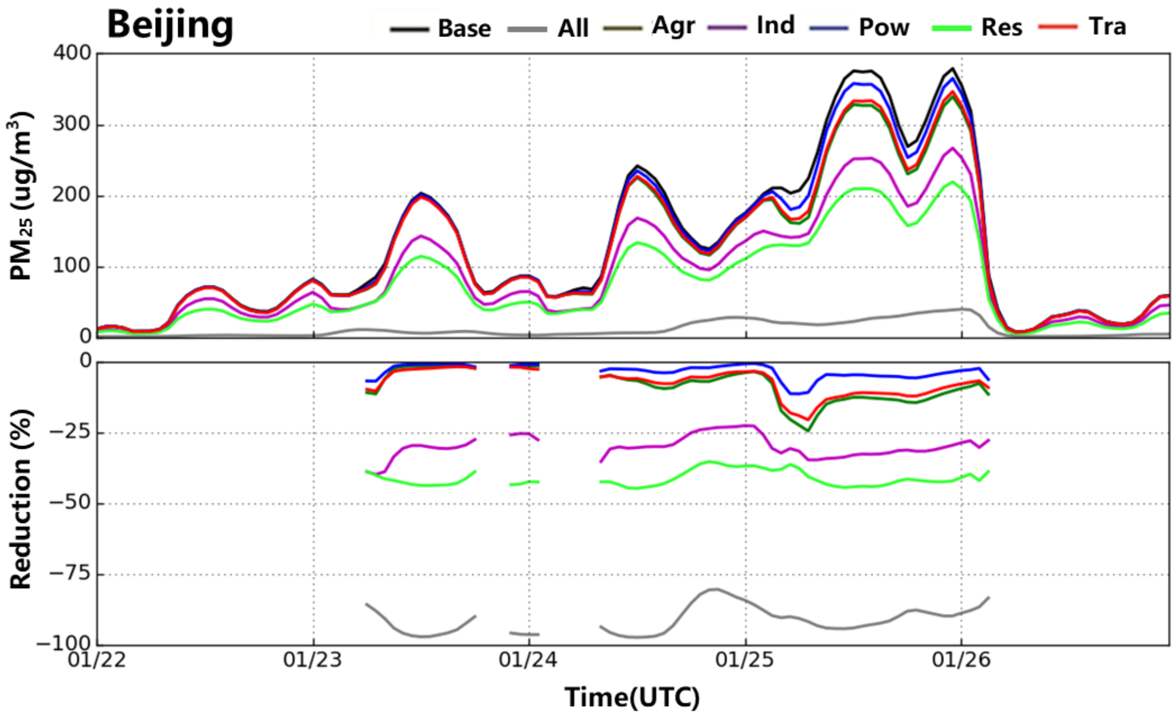

Fig. S21. Sensitivity analysis of anthropogenic emission sectors for Beijing case. The temporal variations of the $PM_{2.5}$ concentration and its reductions relative to Beijing base case for the six sensitivity analysis scenarios in Beijing for the period from Jan 22 to Jan 26, 2017.The reduced proportion is given only when the observation data exceed 75 μg m^{-3}. (All: all sectors, Agr: agriculture, Ind: industry, Pow: power, Res: residential, Tra: transportation.)

Table S1a. Information about the monitoring stations in each city from which the observational data are used to evaluate the model performance for each study case.

Study Case	City	Monitoring stations in city	Latitude	Longitude
Beijing Case	Beijing	Wanshouxigong	39.8673	116.3660
		Dingling	40.2865	116.1700
		Dongsi	39.9522	116.4340
		Tiantan	39.8745	116.4340
		Nongzhanguan	39.9716	116.4730
		Guanyuan	39.9425	116.3610
		Haidianquwanliu	39.9934	116.3150
		Shunyixincheng	40.1438	116.7200
		Huairouzhen	40.3937	116.6440
		Changpingzhen	40.1952	116.2300
		Aotizhongxi	40.0031	116.4070
		Gucheng	39.9279	116.2250
	Baoding	Youyongguan	38.8632	115.4930
		Huadianerqu	38.8957	115.5223
		Jiedaizhongxin	38.9108	115.4713
		Dibiaoshuichang	38.8416	115.4612
		Jiaopianchang	38.8756	115.4420
		Jiancezhan	38.8707	115.5214
	Cangzhou	Cangxianjiansheju	38.2991	116.8854
		Dianshizhuanbozhan	38.3254	116.8584
		Shihuanbaoju	38.3228	116.8709
	Chengde	Tielu	40.9161	117.9664
		Zhongguoyinhang	40.9843	117.9525
		Kaifaqu	40.9359	117.9630
		Wenhuazhongxin	40.9733	117.8184
		Ligong	41.0112	117.9384
	Handan	Huanbaoju	36.6176	114.5129
		Dongwushuichulichang	36.6164	114.5426
		Kuangyuan	36.5776	114.5035
		Congtaigongyuan	36.6198	114.4965
	Hengshui	Dianjibeichang	37.7575	115.6951
		Shijiancezhan	37.7379	115.6426
		Shihuanbaoju	37.7390	115.6906
	Langfang	Yaocaigongsi	39.5178	116.6838
		Kaifaqu	39.5747	116.7729
		Huanjingjiancejianlizhongxin	39.5571	116.7150
		Beihuahangtianxueyuan	39.5343	116.7464

Qinhuangdao	Beidaihehuanbaoju	39.8283	119.5259
	Diyiguan	40.0181	119.7624
	Jiancezhan	39.9567	119.6023
	Shizhengfu	39.9358	119.6070
	Jianshedasha	39.9419	119.5369
Shijiazhuang	Huagongxuexiao	38.0549	114.5637
	Zhigongyiyuan	38.0513	114.4548
	Gaoxinqu	38.0398	114.6046
	Xibeishuiyuan	38.1398	114.5019
	Xinangaojiao	38.0118	114.4671
	Shijigongyuan	38.0306	114.5483
	Renminhuitang	38.0524	114.5214
	fenglongshan	37.9097	114.3541
Tangshan	Gongxiaoshe	39.6308	118.1662
	Leidazhan	39.6430	118.1440
	Wuziju	39.6407	118.1853
	Taocigongsi	39.6679	118.2185
	Shierzhong	39.6578	118.1838
	Xiaoshan	39.6295	118.1997
Tianjin	Shijiancezhongxin	39.0970	117.1510
	Jichecheliangchang	39.1730	117.1930
	Jidianqichang	39.1654	117.1450
	Nanjinglu	39.1205	117.1840
	Hedongzhan	39.1082	117.2370
	Hexizhan	39.0927	117.2020
	Beichenkejiyuanqu	39.2261	117.1850
	Tiaoshanlu	39.1337	117.2690
	Donglizhongxue	39.0877	117.3070
	Meijiangxiaoqu	39.0600	117.2210
	Taifenggongyeyuan	39.0343	117.7070
	Yongminglu	38.8394	117.4570
	Konggangwuliujiagongqu	39.1240	117.4010
	Zhongxinshengtaicheng	39.1587	117.7640
	Tuanbowa	38.9194	117.1570
Xingtai	Dahuoquan	37.0967	114.4821
	Xingshigaozhuan	37.0533	114.5261
	Luqiaogongsi	37.0964	114.5331
	Shihuanbaoju	37.0620	114.4854
Zhangjiakou	Renmingongyuan	40.8367	114.8985
	Tanjichang	40.7948	114.8920
	Wujiku	40.8115	114.8814
	Shijihaoyuan	40.7688	114.9032

		Beibengfang	40.8725	114.9040
Shanghai Case	Shanghai	Putuo	31.2380	121.4000
		Shiwuchang	31.2036	121.4780
		Hongkou	31.3008	121.4670
		Xuhuishangshida	31.1654	121.4120
		Yangpusipiao	31.2659	121.5360
		Qingpudianshanhu	31.0935	120.9780
		Jinganjiancezhan	31.2261	121.4250
		Pudongchuansha	31.1907	121.7030
		Pudongxinqujiancezhan	31.2284	121.5330
		Pudongzhangjiang	31.2071	121.5770
	Changzhou	Shijiancezhan	31.7586	119.9960
		Chengjianxuexiao	31.7786	119.9330
		Changgongyuan	31.8089	119.9620
		Lucheng	31.7638	120.0393
		Wujinjiancezhan	31.7039	119.9350
		Anjia	31.9108	119.9050
	Hangzhou	Binjiang	30.2100	120.2110
		Xixi	30.2747	120.0630
		Xiasha	30.3058	120.3480
		Wolongqiao	30.2456	120.1270
		Zhejiangnongda	30.2692	120.1900
		Chaohuiwuqu	30.2897	120.1570
		Hemuxiaoxue	30.3119	120.1200
		Linpingzhen	30.4183	120.3010
		Chengxiangzhen	30.1819	120.2700
		Yunqi	30.1808	120.0880
	Huzhou	Chengbeishuichang	30.8867	120.1000
		Chengxishuichang	30.8244	120.0700
		Jishanxicun	30.8617	120.0930
	Jiaxing	Qinghexiaoxue	30.7946	120.7440
		Jiaxingxueyuan	30.7478	120.7260
		Jiancezhan	30.7567	120.7630
	Nanjing	Maigaoqiao	32.1083	118.8030
		Caochangmen	32.0572	118.7490
		Shanxilu	32.0723	118.7780
		Zhonghuamen	32.0144	118.7770
		Ruijinlu	32.0314	118.8030
		Xuanwuhu	32.0775	118.7950
		Pukou	32.0878	118.6260
		Aotizhongxin	32.0092	118.7370
		Xianlindaxuecheng	32.1050	118.9070
	Nantong	Nanjiao	31.9600	120.9130

		Hongqiao	32.0005	120.8600
		Chengzhong	32.0200	120.8700
		Xinghuhuayuan	31.9300	120.9400
		Zilangxueyuan	32.0417	120.8100
	Ningbo	Quhuanbaodalou	29.9108	121.8360
		Wanlixueyuan	29.8208	121.5600
		Longsaiyiyuan	29.9572	121.7130
		Sanjiangzhongxue	29.8906	121.5540
		Shizhengguanlizhan	29.9017	121.6150
		Qianhushuichang	29.7736	121.6330
		Taiguxiaoxue	29.8633	121.5860
		Shihuanjingjiancezhongxin	29.8506	121.5240
	Suzhou	Shangfangshan	31.2472	120.5610
		Nanmen	31.2864	120.6280
		Caixiang	31.3019	120.5910
		Zhagangchang	31.3264	120.5960
		Wuzhongqu	31.2703	120.6130
		Suzhouxinqu	31.2994	120.5430
		Suzhougongyeyuanqu	31.3097	120.6690
		Xiangchengqu	31.3708	120.6410
	Wuxi	Daxuecheng	31.4867	120.2690
		Shibeigaozhong	31.6219	120.2750
		Caozhang	31.5600	120.2940
		Qitang	31.5031	120.2420
		Dongtin	31.5848	120.3540
		Wangzhuang	31.5475	120.3540
		Yuhongxiaoxue	31.5631	120.2450
		Yanqiao	31.6842	120.2880
	Yangzhou	Chengdongcaizhengsuo	32.3878	119.4600
		Shijiancezhan	32.4100	119.4040
		Hanjiangjiancezhan	32.3761	119.3890
		Disiyiyuan	32.4033	119.4390
Hangzhou Case	Hangzhou	Binjiang	30.2100	120.2110
		Xixi	30.2747	120.0630
		Xiasha	30.3058	120.3480
		Wolongqiao	30.2456	120.1270
		Zhejiangnongda	30.2692	120.1900
		Chaohuiwuqu	30.2897	120.1570
		Hemuxiaoxue	30.3119	120.1200
		Linpingzhen	30.4183	120.3010
		Chengxiangzhen	30.1819	120.2700
		Yunqi	30.1808	120.0880
	Changzhou	Shijiancezhan	31.7586	119.9960

	Chengjianxuexiao	31.7786	119.9330
	Changgongyuan	31.8089	119.9620
	Lucheng	31.7638	120.0393
	Wujinjiancezhan	31.7039	119.9350
	Anjia	31.9108	119.9050
Huzhou	Chengbeishuichang	30.8867	120.1000
	Chengxishuichang	30.8244	120.0700
	Jishanxicun	30.8617	120.0930
Huaian	Bochishan	33.5981	119.0360
	Beijingnanlu	33.5750	119.0070
	Shijiancezhan	33.6067	118.9890
	Chuzhouqujiancezhan	33.5039	119.1350
	Huanyinqujiancezhan	33.6270	119.0122
Laiwu	Zhiwuyouchang	36.2050	117.6850
	Jishuxueyuan	36.2289	117.6789
	Rishengguoji	36.2081	117.7150
Lianyungang	Shihuanjingjiancezhan	34.5885	119.1760
	Hongmenpaichusuo	34.5896	119.1410
	Xugouhedianzhuanjiacun	34.7507	119.3680
	Kaifaquhengruiyiyaogongsi	34.6984	119.3480
Linyi	Yihexiaoqu	35.0573	118.3418
	Lunanzhiyaochang	35.0622	118.2939
	Xinguangmaofangchang	34.9817	118.2764
	Hedongbaoxiangongsi	35.0896	118.4023
Nanjing	Maigaoqiao	32.1083	118.8030
	Caochangmen	32.0572	118.7490
	Shanxilu	32.0723	118.7780
	Zhonghuamen	32.0144	118.7770
	Ruijinlu	32.0314	118.8030
	Xuanwuhu	32.0775	118.7950
	Pukou	32.0878	118.6260
	Aotizhongxin	32.0092	118.7370
	Xianlindaxuecheng	32.1050	118.9070
Yangzhou	Chengdongcaizhengsuo	32.3878	119.4600
	Shijiancezhan	32.4100	119.4040
	Hanjiangjiancezhan	32.3761	119.3890
	Disiyiyuan	32.4033	119.4390
Zhenjiang	Huanjingjiancezhan	32.2056	119.4290
	Xinqubanshichu	32.1883	119.6800
	Zhijiaozhongxin	32.2150	119.4910
	Dantuqujiancezhan	32.1319	119.4300

Xian Case	Xian	Gaoyakaiguanchang	34.2749	108.8820
		Xingqinxiaoqu	34.2629	108.9930
		Fangzhicheng	34.2572	109.0600
		Xiaozhai	34.2324	108.9400
		Shirenmintiyuchang	34.2713	108.9540
		Gaoxinxiqu	34.2303	108.8830
		Jingkaiqu	34.3474	108.9350
		Changanqu	34.1546	108.9060
		Yanliangqu	34.6575	109.2000
		Lintongqu	34.3731	109.2186
		Caotan	34.378	108.8690
		Qujiangwenhuachanyejituan	34.1978	108.9850
		Guangyuntan	34.3274	109.0430
	Baoji	Zhuyuangou	34.3017	107.0708
		Sanluyiyuan	34.3672	107.1906
		Jiancezhan	34.3547	107.1431
		Jigongxuexiao	34.3739	107.1186
		chencangquhuanbaoju	34.3528	107.3906
		Wenlixueyuan	34.3497	107.2058
		Sandixiaoxue	34.3622	107.2386
	Tongchuan	Dangxiao	35.0994	109.0656
		Wangyiquzhengfu	35.0697	109.0697
		Xinquguanweihui	34.9058	108.9344
		Xinqulanzhigongsi	34.8731	108.9589
	Weinan	Nongkesuo	34.5101	109.5293
		Ribaoshe	34.5004	109.5049
		Tiyuguan	34.493	109.4636
		Gaoxinyixiao	34.5021	109.4266
	Xianyang	Qixiangzhan	34.3956	108.7197
		Zhonghuaxiaoqu	34.3181	108.6761
		Shifanxueyuan	34.3617	108.7233
		Zhongyixueyuan	34.3164	108.7369
	Yanan	Zaoyuan	36.6275	109.4131
		Yandayifuyuan	36.6028	109.4761
		Shijiancezhan	36.5767	109.4824
		Baimidadao	36.6106	109.5056

Table S1b. Information about the monitoring stations in each city from which the observational chemical composition of $PM_{2.5}$ are used to evaluate the model performance for each study case.

Study case	monitoring station	Latitude	Longitude	Data Description
Beijing case	Beijing	39.9934	116.315	An Aerodyne high resolution time-of-flight aerosol mass spectrometer was used to measure the chemical composition of $PM_{2.5}$[6].
	Zhengzhou	34.78	113.68	The observed chemical composition of $PM_{2.5}$ are obtained from the Chinese Meteorological Administration Atmospheric Watch Network (CAWNET)[53]
Shanghai case	Linan	30.3	119.7333	The observed chemical composition of $PM_{2.5}$ are obtained from CAWNET[53]
	Taiyangshan	29.17	111.71	The observed chemical composition of $PM_{2.5}$ are obtained from CAWNET[53]
	Zhengzhou	34.78	113.68	The observed chemical composition of $PM_{2.5}$ are obtained from CAWNET[53]
Hangzhou case	Linan	30.3	119.7333	The observed chemical composition of $PM_{2.5}$ are obtained from CAWNET[53]
	Taiyangshan	29.17	111.71	The observed chemical composition of $PM_{2.5}$ are obtained from CAWNET[53]
	Zhengzhou	34.78	113.68	The observed chemical composition of $PM_{2.5}$ are obtained from CAWNET[53]
Xian case	Xian	34.43	108.97	The hourly and daily $PM_{2.5}$ chemical composition is measured by the Aerodyne High Resolution Time-of-Flight Aerosol Mass Spectrometer (HR-ToF-AMS) with a novel $PM_{2.5}$ lens[54]
	Gaolanshan	36	105.85	The observed chemical composition of $PM_{2.5}$ are obtained from CAWNET[53]

Table S2a. Summary of normalized mean bias (NMB) (%) for model performance for $PM_{2.5}$, PM_{10}, O_3, SO_2, NO_2, and CO at Beijing and its surrounding 10 cities for the Beijing case (2017 forecast case)

Cities	$PM_{2.5}$	PM_{10}	O_3	SO_2	NO_2	CO
Beijing	8.99	-0.22	-32.97	54.72	14.58	-0.47
Baoding	-12.28	-21.85	-31.61	21.39	1.83	-14.62
Cangzhou	11.95	-4.16	-30.38	11.17	11.4	-12.73
Chengde	-20.38	28.36	30.11	-20.6	-18.87	-31.09
Handan	-12.8	-37.55	-31.77	-10.41	-9.94	-24.33
Hengshui	-8.38	-30.65	-9.1	20.7	0.22	-25.59
Langfang	6.75	-7.35	-21.29	62.96	4.01	-27.22
Qinhuangdao	-7.93	-23.09	28.98	-9.44	-17.73	-36.19
Shijiazhuang	-8.59	-22.9	-47.34	27.95	15.25	-13.54
Tangshan	-4.49	-12.02	-8.98	18.75	-9.92	-19.98
Tianjin	31.18	21.02	-39.63	65.37	8.22	-10.73

Table S2b. Summary of model performance for $PM_{2.5}$, PM_{10}, O_3, SO_2, NO_2, and CO at Beijing for the Beijing case (2017 forecast)

Species	Obs*	Model*	R	MB*	NMB (%)	ME	NME (%)	N pairs
$PM_{2.5}$	115.26	125.62	0.83	10.36	8.99	45.97	42.23	136
PM_{10}	141.87	141.56	0.83	-0.31	-0.22	56.05	41.83	136
CO	1.84	1.83	0.84	-0.10	-0.47	0.48	27.44	136
NO_2	59.17	67.80	0.80	8.62	14.58	14.89	26.64	136
O_3	30.67	20.56	0.57	-10.11	-32.97	15.48	53.44	136
SO_2	28.33	43.83	0.88	15.50	54.72	15.25	57.00	136

*The units of Obs, Mod, MB and ME for CO are mg m^{-3}, for other species are μg m^{-3}.

Table S2c. Summary of normalized mean bias (NMB) (%) for model performance for $PM_{2.5}$, PM_{10}, O_3, SO_2, NO_2, and CO at Beijing and its surrounding 10 cities for the Beijing case (2013 case)

Cities	$PM_{2.5}$	PM_{10}	O_3	SO_2	NO_2	CO
Beijing	2.75	2.75	17.26	14.10	4.20	-21.06
Baoding	-10.68	-10.68	6.97	14.19	-28.82	0.82
Cangzhou	5.64	5.64	-10.47	-17.07	-30.57	-16.84
Chengde	-6.86	-47.74	30.71	-18.86	-49.27	9.33
Handan	0.22	0.22	17.57	-19.97	-28.24	-5.00
Hengshui	-10.87	-10.87	-6.75	-36.15	-25.95	-6.93
Langfang	-6.75	-6.75	42.30	20.23	-0.79	9.87
Qinhuangdao	-19.95	-51.73	42.86	-50.26	-37.36	-33.76
Shijiazhuang	-0.61	-0.61	-17.40	-15.40	-9.48	-12.90
Tangshan	45.80	45.80	8.24	-4.26	-15.79	-21.05
Tianjin	1.27	-23.88	13.06	5.26	-2.39	-1.44

Table S2d. Summary of model performance for $PM_{2.5}$, PM_{10}, O_3, SO_2, NO_2, and CO at Beijing for the Beijing case (2013 case)

Species	Obs*	Model*	R	MB*	NMB (%)	ME	NME (%)	N pairs
CO	1.42	1.12	0.59	-0.29	-21.06	0.52	38.68	274
SO_2	13.90	15.85	0.45	1.95	14.10	8.46	63.97	274
NO_2	77.40	80.64	0.68	3.25	4.20	22.17	30.13	274
$PM_{2.5}$	112.24	115.32	0.79	3.08	2.75	38.20	34.28	286
PM_{10}	137.78	137.63	0.76	-0.15	-0.11	45.23	33.77	280
O_3	22.54	26.43	0.75	3.89	17.26	7.83	62.10	161

*The units of Obs, Mod, MB and ME for CO are mg m^{-3}, for other species are μg m^{-3}.

Table S3a.Summary of normalized mean bias (NMB, %) for model performance for $PM_{2.5}$, PM_{10}, O_3, SO_2, NO_2, and CO at Shanghai and its surrounding 10 cities for the Shanghai case.

Cities	$PM_{2.5}$	PM_{10}	O_3	SO_2	NO_2	CO
Shanghai	-14.48	-14.48	9.66	11.07	-18.11	-15.20
Changzhou	5.59	5.59	18.70	-29.03	-5.50	-6.93
Hangzhou	-8.34	-8.34	25.87	8.26	-19.94	6.53
Huzhou	-25.30	-25.30	54.89	-26.12	-41.80	-2.95
Jiaxing	-22.92	-22.92	49.68	-36.93	-33.05	-34.90
Nanjing	-6.76	-6.76	-4.28	51.49	13.97	0.53
Nantong	-6.88	-6.88	10.36	-24.95	-1.92	-47.32
Ningbo	-26.24	-26.24	45.57	-19.36	-38.78	-34.39
Suzhou	19.73	19.73	3.38	56.05	-5.50	-47.49
Wuxi	7.01	7.01	35.15	-18.16	7.59	-25.34
Yangzhou	17.46	17.46	27.22	-12.31	15.98	-15.52

Table S3b. Summary of model performance for $PM_{2.5}$, PM_{10}, O_3, SO_2, NO_2, and CO at Shanghai for the Shanghai case.

Species	Obs*	Model*	R	MB*	NMB (%)	ME	NME (%)	N pairs
CO	1,38	1.17	0.71	-0.21	-15.51	0.40	29.05	304
SO_2	48.97	60.45	0.69	11.48	23.43	20.66	42.18	304
NO_2	73.95	70.48	0.71	-3.47	-4.69	18.77	25.39	304
$PM_{2.5}$	106.88	103.11	0.74	-3.77	-3.53	33.75	31.58	305
PM_{10}	159.42	115.95	0.62	-43.47	-27.26	60.58	33.00	305
O_3	38.56	34.38	0.71	-4.17	-10.82	19.22	49.84	304

*The units of Obs, Mod, MB and ME for CO are mg m^{-3}, for other species are μg m^{-3}.

Table S4a. Summary of normalized mean bias (NMB, %) for model performance for $PM_{2.5}$, PM_{10}, O_3, SO_2, NO_2, and CO at Hangzhou and its surrounding 9 cities for the Hangzhou case.

Cities	$PM_{2.5}$	PM_{10}	O_3	SO_2	NO_2	CO
Hangzhou	-11.38	-11.38	26.43	-4.20	-12.82	-5.21
Changzhou	10.84	10.84	6.51	-15.47	11.66	-38.38
Huaian	-35.87	-35.87	-15.40	-48.74	-19.77	-55.48
Huzhou	-37.12	-37.12	42.21	-34.99	-34.00	6.58
Laiwu	-23.29	-23.29	45.60	-23.42	-29.65	16.06
Lianyungang	-45.78	-45.78	41.59	-48.63	-43.96	-22.15
Linyi	-25.51	-25.51	34.92	-20.00	-28.78	8.95
Nanjing	-7.07	-7.07	3.63	-26.77	-19.51	3.69
Yangzhou	-23.91	-23.91	44.06	-46.11	-4.27	-26.48
Zhenjiang	-12.08	-12.08	43.83	-23.88	-21.83	-27.96

Table S4b. Summary of model performance for $PM_{2.5}$, PM_{10}, O_3, SO_2, NO_2, and CO at Hangzhou for the Hangzhou case.

Species	Obs*	Model*	R	MB*	NMB (%)	ME	NME (%)	N pairs
CO	1.59	1.50	0.62	-0.08	-5.21	0.62	25.44	327
SO_2	43.39	41.57	0.61	-1.82	-4.20	0.23	32.99	327
NO_2	72.88	63.53	0.60	-9.34	-12.82	1.37	25.32	327
$PM_{2.5}$	112.25	99.47	0.58	-12.77	-11.40	30.60	38.28	330
PM_{10}	149.14	118.92	0.58	-30.22	-20.26	15.64	36.07	315
O_3	19.75	24.97	0.69	5.22	26.43	1.53	67.60	327

*The units of Obs, Mod, MB and ME for CO are mg m^{-3}, for other species are μg m^{-3}.

Table S5a. Summary of normalized mean bias (NMB, %) for model performance for $PM_{2.5}$, PM_{10}, O_3, SO_2, NO_2, and CO at Xian and its surrounding 5 cities for the Xian case.

Cities	$PM_{2.5}$	PM_{10}	O_3	SO_2	NO_2	CO
Xian	-11.13	-11.13	-26.68	12.83	-0.01	3.94
Baoji	-32.53	-32.53	50.45	16.12	-24.28	-33.90
Tongchuan	-37.10	-37.10	1.26	0.16	-14.76	-4.99
Weinan	-26.90	-26.90	57.40	26.18	-24.12	-13.85
Xianyang	20.10	20.10	-11.84	62.50	-12.75	-15.94
Yanan	-18.24	-18.24	33.02	-57.61	-12.19	-22.00

Table S5b. Summary of model performance for $PM_{2.5}$, PM_{10}, O_3, SO_2, NO_2, and CO at Xian for the Xian case.

Species	Obs*	Model*	R	MB*	NMB (%)	ME	NME (%)	N pairs
CO	3.76	3.82	0.67	0.06	1.67	1.11	29.67	325
SO_2	86.85	95.48	0.45	8.62	9.93	35.74	41.15	325
NO_2	76.47	78.11	0.69	1.64	2.14	16.27	21.28	325
$PM_{2.5}$	299.67	255.19	0.82	-44.47	-14.84	97.06	32.39	330
PM_{10}	417.29	275.68	0.83	-141.62	-33.93	170.05	40.75	330
O3	13.12	11.93	0.51	-1.20	-9.13	10.41	79.34	325

*The units of Obs, Mod, MB and ME for CO are mg m^{-3}, for other species are μg m^{-3}.

Table S6a. **Summary of model performance for $PM_{2.5}$ chemical composition for the Beijing case (2013 case).**

Site	Species	Obs*	Model*	R	MB*	NMB (%)	ME	NME (%)	N pairs
Beijing	EC	2.58	2.85	0.49	0.27	10.29	0.65	25.23	7
	OC	31.01	16.78	0.55	-14.22	-45.87	17.04	54.94	7
	NO_3^-	30.74	36.53	0.92	5.79	18.84	10.83	35.23	8
	SO_4^{2-}	17.59	12.23	0.94	-5.35	-30.44	5.67	32.27	8
	NH_4^+	17.55	14.14	0.91	-3.41	-19.42	4.07	23.21	8
Zhengzhou	EC	7.26	7.01	0.99	-0.26	-3.54	1.52	20.96	3
	OC	32.11	26.37	0.99	-5.75	-17.89	5.75	17.89	3
	NO_3^-	34.08	24.34	0.82	-9.75	-28.6	9.74	28.6	3
	SO_4^{2-}	13.1	11.13	0.97	-1.96	-14.99	1.96	14.99	3
	NH_4^+	28.15	23.51	0.81	-4.63	-16.46	6.39	22.69	3

*The units of Obs, Mod, MB and ME are μg m^{-3}. The unit of OC is μgC m^{-3}.

Table S6b. Summary of model performance for $PM_{2.5}$ chemical composition for the Shanghai case.

Site	Species	Obs*	Model*	R	MB*	NMB (%)	ME	NME	N pairs
Linan	EC	4.05	4.46	0.88	0.41	10.22	1.48	36.6	4
	OC	16.73	8.86	0.94	-7.87	-47.04	7.87	47.04	4
	NO_3^-	25.32	19.83	0.94	-5.48	-21.66	5.48	21.66	4
	SO_4^{2-}	7.24	8.86	0.75	1.62	22.44	3.29	45.46	4
	NH_4^+	10.99	18.08	0.83	7.1	64.58	7.1	64.58	4
Taiyangshan	EC	3.74	6.94	0.99	3.2	85.61	3.2	85.61	3
	OC	18.76	21.27	0.34	2.5	13.33	2.5	13.33	3
	NO_3^-	30.32	27.02	0.99	-3.3	-10.88	3.3	10.88	3
	SO_4^{2-}	12.55	16.42	0.75	3.87	30.83	3.87	30.83	3
	NH_4^+	20.95	23.05	0.96	2.1	10.04	2.34	11.19	3
Zhengzhou	EC	16.48	21.53	0.98	5.05	30.64	5.05	30.64	3
	OC	69.97	88.06	0.99	-14.91	-21.31	14.91	21.31	3
	NO_3^-	77.51	49.35	0.94	-28.16	-36.33	28.16	36.33	3
	SO_4^{2-}	25.6	22.8	0.99	-2.8	-10.94	5.59	21.83	3
	NH_4^+	39.93	31.42	0.98	-8.51	-21.31	8.51	21.31	3

*The units of Obs, Mod, MB and ME are $\mu g\ m^{-3}$. The unit of OC is $\mu gC\ m^{-3}$.

Table S6c. Summary of model performance for $PM_{2.5}$ chemical composition for the Hangzhou case.

Site	Species	Obs*	Model*	R	MB*	NMB (%)	ME	NME (%)	N pairs
Linan	EC	2.17	2.93	0.99	0.77	35.37	0.77	35.37	3
	OC	12.53	7.74	0.99	-4.79	-38.24	4.79	38.24	3
	NO_3^-	12.3	6.87	0.91	-5.43	-44.19	5.43	44.19	3
	SO_4^{2-}	2.55	5.03	0.96	2.49	97.61	2.49	97.62	3
	NH_4^+	9.04	12.61	0.97	3.57	39.44	3.57	39.44	3
Taiyangshan	EC	1.45	3.08	-0.96	1.64	113.32	1.64	113.32	3
	OC	7.74	7.33	0.68	-0.41	-5.28	2.03	26.24	3
	NO_3^-	13.13	8.87	0.88	-4.26	-32.45	4.26	32.45	3
	SO_4^{2-}	3.46	4.96	0.94	1.5	43.51	1.5	43.51	3
	NH_4^+	4.77	8.4	0.18	3.63	76.04	3.63	76.04	3
Zhengzhou	EC	9.98	11.78	0.99	1.8	18	2.34	23.49	3
	OC	24.69	15.62	0.97	-9.08	-36.76	9.76	39.53	3
	NO_3^-	19.42	11.07	0.99	-8.34	-42.96	8.34	42.96	3
	SO_4^{2-}	7.07	5.21	0.99	-1.87	-26.4	2.06	29.18	3
	NH_4^+	14.83	8.72	0.99	-6.11	-41.19	6.62	44.66	3

*The units of Obs, Mod, MB and ME are μg m^{-3}. The unit of OC is μgC m^{-3}.

Table S6d. Summary of model performance for $PM_{2.5}$ chemical composition for the Xian case.

Site	Species	Obs*	Model*	R	MB*	NMB (%)	ME	NME (%)	N pairs
Xian-daily	OC	121.91	115.19	0.65	-55.49	-32.51	62.42	36.57	12
	NO_3^-	36.07	37.62	0.97	1.55	4.3	3.88	10.76	12
	SO_4^{2-}	83.6	64.38	0.95	-19.22	-22.99	20.81	24.9	12
	NH_4^+	53.82	45.56	0.96	-8.27	-15.36	9.06	16.83	12
Xian-hourly	OC	126.70	113.55	0.55	-63.83	-35.98	73.52	41.45	235
	NO_3^-	35.32	36.56	0.88	1.24	3.52	6.05	17.13	235
	SO_4^{2-}	85.22	65.19	0.8	-20.03	-23.5	26.03	30.54	235
	NH_4^+	53.4	46.06	0.89	-7.34	-13.75	10.96	20.52	235
Gaolanshan	EC	2.85	3.91	0.72	1.06	37.21	1.97	69.2	4
	OC	16.96	11.52	0.63	-5.44	-32.08	5.85	34.49	4
	NO_3^-	20.63	14.42	0.94	-6.21	-30.11	6.21	30.11	4
	SO_4^{2-}	7.81	10.46	0.6	2.66	34.03	5.79	74.11	4
	NH_4^+	11.75	16.22	0.84	4.48	38.14	8.19	69.68	4

*The units of Obs, Mod, MB and ME are μg m^{-3}. The unit of OC is μgC m^{-3}.

Table S7. $PM_{2.5}$ reduction and emission control amounts for each species for each case (Beijing-2013)

Case	$PM_{2.5}$ reduction (%)	Emission reduction (10^7kg)								
		CO	SO_2	NH_3	NO_X	VOC	$PM_{2.5}$	PMcoarse	BC	OC
					Beijing					
1	-56.2	23.7	24.6	0.4	30.2	27.4	12.4	6.3	1.7	1.5
2	-59.6	30.9	31.7	0.4	40.1	36.9	16.7	8.5	2.2	2.0
3	-30.1	16.4	16.5	0.2	23.6	19.0	8.5	4.2	1.3	1.1
4	-37.2	21.6	21.3	0.3	30.6	25.5	11.4	5.7	1.6	1.4
5	-16.8	9.3	8.4	0.2	16.9	10.8	4.6	2.1	0.8	0.6
6	-19.7	12.2	10.8	0.2	21.1	14.2	6.1	2.8	1.0	0.8
7	-11.1	4.5	0.3	0.1	0.9	1.2	0.3	0.1	0.06	0.09

Table S8. Mean $PM_{2.5}$ concentrations and percentages of trajectories (The values in the parentheses are number of back trajectories) for each trajectory cluster for four cases. The "Polluted Percent" is calculated on the basis of number of trajectories for the trajectory cluster such as NW with $PM_{2.5}$ concentration > 75 μg m^{-3} divided by the total trajectories from the trajectory cluster. The values of the pressure and height of each trajectory cluster at 48-h earlier before arriving at the receptor sites are also listed. "Beijing case-F" and "Beijing case-2013" represent the results for the periods of Jan 24-26, 2017 and Oct 27 to Nov 3, 2013, respectively.

	Clusters	Percent (%)	Mean $PM_{2.5}$ (μg m^{-3})	Polluted Percent (%)	Polluted Mean $PM_{2.5}$ (μg m^{-3})	Pressure (-48h)(hPa)	Height (m)
**Beijing case-F**							
1	W	25%(67)	98.09	23%(40)	139.09	764.62	2284.21
2	NW	20%(55)	24.75	1%(1)	83.57	631.85	3802.35
3	SW	55%(149)	201.51	77%(136)	216.12	938.50	642.86
All		100%(271)	14.07	100%(177)	197.97	-	-
**Beijing case-2013**							
1	E-SW	49% (261)	201.55	61% (250)	208.04	954.03	449.95
2	NW	21% (113)	41.32	4% (17)	98.94	733.95	2691.44
3	SW	29% (155)	187.55	35% (145)	199.01	1002.72	24.51
All		100% (529)	163.02	100% (412)	200.36	-	-
**Shanghai case**							
1	NW	57% (251)	132.08	50% (159)	175.36	761.77	2373.46
2	NW-W	38% (168)	132.18	44% (142)	144.23	828.89	1651.73
3	NW-S	5% (20)	124.75	6% (19)	127.42	884.74	1094.41
All		100% (439)	131.78	100% (320)	158.7	-	-
**Hangzhou case**							
1	NE	8% (61)	40.49	0% (0)	0	931.89	650.64
2	NW	58% (459)	128.19	72% (355)	149.95	847.46	1462.36
3	N	34% (270)	79.88	28% (135)	110.49	871.4	1224.26
All		100% (790)	104.91	100% (490)	139.08	-	-
**Xian case**							
1	N	33% (336)	332.81	32% (315)	352.06	812.23	1825.27
2	NW	24% (252)	214.15	24% (234)	225.66	773.96	2237.77
3	SE	20% (204)	344.17	21% (204)	344.17	964.07	360.48
4	E	23% (240)	381.66	24% (240)	381.66	927.53	690.72
All		100% (1032)	317.44	100% (993)	327.81	-	-

Table S9. Contributions of different anthropogenic emission sectors over the Beijing-Tianjing-Hebei region to $PM_{2.5}$ concentrations in Beijing on the basis of the model forecast simulations from Jan 24-26, 2017.

Contribution (%)	All	Agr	Ind	Pow	Res	Tra
Mean	91.53%	5.65%	28.71%	2.97%	40.90%	5.54%
Min	78.93%	0.04%	19.67%	0.20%	32.96%	1.04%
Max	97.37%	24.56%	42.51%	11.41%	44.79%	20.61%

Table S10. The comparison of $PM_{2.5}$ reduction percentages of six emission control scenarios of Beijing-Forecast case and Beijing-Observation case. (Case1, 3, 5-F: three scenarios of Beijing-Forecast case, Case1, 3, 5-O: three scenarios of Beijing-Observation case) (see Table 1)

$PM_{2.5}$ Reduction (%)	Case1-F	Case1-O	Case3-F	Case3-O	Case5-F	Case5-O
Mean	-33.0%	-32.6%	-20.7%	-20.4%	-9.9%	-9.8%
Min	-26.9%	-24.1%	-15.0%	-13.4%	-3.4%	-3.4%
Max	-38.2%	-38.0%	-30.1%	-29.9%	-23.4%	-23.2%
